# Multifunctionality and diversity of GDSL esterase/lipase gene family in rice (*Oryza sativa* L. *japonica*) genome: new insights from bioinformatics analysis

**DOI:** 10.1186/1471-2164-13-309

**Published:** 2012-07-15

**Authors:** Hanna Chepyshko, Chia-Ping Lai, Li-Ming Huang, Jyung-Hurng Liu, Jei-Fu Shaw

**Affiliations:** 1 Department of Food Science and Biotechnology, National Chung Hsing University, Taichung, Taiwan, 402, ROC; 2 Department of Food and Beverage Management, Far East University, Tainan, Taiwan, 74448, ROC; 3 Institute of Biotechnology, National Cheng Kung University, Tainan, Taiwan, 701, ROC; 4 Institute of Genomics and Bioinformatics, National Chung Hsing University, Taichung, Taiwan, 40227, ROC; 5 Department of Biological Science and Technology, I-Shou University, Kaohsiung, Taiwan, 84001, ROC; 6 Agricultural Biotechnology Center, National Chung Hsing University, Taichung, Taiwan, 40227, ROC; 7 Agricultural Biotechnology Research Center, Academia Sinica, Nankang, Taiwan, 115, ROC

## Abstract

**Background:**

GDSL esterases/lipases are a newly discovered subclass of lipolytic enzymes that are very important and attractive research subjects because of their multifunctional properties, such as broad substrate specificity and regiospecificity. Compared with the current knowledge regarding these enzymes in bacteria, our understanding of the plant GDSL enzymes is very limited, although the GDSL gene family in plant species include numerous members in many fully sequenced plant genomes. Only two genes from a large rice GDSL esterase/lipase gene family were previously characterised, and the majority of the members remain unknown. In the present study, we describe the rice *OsGELP* (*Oryza sativa* GDSL esterase/lipase protein) gene family at the genomic and proteomic levels, and use this knowledge to provide insights into the multifunctionality of the rice OsGELP enzymes.

**Results:**

In this study, an extensive bioinformatics analysis identified 114 genes in the rice *OsGELP* gene family. A complete overview of this family in rice is presented, including the chromosome locations, gene structures, phylogeny, and protein motifs. Among the OsGELPs and the plant GDSL esterase/lipase proteins of known functions, 41 motifs were found that represent the core secondary structure elements or appear specifically in different phylogenetic subclades. The specification and distribution of identified putative conserved clade-common and -specific peptide motifs, and their location on the predicted protein three dimensional structure may possibly signify their functional roles. Potentially important regions for substrate specificity are highlighted, in accordance with protein three-dimensional model and location of the phylogenetic specific conserved motifs. The differential expression of some representative genes were confirmed by quantitative real-time PCR. The phylogenetic analysis, together with protein motif architectures, and the expression profiling were analysed to predict the possible biological functions of the rice *OsGELP* genes.

**Conclusions:**

Our current genomic analysis, for the first time, presents fundamental information on the organization of the rice *OsGELP* gene family. With combination of the genomic, phylogenetic, microarray expression, protein motif distribution, and protein structure analyses, we were able to create supported basis for the functional prediction of many members in the rice GDSL esterase/lipase family. The present study provides a platform for the selection of candidate genes for further detailed functional study.

## Background

The GDSL motif enzyme is a relatively newly discovered lipase, with many characteristics that have not yet been fully, clearly, and precisely described [1,2]. Since 1995, when Upton and Buckley first reported the new GDS[L]-motif-like subfamily of lipases (pfam PF00657), new questions have arisen about the specific functions of these fascinating lipolytic enzymes.

The number of lipases (EC 3.1.1.3) and esterases (EC 3.1.1.1) that have been studied tremendously increased over the last decades. The lipase and esterase families belong to hydrolases—a class of enzymes that shows very broad substrate specificity. All enzymes in these families contained a catalytic triad composed of serine (Ser), aspartic (or glutamic), and histidine (His) residues. The role of the nucleophile in lipases is played by a Ser residue, which is a part of the highly conserved motif Gly-X-Ser-X-Gly (X being any amino acid), positioned in the middle of the amino acid sequence. In contrast, enzymes that belong to the GDSL family of esterases/lipases share five blocks of highly conserved homology, which are important for their classification. The active-site Ser is located close to the N-terminus. The GDSL family is further classified as SGNH hydrolase because of the presence of the strictly conserved residues Ser-Gly-Asn-His in the conserved blocks I, II, III, and V [1-3]. Two other proton donors to the oxidation hole are the glycine (Gly) residue in block II and the asparagine (Asn) in block III. The His amino acid in block V serves as a base that makes the Ser in block I more nucleophilic by deprotonating the hydroxyl group. Additional characteristic for block V is the presence of aspartate (Asp) three amino acids ahead of His (i.e., DxxH sustain as the third member of the catalytic triad). Unlike other lipases, GDSL hydrolases have a flexible active site and they change conformation in the presence of different substrates; hence, some GDSL enzymes have broadly diverse enzymatic activities, including esterase and protease activity in the same enzyme [4,5].

The GDSL esterases/lipases are found throughout all kingdoms of life. Due to their broad substrate specificity, these highly promising enzymes can be potentially used for biotechnological application in a wide range of industries (e.g. food, fragrance, cosmetics, textile, pharmaceutical, and detergent industry) [3]. They have been previously identified in a wide range of organisms, and several GDSL Ser esterases/lipases have been cloned and characterized. Many GDSL esterases/lipases have been found in bacteria, and advancement has been made toward uncovering their structures, functions, and physiologic roles [6-20]. The enzymes of GDSL esterases/lipases have been cloned and characterized, and at present, the crystal structures from *Streptomyces scabiesEscherichia coliPseudomonas fluorescensMycobacterium smegmatis*, and *Pseudomonas aeruginosa* are available [21-28]. Their mature enzymes display expansive hydrolytic activity with different types of substrates, including acyl-CoAs, a variety of esters, and amino acid derivatives.

All the structures of the GDSL esterase/lipase that have been described to date belong to the α/β hydrolase fold superfamily of proteins. The main difference in folding from classical α/β hydrolase fold is a distinct location of the residues involved in active site formation, which direct to a different analogous orientation of the catalytic triad with regard to the central parallel β-sheet [4,25]. Recently, the structure of the GDSL esterase/lipase proteins from several species of bacteria has been determined [21,23,25-28], but no structure from plants has been resolved yet.

The GDSL esterases/lipases have been also found in plant species and have become very attractive subjects because of their newly discovered properties and functions. Recently, in the plant kingdom, the novel family of the GDSL esterases/lipases is represented by more than 1100 members from the twelve different fully sequenced plant genomes. It was reported that GDSL family from *Arabidopsis thaliana* consists of 108 members [29], and *Vitis vinifera, Sorghum bicolourPopulus trichocarpa, and Physcomitrella patens* contain 96, 130, 126 and 57 members, respectively [30]. Search across multiple databases revealed 114 members from *Oryza sativa*, 53 members from *Zea mays*, 90 members from *Selaginella moellendorffii*, 88 members from *Medicago truncatula*, 102 members from *Chlamydomonas reinhardtii*, 59 members from *Ostreococcus tauri*, and 75 members from *Phaeodactylum tricornutum*[31,32]. Several plant GDSL esterases/lipases have been isolated, cloned, and characterized. Physiologically, the GDSL esterases/lipases that have been described so far are mainly involved in the regulation of plant development, morphogenesis, synthesis of secondary metabolites, and defence response [33-55].

Rice has become a model plant for genomic research of monocotyledonous species because of its small genomic size and economic importance, but our knowledge of the GDSL esterases/lipases gene family in rice is rather limited. Although there are more than 100 members of the GDSL esterase/lipase family in the rice genome, only a few GDSL esterases/lipases genes have been studied and the functions and properties of the majority of members remain unknown. Currently, only two rice GDSL esterases/lipases genes have been reported. *GDSL-containing enzyme rice 1* (*GER1*) and *wilted dwarf and lethal 1* (*WDL1*) were cloned from the rice genome, and their physiologic functions were suggested as regulatory in coleoptile elongation and plant growth in the seedling stage, respectively [56,57].

In the present study, 114 *OsGELP* genes were identified in rice. This is the first bioinformatics genome-wide survey of the *OsGELP* gene family with description of: the genomic distribution, gene structure of the *OsGELP* genes, phylogenetic analysis, as well as motif analysis, and structure modelling for the OsGELP proteins. More than 30 additional, clade-common and -specific peptide motifs outside the GDSL domain were uncovered, described, and their putative functionality based on the GDSL-lipase protein tertiary structure was proposed. Potentially important regions for substrate specificity and binding, as well as functional grouping according to the phylogenetic relations are discussed. The expression patterns of some representative genes analysed by quantitative real-time PCR in response to cytokinin hormone treatment matched with the digital expression results. The results of the microarray expression profiling under the different treatment conditions, and the phylogenetic relatedness of the genes were analyzed in order to predict their functions in rice.

Considering the fact that a very limited number of the *OsGELP* genes have been characterized to date, results reported in this study is the first step towards the understanding of the roles of the GDSL esterases/lipases in rice, which provide a solid foundation for function predictions of possible roles of the GDSL enzymes in rice. Our work introduces a fundamental framework for selection of appropriate candidate genes for the subsequent functional analysis of the *OsGELP* family members.

## Results

### Identification of the GDSL esterase/lipase family genes in rice

A total of 114 putative *OsGELP* genes were identified and designated as *OsGELP1* to *OsGELP114* based on their order and position in corresponding chromosomes 1–12 from top to bottom. Their gene name, locus ID, the accession numbers for coding sequences (CDSs), genomic DNA, cDNA, and predicted isoelectric points of all the 114 *OsGELP* genes are listed in Additional file 1. The open reading frame (ORF) sizes of the *OsGELP* genes vary from 570 bp (*OsGELP76*) to 1,362 bp (*OsGELP30*), with an average sequence length of 1,097 bp.

**Figure 1:**
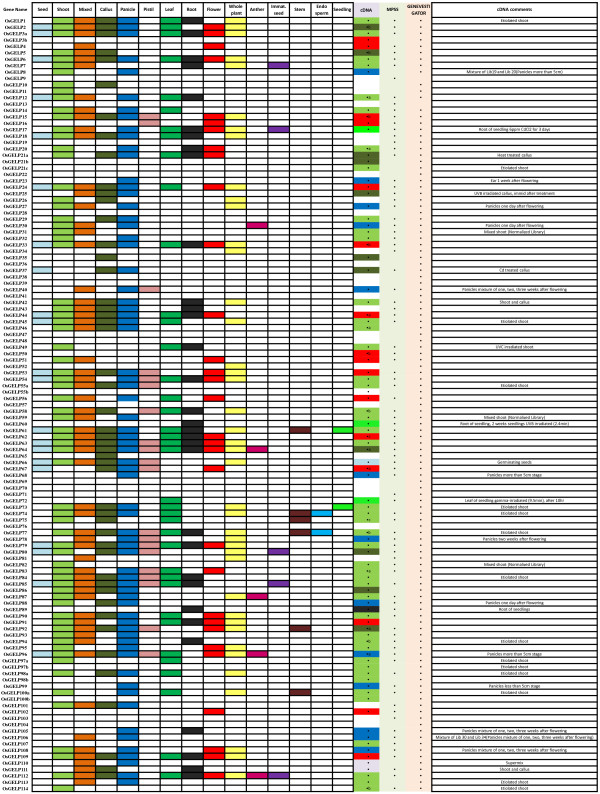
**The rice*OsGELP*gene expression anatomy viewer.** The expression patterns of 121 transcripts of 114 *OsGELP* genes in different rice tissues are shown. The evidence of gene expression for the genes is based on EST, FL-cDNA, MPSS, and Genevestigator data. A positive signal is indicated by a coloured box as follows: light blue for seed, light green for shoot, orange for mixed tissue, dirty green for callus, dark blue for panicle, light pink for pistil, green for leaf, black for root, red for flower, light yellow for whole plant, dark pink for anther, purple for immature seed, blue for endosperm, and lime for seedling. The white box indicates that no expression was observed. The colour in the cDNA column designates tissue library from where cDNA support was obtained. The black points display availability of expression data.

Most of the *OsGELP* genes are expressed in various organs. Ninety nine genes have one or more full-length cDNA (FL-cDNA) and/or expressed sequence tags (ESTs) ( Additional file 2). The expression of 13 other genes were confirmed by microarray data available at Genevestigator [58], and two (*OsGELP9* and *13*) genes had only MPSS data support (Figure 1). The number of mapped EST sequences for the *OsGELP* genes was quite variable, indicating marginal 1–3 (e.g., *OsGELP113452688289*, and *102*) to strong 100 to >200 (for *OsGELP3653637779*, and *85*) expression ( Additional file 2).

Up to 24.5% (28 of 114) of the *OsGELP* genes were predicted to be alternatively spliced by the Rice Genome Annotation Project (RGAP) database (release 6.1). The *OsGELP* genes are present in two to four alternatively spliced forms, giving rise to a total of 68 transcripts ( Additional file 1). This number is slightly higher than that predicted for rice genes overall [59]. The expression of 33 of the 68 transcripts was confirmed by FL-cDNA evidence (Figure 1Additional file 2). Several annotation errors were observed in the automated annotation of the rice genome, including intron/exon numbers/positions that were corrected according to the rice FL-cDNA sequences from the Knowledge-based Oryza Molecular Biological Encyclopedia database (KOME) [60]. For example, the annotation of two *OsGELP* (*OsGELP79* and *113*) genes were corrected. Their structure annotations were changed from 2 exon/1 intron into 3 exon/2 intron, and 4 exon/3 intron to 5 exon/4 intron patterns. Also, the predicted ORF sizes were modified according to the availability of FL-cDNA (AK066113 and AK063071), from 1,107 and 1,272 bp to 1,026 and 846 bp, respectively.

### Chromosomal distribution, gene structure and evolutionary expansion of the *OsGELP* genes

**Figure 2:**
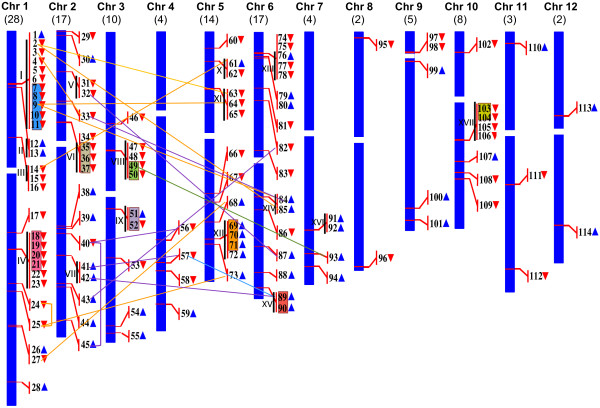
**Genomic distribution of the*OsGELP*genes in rice chromosomes.** The *OsGELP* genes are numbered 1–114. The white rectangles on the chromosomes (vertical bars) indicate the positions of the centromeres. Chromosome numbers are indicated at the top of each bar, and the number in parentheses corresponds to the number of the *OsGELP* genes present on that chromosome. The *OsGELP* genes present on duplicated chromosomal segments are connected by coloured lines (one colour per chromosome). The tandemly duplicated genes present in the same colour box. The roman numerals and vertical black solid lines show the number and specify groups of the closely linked genes identified as clusters. The blue and red triangles indicate the upward and downward directions of transcription, respectively.

Figure 2 is a diagrammatic representation of the chromosomal distribution and direction of transcription of the *OsGELP* genes in 12 rice chromosomes. As shown in Figure 2, the *OsGELP* genes are present in every chromosome, but their distribution is not homogeneous and uniform. For example, the highest number (24.6%) of the *OsGELP* was observed in chromosome 1, with a relatively high density of the *OsGELP* genes in some chromosomal regions (Figure 2). Also, a high number of genes are condensed on chromosomes 2, 6 (14.9% on each), and 5 (12.3%), whereas rice chromosomes 8 and 10 contain only two *OsGELP* gene loci each. Up to 46.5% *OsGELP* genes are located closely in chromosomes. These 54 *OsGELP* genes comprise 17 clusters, in which closely linked genes are adjacent or separated by 1 or not more than 4 unrelated genes (Figure 2Additional file 3). Interestingly, the genes that interrupt the *OsGELP* gene clusters encode mostly small-sized hypothetical or expressed proteins and large retrotransposon/transposon proteins. A total of seven clusters (I, II, IV, VI, IX, XI, and XIV), located in chromosomes 1, 2, 3, 5, and 6, contain a large number of transposable element (TE)-related genes inserted between 26 *OsGELP* genes. To understand the mechanisms underlying the evolution of the *OsGELP* gene family, both tandem and segmental duplication events were examined. A large number (19.3%) of the *OsGELP* genes were observed on duplicated chromosomal segments of rice ( Additional file 4). Furthermore, 25 of the 114 *OsGELP* genes that clustered in the same chromosomal regions (Figure 2) comprise eight groups of tandemly duplicated genes. Notably, we determined fifty three outparalogous genes (46.5%) that have undergone duplication after the split of eudiocts-monocots, but prior to sorghum and rice speciation, ( Additional file 5) using the phylogenetic study of Volokita et al. [30]. There is no consensus regarding the number of exons and introns in the GDSL gene structure. In most cases (49.1%), the *OsGELP* genes are interrupted by four introns and consist of five exons within their coding regions ( Additional file 6), which is consistent with the global analysis of the gene structure in the rice genome [61]. In other cases, the number of introns in the ORF varied from 1 to 6, and the *OsGELP39* gene was found intronless. The pattern with the highest number of exons was observed only in the *OsGELP109* gene (seven exons and six introns), whereas 4, 27, 16, and 9 genes held six/five, four/three, three/two, and two/one exon/intron patterns, respectively.

The chromosomal regions where the candidate genes reside vary in their size. Their genomic sequence lengths range from 1009 to 24,799 bp due to the large introns ( Additional file 7). The intron sizes of 45.6% of the *OsGELP* genes appear to exceed 1,000 bp. The *OsGELP21* and *OsGELP97* genes contain over 10-fold longer introns than the other genes in the family. The two huge introns from these genes, 12,861, and 11,743 bp, are consistent through all alternative splicing forms. Within these long introns, a total of 13 and 12 repetitive elements were detected. These elements are represented by different types of miniature inverted-repeat transposable elements, transposons, and retrotransposons. In general, the diverse repetitive sequences, from several superclasses with a variety of sizes, were discovered within introns, exons, and 5′ or 3′ untranslated regions (UTRs) of 71 *OsGELP* genes ( Additional file 8).

### Phylogenetic analysis and evolution of the *OsGELP* genes

To study the evolutionary relationship of the members of the *OsGELP* gene family, as well the phylogenetic relationship among the rice *OsGELP* genes and other plant GDSL genes, whose putative functions were elucidated recently, the unrooted phylogenetic trees based on the multiple sequence alignment of their protein sequences were constructed by the neighbour-joining (NJ) method and displayed using the Molecular Evolutionary Genetics Analysis (MEGA4) program.

For the rice *OsGELP* phylogenetic tree, a dataset of 96 protein sequences containing 13 conserved alignment regions were collected, including the special features of the GDSL esterase/lipase such as blocks I, II, III, and V. Other 18 *OsGELP* genes contain gap-rich regions. During evolution, they probably lost some common GDSL enzyme blocks, as well as other shared regions. For this reason, they were eliminated from further phylogenetic analyses ( Additional file 9).

**Figure 3:**
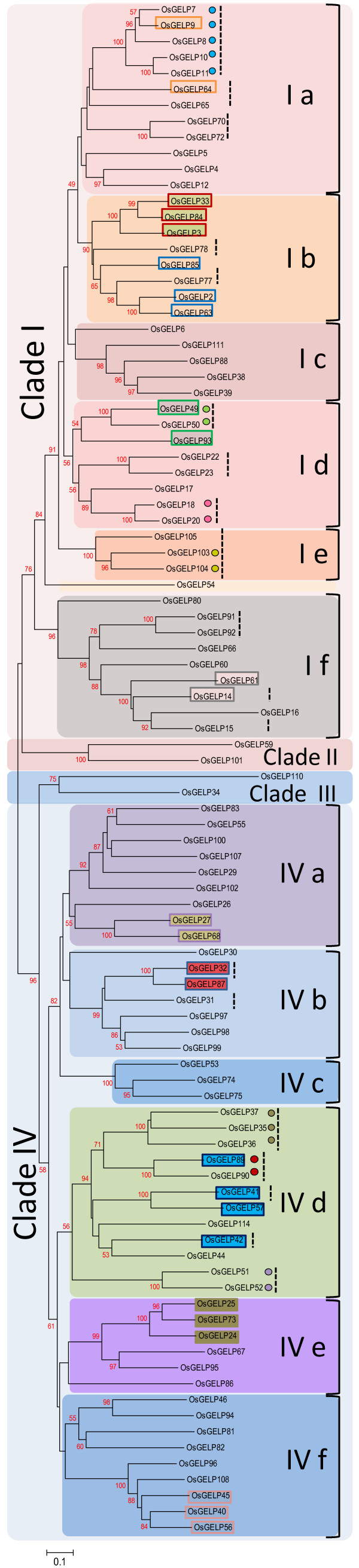
**The phylogenetic relationship of the*OsGELP*gene family.** The unrooted tree was constructed based on multiple sequence alignment of the rice OsGELP protein sequences using ClustalW program by NJ method with 1,000 bootstrap replicates. Subclades are numbered at the right part of the tree and marked with different alternating tones of a background to make subclade identification easier. *OsGELP* genes that are in the same coloured boxes are segmental duplicated genes. Coloured dots indicate genes in tandem duplication. Vertical dashed black lines point out genes from genomic clusters. The node numbers lower than 50 are not shown.

The rice *OsGELP* gene family was divided into four clades in the final unrooted phylogenetic tree construction (Figure 3). The result suggests that clades I and IV can be further subdivided into 12 subclades (6 per clade). The *OsGELP* genes, that grouped together in the subclades conformed their predictional arrangement of segmental and tandemic duplication events. *OsGELP*s from 15 of the 17 genomic clusters were verified to have close phylogenetic relationships through their high node numbers (Figure 3). Up to 62 *OsGELP* genes comprise 31 sister pairs. A total of 12 pairs belong to 10 gene clusters and 7 pairs are segmentally duplicated genes (Figure 3). Each subclade consists of one or more sister gene pairs. This suggests the major role of duplication events in the expansion of the *OsGELP* gene family in the rice genome.

**Figure 4:**
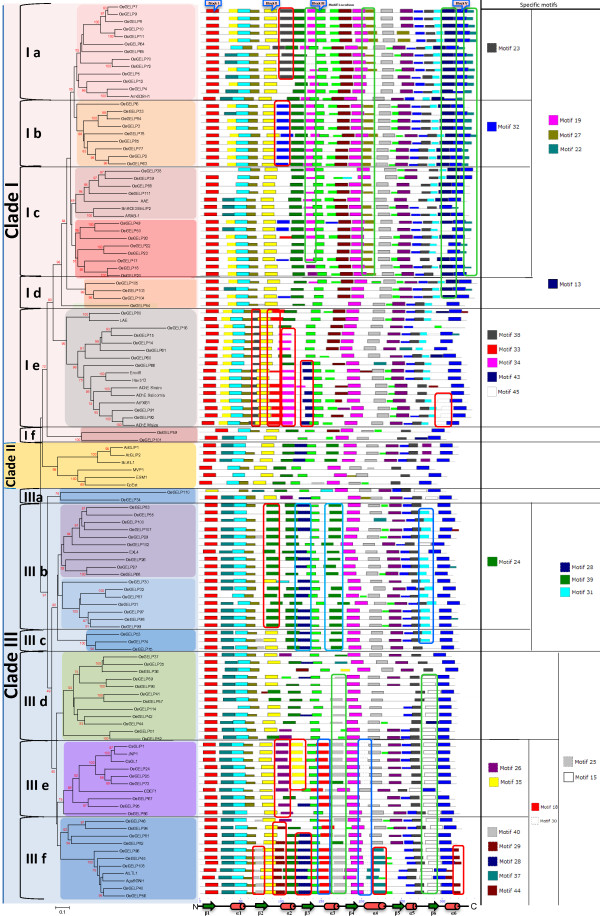
**An analytical view of the phylogenetic relationship among the rice OsGELP and plant homologues of known function.** Protein NJ tree: The unrooted tree, constructed using ClustalW, summarizes the evolutionary relationship among 120 members of the GDSL esterase/lipase plant family. The NJ tree was constructed using the alignment of only the highly conserved amino acid sequence regions. The tree shows 13 major phylogenetic groups. Left column identifies subclades and is marked with different alternating tones of background to make subclade identification easier. The numbers beside the branches represent bootstrap values based on 1,000 replications. The node numbers lower than 50 are not shown. Protein motif structure and location: the OsGELP and plant GDSL esterase/lipase proteins are in the order of their appearance in the phylogenetic tree. Each coloured box represents particular motif. Their consensus sequence, length (amino acids), number of the GDSL esterase/lipase proteins containing the motif, and E-value are given in Additional file 11. The GDSL motif blocks I, II, III, and V are indicated in pink boxes above the motif distribution pattern. The length of proteins (amino acids) can be estimated using the scale at the bottom. Motifs enclosed in red, blue, or green frames are highlighted motifs that exclusively appear in proteins from one, two, or three subclades, respectively. The number of highlighted motifs specific for one or several subclades is given at the right. The secondary element assignment, below the motif distribution scheme, corresponds to the general structure of the OsGELPs.

Given that orthologs frequently hold an identical function [30,62], our second unrooted NJ phylogenetic tree combined 96 rice *OsGELP* genes and 24 plant GDSL orthologs or homologs whose putative functions were annotated recently (Figure 4Additional file 10). According to the phylogenetic analysis, the *OsGELP* genes and their close plant orthologs or homologs were divided into three major subfamilies represented by clades I, II, and III. In addition, clades I and III each were separated into six subclades (Figure 4). Among the plant GDSL esterases/lipases whose functions have been determined, 5 genes (*ARAB-1AtFXG1*, maize *AChECDEF1*, and *AtLTL1*) were found as orthologs of the 15 *OsGELP* genes ( Additional file 10). Orthologs, as well as the close homologous proteins, share more than 40% similarity and assemble together in the same subclades of the phylogenetic tree. All 12 subclades of the *OsGELP* tree order remained conserved in the newly generated conjoint plant GDSL esterase/lipase gene family tree constructed from a total of 120 members (Figure 4). Locations of the plant GDSL genes that were chosen for our study coincided with the previously reported tree topology of the GDSL esterase/lipase gene family in land plants (Embryophyta) [30].

Of the four clades of the original rice *OsGELP* phylogenetic tree, a new clade of the plant GDSL genes appeared. The emerging clade (II) is well supported by the bootstrap value (98%) and consists of six members of the GDSL esterase/lipase genes from *A. thalianaBrassica rapa*, and *Carica papaya*, which have been shown to be correlated with different kinds of biotic stress responses, except one *CpEst* gene (Figure 4) [33-38,63]. The specific nature of clade II in the tree can be explained by the association of the clade members with the myrosinase–glucosinolate system. This system is almost exclusive to the order Capparales, which includes the Brassicaceae plants [34]. This fact can account for separation of the group of genes in clade II from the other clades in the phylogenetic tree, and every member shows relatively low similarity (below 35%) to the *OsGELP* genes (Figure 4Additional file 10).

### Relationship between protein motifs and phylogenetic classification

A total of 45 motifs with statistical significance (E-value) from 1.3e-966 to 9.1e-002 were found among the OsGELPs and the known plant GDSL esterase/lipase proteins ( Additional file 11). Motifs 3, 5, 6, and 2 represent GDSL esterase/lipase conserved blocks I, II, III, and V, respectively (Figure 4, Additional file 11). As expected, the presence of the common GDSL domain proteins, represented by the four blocks, affirms its major functional role. Other well-conserved motifs outside the GDSL domain were also detected. Significantly, 12 conserved motifs (1–12 with E-values around e-100) with more than 10 but less than 15 amino acids in length are present in almost all proteins ( Additional file 11). The other 33 motifs were found to be specific to the different subclades of the GDSL esterase/lipase phylogenetic tree. We found that the GDSL proteins that cluster in clade I in the phylogenetic tree share a similar motif pattern (motifs 14, 16, 20, and 21), whereas there were no specific motifs for clade III. At the same time, the subclades of clade III demonstrate high diversity in specific motifs (Figure 4). Most of the OsGELP proteins that clustered together with homologs and/or orthologs in the same subclade share more than one additional conserved motifs outside the GDSL domain. Motifs 13, 19, 22, and 27 are specific to subclades Ia, Ib, Ic, and Id, whereas motifs 33, 34, 38, 43, and 45 exclusively appear in subclade Ie (Figure 4). Subclades IIIa, IIIb, and IIId, IIIe, IIIf contain specific motifs 28, 31, 39, and 15, 25, respectively (Figure 4, Additional file 11). Subclades Ia and Ib exclusively contain motifs 23 and 32, respectively. Motif 24 is specific to subclade IIIb. Subclade IIIe appears to have distinct motifs 26 and 35. Finally, five particular motifs (28, 29, 37, 40, and 44) belong to subclade IIIf (Figure 4).

The newly found additional, subclade-specific motifs were considered as novel, because there were no any statistically significant sequence similarities of our motifs with known motifs or possible function assignments within the Prosite and UniProtKB/Swiss-Prot databases [64,65].

### Distribution of the conserved motifs and their locations on the three-dimensional structure

We consider the possibility that the consensus regions outside of the motifs encoding GDSL esterase/lipase conserved blocks I, II, III, and V may contain functionally important motifs involved in substrate specificity, protein structure ordering and arrangement, protein–protein interaction, etc. Such “supplemental” functional motifs often remain conserved among members of a subgroup in large families in plants [66,67]. Thus, the proteins within the subgroups that share these motifs likely display similar functions. To find the three-dimensional orientation of these additional motifs, in order to support our functional prediction, the structure prediction were conducted on the OsGELP proteins using the Protein Homology/analogY Recognition Engine (PHYRE) server [68].

**Figure 5:**
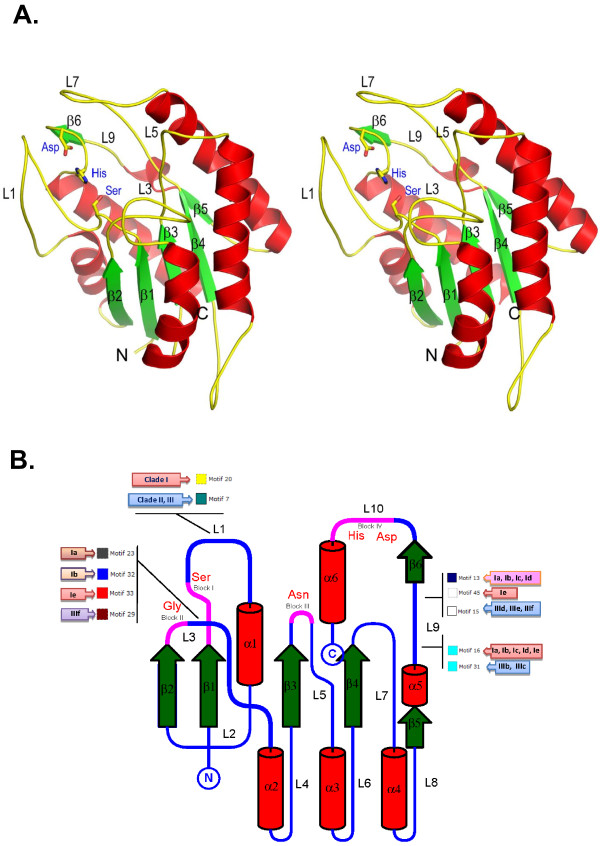
**Schematic diagrams of the structure prediction for the rice OsGELP esterase/lipase proteins.A.** The stereoview of the ribbon diagram for general structure prediction model of the OsGELP proteins is given. The six-stranded β-sheet is labelled. The catalytic triad Ser, Asp, and His are shown as sticks. **B.** Common schematic view of the OsGELP protein secondary structure. The folds showing six parallel β-strands are labelled β1–β6 and helices are labelled α1–α6. The loop regions are labelled L1–L10. The location of the GDSL consensus blocks is coloured magenta and catalytic residues are shown. Highly variable motif composition loops (L1, L3, and L9) are pointed out. The phylogenetic subclade in Figure 4, which contains specific motif(s) within the mentioned loops, is enclosed in shaded coloured boxes next to the motif’ numbers.

The structural homology detection showed four of the most closely homologous structures of the bacterial GDSL motif proteins. The lipase/acylhydrolase from *Enterococcus faecalis* [Protein Data Bank (PDB) code 1yzf] showed 10%–15% similarity, esterase from *Streptomyces scabies* (PDB code 1esc) demonstrated 10%–14% similarity, and thioesterase I from *E. coli* (PDB code 1ivn) showed 15–18% similarity. Finally, the general prediction model of the OsGELP proteins was built using the X-ray structure of the aryl esterase from *M. smegmatis* (PDB code 2q0q), which showed the highest similarity from 17% to 19% (Figure 5A).

The predicted basic structural model consists of six α-helices and a central β-sheet core containing six parallel β-strands (Figure 5). The active Ser residue is located in the loop region (L1) right after the first β-strand; meanwhile, in the bacterial structural model, Ser appears in a short helical segment following the first β-strand. The aspartic acid and His residues, which together with Ser form the catalytic triad, seems to hold the same location in plants and bacteria, and reside in the turn structure preceding the C-terminal α-helix (Figure 5B). Blocks II and III with their representative Gly and Asn residues, which act as proton donors to the oxyanion hole, are located in the unstructured regions following the second β-sheet and right after the third β-sheet, respectively, and designated in Figure 5B as L3 and L5.

Moreover, many predicted putative motifs within the unstructured loop regions were observed to be specific to the members of phylogenetic clades I or III and/or the subclades of these clades (Figure 5B). Three loops (L1, L3, and L9) can be specified as the most divergent in terms of motifs for the different OsGELP phylogenetic groups that deviate in biological functions. These particular loops possibly play a role in differentiation of substrate-binding specificity for the different subclades and thus bring their broad functional divergence.

## Discussion

For plants, during the course of their evolution, gene families generally underwent either tandem and/or large-scale segmental duplication to maintain a high number of family members [69-71]. The phylogenetic tree (Figure 3) demonstrates that the genes from 7 gene clusters are sister genes, with high degrees of phylogenetic relatedness. Only 17 genes from gene clusters I, IV, VI, VIII, and XII probably emerged as a result of local duplication, as it was previously shown by the phylogenetic analysis by Volokita et al. [30]. The phylogenetic study of plant GDSL esterases/lipases from bryophytes, gymnosperms, monocots, and eudicots suggested that duplication of more than 40% of rice GDSL genes predated the sorghum-oryza split [30]. If this number is combined with the number of other instances of genes’ duplication events, such as segmental or tandem duplication, the high number (71%) of the *OsGELP* genes potentially arose from such mode of evolutionary novelty. Taken together, the data suggest that duplications in general played a major role in the multiplication of the *OsGELP* genes, in the course of evolution. These conclusions are in line with a previous examination of the evolutionary mechanisms of the GDSL esterase/lipase gene family in land plants [30]. The fact that many *OsGELP* gene clusters are interrupted by a number of TE-related gene insertions implies that duplication events of the GDSL esterase/lipase protein family genes were followed by insertion of the TEs throughout the course of their evolution. The large number (62%) of the *OsGELP* genes with TEs can be also regarded as supporting evidence in favour of the theory that subsequent and important events for the expansion in size of the *OsGELP* gene family in the rice genome after duplication could be the amplification of the repetitive elements ( Additional file 8). This observation is consistent with the previous conclusion that one of the forces for amplification of the rice genome is the addition of TEs [72].

Several forms of gene regulation, positive and negative, that involve plant introns were found [73]. Considering that the intron evolution in the rice genome is largely dominated by intron loss [74,75], the large introns within the *OsGELP* genes that were left in the course of natural selection are likely due to their possible functionality. Recent studies have shown that some introns can function as alternative promoters or enhancer elements, and some introns promote mRNA accumulation through diverse processes called intron-mediated enhancement [73]. In addition, in contrast to exon evolution, introns appear to be under a lower selection pressure; thus, they could frequently vary in size and sequence, and slowly diverge if their position in the genes that facilitate the evolution of new proteins through exon shuffling and alternative splicing increased the coding capacity of a genome [73,76,77]. Although the *OsGELP* genes with long introns contain repetitive elements, the majority of them (47 of 52) are expressed. For example, aforementioned *OsGELP21* and *OsGELP97* genes are expressed in various rice organs in three and two alternative splicing forms as supported by cDNA evidence (Figure 1Additional file 2). Stress conditions are one of the effectors of the alternative splicing of pre-mRNAs because stress regulation might enable plants to quickly regulate the splicing and gene expression of many unrelated genes [61]. Many alternatively spliced transcripts that were expressed under stress conditions were found among long intron genes (Figure 1). For instance, the *OsGELP21* gene that encodes three alternative spliced forms in the first and third forms is expressed in the shoots and calluses under the etiolation and heat treatments. These results suggest that subsequent studies should continue to investigate the advanced functions and transcriptome complexity of the *OsGELP* gene family.

**Figure 6:**
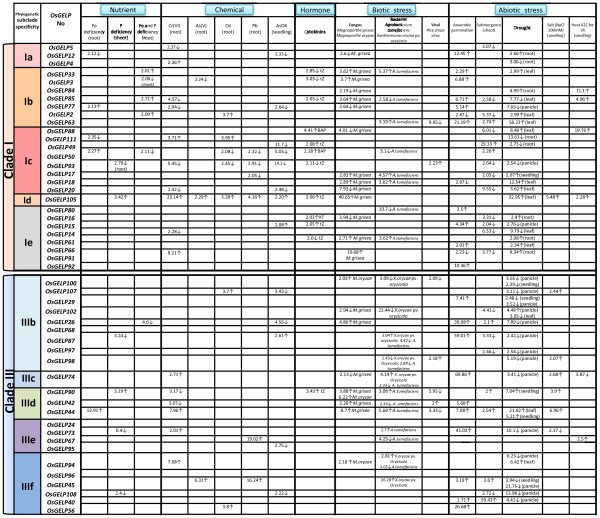
**Expression pattern of the*OsGELP*genes with predicted functions in response to different treatment conditions.** The microarray data-based expression profiles under various conditions are presented using the meta-profile analysis tool at Genevestigator for 50 *OsGELP* genes. The transcript levels are depicted by numbers indicating relative fold values. The *OsGELP* genes are in the order of their appearance in the phylogenetic tree. The number of clades and subclades are presented in the left side of the diagram. The subclades are highlighted in the same alternating tones as they were shadowed in the phylogenetic tree in Figure 4.

In accordance with the phylogenetic analysis, 24 plant GDSL esterases/lipases genes, whose functions were elucidated recently, fell into two putative groups that differ in their generic biological processes: clades I and III. In general, according to the experimental findings [33-57], the *OsGELP* gene orthologs and paralogs of known functions from clade I can be potentially involved in the secondary metabolism pathways, plant development and morphogenesis, whereas the orthologs from clade III seem to play a role in plant defence and reproduction ( Additional file 10). Furthermore, to show possible function divergence of *GELP* genes in rice, the microarray expression data of clade I and clade III were searched in terms of their responses to different treatment conditions by querying the Genevestigator microarray database [58]. With the 2-fold expression difference cutoff, the expression profiles of 50 *OsGELP* genes that share 28 to 80% similarity, to the 24 GDSL esterases/lipases genes of known functions are summarized in Figure 6 ( Additional file 10). As shown in Figure 6, such factors as nutrient deficiency, chemical and hormonal treatments, biotic and abiotic stresses can modulate the expression of these 50 genes. The most notable expressional difference between clade I and III seems to be in response to the cytokinins *trans*-zeatin (tZ), 6-benzylaminopurine (6-BAP), or kinetin (KT)] treatment (Figure 6). Cytokinins are a class of plant hormones associated with regulations of plant growth and development, chloroplast biogenesis, bud and root differentiation, shoot meristem initiation and growth, stress tolerance, and organ senescence [78]. Expression profiles of genes from clade III do not show significant change in their expression fold in the presence of the cytokinin. At the same time, many members of clade I show differential expression under KT, tZ, BAP hormones treatment (Figure 6), implying the possible role of the genes from clade I in plant growth and development.

To validate the results of the microarray data obtained from Genevestigator database, changes in the expression level of 17 representative *OsGELP* mRNAs from clades I and III, under cytokinins (tZ, KT, or BAP) treatments in rice seedling were examined by quantitative real-time RT-PCR. The treatment conditions were repeated according to the description of the experiments in the Genevestigator database. The expression patterns that were obtained via RT-qPCR for 8 and 2 selected genes which were treated with tZ and BAP, respectively, followed the same tendency and confirmed the microarray data ( Additional file 12). Results of the digital expression analysis for the *OsGELP2, 17, 12, 61, 44, 77, 90, 100*, and *OsGELP9*2 genes were not coherent with the qPCR analysis, and did not show up-regulation during the tZ and KT treatments, respectively. Although, most of the genes from both clades showed up-regulation of their expression, only 3 genes (*OsGELP15, 50, 88*) from clade I were significantly up-regulated (>2-fold) after treatment with tZ or BAP hormones for 30 min or 3 h, respectively. At the same time, none of the genes from clade III demonstrated significant fold change under the cytokinins treatment ( Additional file 12), therefore suggesting functional differentiation of the two examined clades. Further experiments are needed to confirm the microarray validity in order to explore functional divergence of the *OsGELP* family.

Following the assumption that functional information of unknown GDSL esterases/lipases can be deduced from the orthologs of known functions [30], we attempted to extrapolate the functional characteristics of described plant GDSL onto the *OsGELP* rice genes*.* Using the functional descriptions of the potential orthologs and homologs, based on the phylogenetic grouping, the possible putative functions for a number of the *OsGELP* genes were predicted, and discussed further.

The rice GDSL esterase/lipase family members *OsGELP45*, and *12* from subclade Ia share high similarity with *AmGDSH1* (*Alopecurus myosuroides* hydrolase) that demonstrates acetylajamaline hydrolase activity and it is involved in alkaloid metabolism [47]. Subclade Ib (*OsGELP23637784*, and *85*) is expected to be involved in plant development and morphogenesis at the seedling stage according to the function of their close homologue *GER1* (*OsGELP33*) [56]. These genes are not only expressed in many rice organs and development stages, as well as share analogous gene structure and special protein motif 32, but also change their expression dramatically under stress conditions during early plant development (Figures. 1,4, and 6). Two genes from clade Ib have received attention in recent literature. The *OsGELP63* gene is induced by both red and far-red light and by jasmonic acid, and acts in response to drought and cold stresses [79]. The study of *OsGELP33* (*GER1*) has demonstrated the role of this gene in the rice plant development at the seedling and coleoptile elongation stages [56]. *OsGELP33*, together with its sister genes *OsGELP84* and *OsGELP3*, arose from the segmental duplication event (Figure 2). Their close phylogenetic relationship is confirmed by the high node number and the high protein similarity score (Figure 4Additional file 10). Therefore, the functions of these genes might be similar to that of the *OsGELP33* (*GER1*). Subclade Ie, mentioned previously, is a good example of the group of genes with possibly related functions. The genes in subclade Ie appear to belong to the cell wall-associated proteins with carbohydrate substrate specificity (Figure 4Additional file 10). Together with the cell wall-associated GDSL esterase/lipase orthologs (e.g., *AtFXG1LAEEnod8*, maize *AChE*, and *Hevb13*) [46,50,51,53,54,80], rice *OsGELP1415166061668091*, and *92* genes form a distinctive group in clade I (Figure 4). The α-fucosidase 1 (*AtFXG1*) from *A. thaliana*, lanatoside 15′-*O*-acetylesterase (*LAE*) from *Digitalis lanata* Ehrh. Woolly, and their homologue Early nodulin protein (*Enod8*) from *Medicago sativa* are reportedly active on oligo- or polysaccharide substrates [46,50,51]. *LAE* acts as deacetylators on cardenolide glycosides (cardenolides that contain structural groups derived from sugars) [51]. *AtFXG1* modifies xyloglucan oligosaccharides through the hydrolysis of t-fucosyl residues [50]. The representatives of the acetylcholinesterase (*AChE*) gene family have been characterized and cloned recently in several plants, including *Z. mays* L., *Macroptilium atropurpureum*, and *Salicornia europaea* L. [52-55]. Although the definite physiologic role of the *AChE* gene family has not been elucidated yet, *AChEs* are suggested to play a role in the gravity response of plants. According to the motif analysis, this group of the cell wall-associated proteins shares several special motifs in the subclade Ie, such as motifs 38, 33, 34, 43, and 45 (Figure 4). A total of 29 rice *OsGELP* genes from clades I and III putatively can be important to the plant defence response against biotic infections, as evident from their microarray expression data (Figure 6) and relatively high similarity to the number of defensive GDSL esterases/lipases (e.g., *CaGLIP1CaGL1AtLTL1GLIP1GLIP2Br-SIL1ESM1*, and *MVP1*) ( Additional file 10) [33-41]. As potentially appealing subjects for future analyses of the *OsGELP* gene family, subclade IIIf can be specifically studied. It not only contains five different exclusive motifs (28, 29, 37, 40, and 44), but also some of its members show expression in all rice organs and share similar gene structure patterns within a particular subclade (Figures. 1 and 4Additional file 6).

Based on the protein sequence analysis, a diversity of the consensus regions outside of motifs that encoded the GDSL esterase/lipase conserved blocks I, II, III, and V was found. These consensus motifs are specific to different phylogenetic clades and/or subclades from conjoint tree that differed in biological functions (Figure 4). The GDSL esterases/lipases are active on a wide range of substrates. This multienzymatic activity can be explained by the flexible substrate-binding pocket in the active site, which facilitates the binding of different substrates [3]. Considering that many motifs can be functionally important and play a role in enzyme specificity and biochemical activity, the long loop regions extending from the protein core in the plant GDSL esterases/lipases might be involved in the diversification of molecular multifunctionality, as this was found in bacterial species [25,27]. For example, aryl esterase from *M. smegmatis* and thioesterase I from *E. coli* share a common structural fold, but differs in the additional insertions—unstructured loop regions in the aryl esterase proteins. It was suggested that such insertions might determine the type of enzymatic mechanism, contribute to the oligomerization, and greatly restrict the shape of the enzyme active site [27]. Many of the predicted motifs within the loop regions were found to be specific to the members of particular phylogenetic subclades that unite the GDSL enzymes with similar biological functions (Figures. 4 and 5). Based on these findings, we would like to specifically highlight L1, L3, and L9. The peptide regions of these loops are specific to different subclades from clades I and III. Based on the functional prediction, these subclades represent the proteins with different molecular functions and reaction types. As shown in the 3D protein structure prediction model (Figure 5A), loops L1, L3, and L9 are hypothetically oriented around the enzyme active site and function in the flexibility of the substrate-binding pocket. Therefore, these loops should be studied further to determine their role in molecular functional diversification of the plant GDSL enzymes. Experiments using reverse genetics would be required to establish contribution of these motifs. The close homologs or orthologs from plant species with known putative functions, which cluster together with the OsGELP proteins in the same subclades, share one or more additional conserved motifs (Figure 4). Although the functions of these specific motifs outside the GDSL esterase/lipase domain are still unknown, the presence of the conserved motifs certainly reflects the functional similarities among the OsGELP proteins that share these common motifs with other plant homologue proteins of known function.

The rice GDSL esterase/lipase family is notably one of the 11 largest families in the rice genome, with more than 100 members [81]. In other fully sequenced plant genomes, the GDSL esterase/lipase family also consist of high number of family members [29,30]. The remarkably high number of genes in the GDSL family in different plants can be explained by differences in enzyme function and activity on a wide range of substrates, as it was shown by Volokita et al. [30]. This claim is supported by the existing data collected by investigations of the GDSL esterases/lipases, which have already undergone functional analysis, cloned, and characterized in different plant species, and whose physiologic role, properties, and functions have been elucidated ( Additional file 10). The multifunctionality of the OsGELP family in rice, as well as in other land plant species, their diverse roles in different aspects of plant growth and development can be explained by the complexity and diversity of the genes at the structural level. The large number of genes that comprise the GDSL esterase/lipase family in land plant species, with many distinct groups and subgroups arising in the course of evolution, further explains functional divergence. Hypothetically, plant GDSL esterase/lipase proteins are the evolutionary product of recombination of several proteins, and contain various domains/motifs with putative functions. Such an assumption provides a clue to further study the diverse functionality of this enzyme family. Motif search analysis, presented here, offers further evidence for such supposition. Our manuscript introduces, for the first time, a concrete rationale for further experimentation with the rice *OsGELP* family members, and presents unique opportunities, and articulates coherent basis for functional studies. Further analyses of the gene functions using RNAi and overexpression are currently under way to elucidate the mechanisms further.

## Conclusions

The present bioinformatics analysis accommodates new insights into the genomic and proteomic diversity of the rice GDSL esterase/lipase gene family. The phylogenetic analysis divides the *OsGELP* gene family into the distinct groups that share similar protein motif structure. We found 41 additional motifs that represent the core secondary structure elements or appear specifically in different phylogenetic subclades. Members within the specified subclades can have common evolutionary origins, and obtain common unambiguous motifs that probably reflect their related molecular functions. Thus, our study support required basis, and should stimulate future full-fledged functional studies of these particular motifs, as understanding the structure-function relationship of the members of the *OsGELP* gene family is necessary.

Recently, only few rice *OsGELP* genes have been studied in order to determine their function. Here, we provide a rationally reasoned, well defined platform for more detailed functional, in-depth studies of the *OsGELP* genes based on combination of the phylogenetic, motif, and protein dimensional structure analyses. The findings presented in our manuscript can be utilized for selection of candidate genes for functional validation studies. It is of broad interest to the biological research community with wide and important practical applications in biotechnology and food science. The researchers from different domains, with different goals will find our analyses crucial for the initiation of their investigations.

## Methods

### Identification of genes coding the GDSL esterase/lipase in genome sequences of rice subsp. *japonica* cv. Nipponbare

A total of 132 genes were identified as possible candidates of the GDSL esterase/lipase proteins using primary bioinformatics analysis. First, the genes previously annotated as GDSL esterase/lipase were collected from several public online databases, such as MSU RGAP (release 6.1), Rice Protein Database in GRAMENE, and GenBank from the National Centre for Biotechnology Information [31,32,82]. Then, multiple BLAST algorithm analysis of the primary candidates, using the typical GDSL esterase/lipase protein sequence as our query, was done. The OsGELP candidates were tested against the Hidden Markov Model (HMM) profile (build 2.3.2) of GDSL domain, numbered PF00657 in the Pfam HMM library in the MyHits protein domains database [83]. All sequences with an E-value below 0.1, gathering cut-off above −69.0, and length above 100 amino acids were selected for further analyses. Subsequently, five genes that possessed repetitive sequences and were defined as retrotransposon genes, such as LOC_Os01g12340, LOC_Os01g32630, LOC_ Os06g24420, LOC_Os10g09130, and LOC_Os11g19690, were excluded from our analyses. We also eliminated several putative *OsGELP* genes that were annotated as esterase, anther-specific proline-rich protein APG precursor, alpha-l-fucosidase 3 precursor, hypothetical protein, expressed protein, and carboxylic ester hydrolase, and had GDSL motif with Pham E-value less than 0.1 ( Additional file 13).

The nomenclature of the *OsGELP* genes is based on the arrangement of positions on rice chromosomes 1–12. In the present study, the LOC prefix from all RGAP locus IDs that represent the GDSL esterase/lipase genes were removed for convenience. Information regarding ORF length, amino acid number, molecular weight, and isoelectric point of protein was downloaded from RGAP. The full-length cDNAs of all predicted genes were searched in the KOME [84]. Genomic sequences that were misannotated compared with available FL-cDNA sequences were corrected manually for the following analysis.

### Distribution of the *OsGELP* genes on rice chromosomes and duplication events

The chromosomal distribution of the predicted *OsGELP* genes members was retrieved from the RGAP database. Information regarding their physical positions was derived from the RGAP database according to the location of the rice chromosome pseudomolecules [32]. To identify the closely linked *OsGELP* genes, defined as gene cluster sets, in the rice chromosomes, the RGAP Rice Genome Browser was explored. Segmental duplication analysis was done with the RGAP rice segmental duplication database with the maximum length distance permitted between collinear gene pairs set to be 500 kb. The information on tandemly duplicated *OsGELP* genes, paralogs, and orthologs was obtained from the Rice Protein Database in Gramene [31], the Kyoto Encyclopedia of Genes and Genomes (KEGG) Database [85], the GreenPhyl Orthologs Search Tool (GOST) [86], and the Orthologous Groups Search page on RGAP. Outparalogs were determined from phylogenetic analyses of GDSL esterase-lipases from 7 plant species by Volokita et al. [30]. Proteins designated as homologous to 24 plant GDSL esterase/lipase genes, whose putative functions were annotated recently, share 30%–80% similarity.

### Exon/intron structure and sequence analysis

The exon/intron structures of the *OsGELP* genes were retrieved from the RGAP [32] and Gramene/Ensembl Genome Annotation for Rice [31]. For genes whose cDNA sequences were available, their structure was checked manually, aligning genomic and cDNA sequences. The diagram of the exon/intron structures and information on intron distribution pattern were obtained using the online Gene Structure Display Server [87]. The alternative splicing of the *OsGELP* genes was validated manually by alignment of rice FL-cDNA with genomic sequences or using RGAP Rice Genome Browser. The repetitive sequences were screened using RepeatMasker database [88].

### Multiple sequence alignment, and phylogenetic analysis

The *OsGELP* genes nucleotide cDNA and CDS sequences were translated into protein sequences. The protein sequences were aligned using multiple sequence alignment via the ClustalW method and were then manually corrected and implemented in the MEGA4 software (version 4.0) [89]. A total of 18 *OsGELP* genes were excluded from the final alignment because of the absence of some conserved GDSL blocks and poorly matched alignable regions with gaps. The culled protein set consisting of 96 *OsGELP* genes was used to construct trees. Second unrooted NJ phylogenetic tree combined 96 *OsGELP* genes and 24 plant GDSL orthologs or homologs whose putative functions were annotated recently following by procedure described by Volokita et al. [30].

A multiple-step strategy was used to construct the phylogenetic trees. Very large protein families commonly contain various domains and repeats that make them extremely difficult to analyze. The special feature of the GDSL esterases/lipases is the presence of the four strictly conserved residues Ser-Gly-Asn-His in conserved blocks I, II, III, and V. Consequently, our first consideration was to construct the phylogenetic tree based on the four blocks of the GDSL enzyme. Surprisingly, the node numbers were very low, and any kind of phylogenetic tree analysis would not help. The multiple alignments showed diversity of the strictly conserved areas that were consistent throughout the protein sequences of all GDSL candidates, along with the less conserved regions with gaps. To analyse those well-conserved regions, a motif identification search was conducted together with the protein structural prediction analysis. First, using Multiple Em for Motif Elicitation (MEME) program, the additional putative conserved motifs from a total of 120 plant GDSL esterase/lipase proteins (96 rice OsGELP proteins and 24 plant GDSL esterases/lipases whose putative functions were elucidated recently) were identified [90]. Second, after the structural topology of the OsGELP was predicted, the multiple sequence alignment, motif search, and protein structure analysis were analytically combined. Thirteen aligned regions (including GDSL esterase/lipase blocks I, II, III, and V) were found to be consistent throughout all 120 proteins and, in most cases, they encode the core secondary structure elements such as α-helices and/or β-sheets. Assuming that these core structure regions are mainly ancient, less mutated, and, probably, in the course of evolution, were under the lowest selections pressure, the phylogenetic study was performed based on these well-conserved regions. As a result, the trees were based on 13 conserved alignment blocks, which are represented by 23 putative conserved motifs (motifs 1–7, 10–12, 17, 20, 22, 24, 27, 30, 36–38, 40, 42, and 44) that were identified through motif search analysis ( Additional file 14). The phylogenetic trees that were built based on that strict alignment blocks showed the highest node numbers compared with the other trees that were based on full-length or four GDSL block alignments. In parts of the sequences that were out of those well-conserved alignment regions, including the N- and C-terminus, rich gap parts were manually removed from the alignment and phylogenetic analysis of all 120 GDSL protein sequences. Finally, two unrooted phylogenetic trees were constructed using the NJ method and were displayed using the MEGA4 program. The bootstrap values of 1,000 replicates were placed at the nodes, and the scale bar corresponded to 0.1 estimated nucleic acid substitutions per site. The topologies of the eventual unrooted NJ trees were maintained in trees that were built using the distance or parsimony methods.

### Determination of conserved motifs, and structure modelling

To identify the additional putative conserved motifs in the rice *OsGELP* gene family and in 24 plant GDSL esterases/lipases, whose putative functions were recently elucidated, the MEME motif search tool was used [91]. During our motif distribution search, different sets of parameters for width, number, and occurrences were tried for a single motif. Our final motif search was based on the following criteria: number of repetitions, zero or one per sequence; maximum number of motifs, 45; optimum motif width, ≥6 and ≤15. The N and C-termini were removed from all protein sequences in the final motif search after we confirmed no additional motifs were present in those parts. To determine which of our motifs can be considered novel, all regular expressions of found motifs were compared against the Prosite database patterns [64]. Functional annotation search was completed with UniProtKB/Swiss-Prot and Prosite databases [64,65].

To gather information about the secondary and tertiary structure of the OsGELP proteins, 3D models were constructed using the automatic protein structure homology modelling server using the PHYRE software [68]. Each submitted OsGELP sequence was scanned against the non-redundant sequence database structural classification of proteins and the PDB database. Aligned structures were displayed and analyzed within the PyMOL Molecular Graphics System [92]. Topology map was created using the *TopDraw* program [93].

### Expression analysis of the *OsGELP* genes

The evidence of expression of the rice *OsGELP* genes was obtained by several types of transcript data, such as FL-cDNA, EST, and/or MPSS from Expression Evidence Search page at RGAP [32], and the microarray data were available at the Genevestigator site [58]. The locus name of the GDSL esterase/lipase genes was used to query the MPSS database containing the signature information of the genes [94].

### Hormone treatment and quantitative real-time RT-PCR analysis

To confirm the differential expression of representative *OsGELP* genes under the hormone treatment identified by microarray data analysis, the tissue samples of seedling, from the rice (*O. sativa* L. cv Tainung 67, a japonica variety) were collected. The seeds that were sterilized with 70% ethanol for 15 min and then with 2% (w/v) sodium hypochlorite for 15 min, soaked in distilled water at 30°C for 1 day, and germinated seeds were grown for 7 days or 2 weeks with a photoperiod of 12 h light (30°C)/12 h dark (28°C). For hormone treatment with tZ, the whole roots were cut at the lamina joint in water from the 2-week-old seedlings and immediately dipped in distilled water containing either 5 μM *trans*-zeatin in dimethylsulfoxide [DMSO; 0.1% (v/v)] or an equal volume of DMSO as a control. Each excised organ was incubated at 30°C for 30 min, as it was described previously [95]. For kinetin responsive study, rice seeds were germinated and grown hydroponically in nutrient solution [96]. Seedling samples grown till the 3-leaves stage (two-week-old seedlings) and then treated with 100 μM kinetin for 60 min. For cytokinin treatment with benzyl aminopurine (BAP), rice seedlings that were grown hydroponically for 7 days, were transferred to a solution containing 50 μM benzyl aminopurine for 3 h. Seedlings mock-treated with dimethylsulfoxide (final concentration 0.1%) served as the control. All samples are harvested and stored at −80°C until the RNA was extracted.

Real-time PCR analysis was performed using gene-specific primers as described earlier [97]. The primer sequences are listed in Additional file 15. There are at least three biological replicates of each treatment and duplicate QRT-PCR analyses for each sample. Total RNA was prepared using RNeasy plant Mini Kit (Qiagen) with RNase-free DNase I (Qiagen). Approximately 2 μg of total RNA was used as template for first-strand cDNA synthesis, which was performed by SuperScript III RT (Invitrogen, Carlsbad, CA, USA) with oligo(dT)15 primers in a reaction volume of 20 μl. The RT reaction was diluted 1:10 and 5 μl used in the amplification with the specific PCR primers. Quantitative RT-PCR analysis was performed using an ABI 7500 real-time detection system and SYBER Green Dye (ABI, Foster City, CA). PCR amplification was performed in duplicate. The RNA expressions were normalized with the internal control, ACTIN 1 (ACT1) or 18 s rRNA [97] to ensure the equal amount of cDNA. The mRNA levels for each candidate gene in different tissue samples were calculated using the ΔΔCT method.

## Competing interests

The authors declare that they have no competing interests.

## Authors’ contributions

H.C. carried out most of the bioinformatics analyses and wrote the entire manuscript. C.P.L. coordinated and supervised all the analyses and contributed to writing of the manuscript. L.M.H. carried out hormone treatment experiments from plant material to qRT-PCR. J.H.L. carried out the protein structure modelling study and revised the final text of the manuscript. J.F.S., the principal investigator of the project, provided the concept and strategic planning for the entire study, and directed and supervised the completion of the manuscript. All authors read and approved the final version of the manuscript.

## Supplementary Material

Additional file 1 Characteristics of the rice GDSL esterase/lipase gene family. The gene name, locus ID MSU Osa1 RGAP Release 6.1, open reading frame length, protein length, FL-cDNA, genomic sequences and CDS accession numbers, and isoelectric points of all 114 *OsGELP* genes are given. Click here for file

Additional file 2 Expression evidence for the *OsGELP* rice genes. The *OsGELP* gene names, locus ID, MPSS signature sequences, FL-cDNA number, total quantity of mapped ESTs, and the presence of microarray data from Genevestigator for each of 153 transcripts (including alternative spliced models) of the 114 *OsGELP* genes are given. Click here for file

Additional file 3 Pattern of the *OsGELP* gene clusters on rice chromosomes. (A) The order and clusters’ structures of 54 *OsGELP* genes on rice chromosomes. (B) The pattern of the *OsGELP* gene clusters on rice chromosomes, which are interrupted by unrelated genes. Click here for file

Additional file 4 The *OsGELP* genes present on duplicated chromosomal segments of rice *O. sativa* L. ssp. *japonica.* The segmental duplicated of the *OsGELP* genes, with their BLASTP E-value, locus ID, and chromosome coordinates, are present according to the RGAP Segmental Genome Duplication of Rice, with the maximal length distance permitted between collinear gene pairs of 500 kb. Click here for file

Additional file 5 The *OsGELP* genes resulting from duplications after the eudicots-monocots split, and preceding the sorghum and rice speciation. Such *OsGELP* genes with their gene names and chromosome locations are presented. Click here for file

Additional file 6 Gene structure of the *OsGELP* genes. The exon/intron structures of a total of 153 transcripts (including alternative spliced models) of the 114 *OsGELP* genes are presented. Green and blue boxes represent exon and UTR regions, respectively, and solid lines indicate intron regions. The length of the boxes and lines are scaled based on the length of genes. Click here for file

Additional file 7 Chromosomal location and exon/intron number for the *OsGELP* rice genes. The *OsGELP* gene names, locus ID, chromosomal location, open reading frame and genomic sequence length, and numbers of exons/introns for each 114 GDSL esterase/lipase genes are given. Click here for file

Additional file 8 Identification of the repetitive DNA sequences within the *OsGELP* rice gene family. Diverse types of repetitive sequences with names, length (bp), and their positions and numbers for the 71 *OsGELP* genes are shown. The list of the repetitive DNA sequences present in the *OsGELP* genes is displayed in the order of their appearance from 5′- to 3′-end. Click here for file

Additional file 9 The 18 OsGELP proteins that were excluded from phylogenetic analysis. The GDSL esterase/lipase gene names, protein length, and the presence of five strictly conserved residues Ser-Gly-Asn-Asp-His in conserved blocks I, II, III, and V for 18 excluded genes are given. The presence of the consensus GDSL blocks is indicated by filled coloured boxes, and blank boxes display the absence of consensus alignment between them and other OsGELP proteins. Click here for file

Additional file 10 Physiological role, properties, and putative functions of plant GDSL esterases/lipases . The name, accession number, properties, and putative functions, as well as general biological roles of 24 plant GDSL esterases/lipases, whose putative functions have been elucidated recently and were adjoined into the original rice OsGELP family NJ tree, are listed. The coloured table divides 24 plant GDSL esterase/lipase proteins into three parts according to their major biological roles: secondary metabolism, plant development and morphogenesis, and defence and are shaded in blue, green, and light pink, respectively. In total, 50 OsGELP proteins with their names and percentage of similarity to every plant homolog or ortholog protein, whose function was revealed recently, along with phylogenetic subclade specificity to the tree from Figure 4, are given. Click here for file

Additional file 11 Putative conserved motifs predicted in the OsGELP and known plant GDSL esterase/lipase proteins. The consensus sequence, regular expression, amino acid length, number of the OsGELP proteins containing the motif, and E-value of each 45 predicted motifs are given. The overall height of each column in the motif LOGO indicates sequence conservation at that position, whereas the height of symbols within each column presents relative frequency of the corresponding amino acid. GDSL lipase consensus block distribution is as follows: block I is located in motif 3, block II in motif 5, block III in motif 6, and block V in motif 2. Four strictly conserved catalytic residues Ser-Gly-Asn-HisxxAsp from conserved blocks I, II, III, and V are coloured red in regular expression of corresponding motifs. Regular expression pattern sequences that are coloured in blue and green represent possible sequences for secondary structure elements like helix or sheet, respectively. Click here for file

Additional file 12 Differential expression of rice *OsGELP* genes in response to plant hormone cytokinin. A. Comparison of the fold expression difference for the 17 representative genes under cytokinin (tZ, BAP, and KT) treatment for results from the real-time PCR, and the microarray data obtained from Genevestigator database are given. B. Real-time PCR analysis of representative *OsGELP* genes and their differential expression during cytokinin (tZ, BAP, and KT) treatment are shown. The mRNA levels for each gene in different tissue samples were calculated relative to its expression in control seedlings. The error bars represent standard deviation. Click here for file

Additional file 13 The rice GDSL esterase/lipase genes excluded from the general list of the *OsGELP* candidates. The locus ID, ORF length, predicted protein length, the presence of GDSL-lipase domain with confidence (E-value), description, and cDNA support of all 19 excluded genes are given. Click here for file

Additional file 14 Motifs represent 13 highly conserved OsGELP protein alignment blocks used for phylogenetic analysis. The consensus sequence, regular expression, length (amino acids), number of the OsGELP proteins containing the motif, and E-value of each of predicted motifs are given. The overall height of each column in the motif LOGO indicates sequence conservation at that position, whereas the height of symbols within each column presents relative frequency of the corresponding amino acid. GDSL lipase consensus block distribution is as follows: motif 3 is located in block I, motif 5 is in block II, motif 6 is in block III, and motif 2 is in block V. Four strictly conserved catalytic residues Ser-Gly-Asn-HisxxAsp from conserved blocks I, II, III, and V are coloured red in the regular expression of representative motif. Regular expression pattern sequences that are coloured in blue and green represent possible sequences for secondary structure elements like helix or sheet, respectively. Click here for file

Additional file 15 Primer sequences used for real-time PCR analysis. The *OsGELP* gene names and sequences of PCR primers used in the quantitative RT PCRs to verify gene expression levels are listed. Click here for file

## References

[B1] Brick DJ Brumlik MJ Buckley JT Cao J-X Davies PC Misra S Tranbarger TJ Upton C A new family of lipolytic plant enzymes with members in rice, arabidopsis and maize FEBS Lett 1995 377 3 475 480 10.1016/0014-5793(95)01405-5 8549779

[B2] Upton C Buckley JT A new family of lipolytic enzymes? Trends in Biochemical Sciences 1995 20 5 178 179 10.1016/S0968-0004(00)89002-7 7610479

[B3] Akoh CC Lee GC Liaw YC Huang TH Shaw JF GDSL family of serine esterases/lipases Prog Lipid Res 2004 43 6 534 552 10.1016/j.plipres.2004.09.002 15522763

[B4] Molgaard A Kauppinen S Larsen S Rhamnogalacturonan acetylesterase elucidates the structure and function of a new family of hydrolases Structure 2000 8 4 373 383 10.1016/S0969-2126(00)00118-0 10801485

[B5] Lee Y-L Chen JC Shaw J-F The Thioesterase I ofEscherichia coliHas Arylesterase Activity and Shows Stereospecificity for Protease Substrates Biochem Biophys Res Commun 1997 231 2 452 456 10.1006/bbrc.1997.5797 9070299

[B6] Shaw JF Chang RC Chuang KH Yen YT Wang YJ Wang FG Nucleotide sequence of a novel arylesterase gene from Vibro mimicus and characterization of the enzyme expressed in Escherichia coli Biochem J 1994 298 Pt 3 675 680 8141782 10.1042/bj2980675 PMC1137913

[B7] Cho H Cronan JE “Protease I” of Escherichia coli functions as a thioesterase in vivo J Bacteriol 1994 176 6 1793 1795 8132479 10.1128/jb.176.6.1793-1795.1994 PMC205273

[B8] Chang RC Chen JC Shaw JF Vibrio mimicus arylesterase has thioesterase and chymotrypsin-like activity Biochem Biophys Res Commun 1995 213 2 475 483 10.1006/bbrc.1995.2156 7646502

[B9] Chang RC Chen JC Shaw JF Site-directed mutagenesis of a novel serine arylesterase from Vibrio mimicus identifies residues essential for catalysis Biochem Biophys Res Commun 1996 221 2 477 483 10.1006/bbrc.1996.0620 8619880

[B10] Lee Y-L Su M-S Huang T-H Shaw J-F C-terminal his-tagging results in substrate specificity changes of the thioesterase I from Escherichia coli 1999 Springer, Heidelberg, ALLEMAGNE

[B11] Wilhelm S Tommassen J Jaeger KE A novel lipolytic enzyme located in the outer membrane of Pseudomonas aeruginosa J Bacteriol 1999 181 22 6977 6986 10559163 10.1128/jb.181.22.6977-6986.1999 PMC94172

[B12] Huang YT Liaw YC Gorbatyuk VY Huang TH Backbone dynamics of Escherichia coli thioesterase/protease I: evidence of a flexible active-site environment for a serine protease J Mol Biol 2001 307 4 1075 1090 10.1006/jmbi.2001.4539 11286557

[B13] Tyukhtenko SI Litvinchuk AV Chang CF Leu RJ Shaw JF Huang TH NMR studies of the hydrogen bonds involving the catalytic triad of Escherichia coli thioesterase/protease I FEBS Lett 2002 528 1–3 203 206 12297305 10.1016/s0014-5793(02)03308-2

[B14] Vujaklija D Schroder W Abramic M Zou P Lescic I Franke P Pigac J A novel streptomycete lipase: cloning, sequencing and high-level expression of the Streptomyces rimosus GDS(L)-lipase gene Arch Microbiol 2002 178 2 124 130 10.1007/s00203-002-0430-6 12115057

[B15] Talker-Huiber D Jose J Glieder A Pressnig M Stubenrauch G Schwab H Esterase EstE from Xanthomonas vesicatoria ( Xv_EstE) is an outer membrane protein capable of hydrolyzing long-chain polar esters Appl Microbiol Biotechnol 2003 61 5–6 479 487 12764562 10.1007/s00253-003-1227-5

[B16] Tyukhtenko SI Litvinchuk AV Chang CF Lo YC Lee SJ Shaw JF Liaw YC Huang TH Sequential structural changes of Escherichia coli thioesterase/protease I in the serial formation of Michaelis and tetrahedral complexes with diethyl p-nitrophenyl phosphate Biochemistry 2003 42 27 8289 8297 10.1021/bi027246w 12846577

[B17] Yang TH Pan JG Seo YS Rhee JS Use of Pseudomonas putida EstA as an Anchoring Motif for Display of a Periplasmic Enzyme on the Surface of Escherichia coli Appl Environ Microbiol 2004 70 12 6968 6976 10.1128/AEM.70.12.6968-6976.2004 15574889 PMC535197

[B18] Hausmann S Jaeger KE Timmis KN Lipolytic Enzymes from Bacteria Handbook of Hydrocarbon and Lipid Microbiology 2010 Springer, Berlin Heidelberg 1099 1126

[B19] Yoshida S Mackie RI Cann IK Biochemical and domain analyses of FSUAxe6B, a modular acetyl xylan esterase, identify a unique carbohydrate binding module in Fibrobacter succinogenes S85 J Bacteriol 2010 192 2 483 493 10.1128/JB.00935-09 19897648 PMC2805333

[B20] Yu S Zheng B Zhao X Feng Y Gene cloning and characterization of a novel thermophilic esterase from Fervidobacterium nodosum Rt17-B1 Acta Biochim Biophys Sin (Shanghai) 2010 42 4 288 295 10.1093/abbs/gmq020 20383468

[B21] Wei Y Schottel JL Derewenda U Swenson L Patkar S Derewenda ZS A novel variant of the catalytic triad in the Streptomyces scabies esterase Nat Struct Biol 1995 2 3 218 223 10.1038/nsb0395-218 7773790

[B22] Lin TH Chen C Huang RF Lee YL Shaw JF Huang TH Multinuclear NMR resonance assignments and the secondary structure of Escherichia coli thioesterase/protease I: a member of a new subclass of lipolytic enzymes J Biomol NMR 1998 11 4 363 380 10.1023/A:1008226515482 9691282

[B23] Li J Derewenda U Dauter Z Smith S Derewenda ZS Crystal structure of the Escherichia coli thioesterase II, a homolog of the human Nef binding enzyme Nat Struct Biol 2000 7 7 555 559 10.1038/76776 10876240

[B24] Lo YC Lee YL Shaw JF Liaw YC Crystallization and preliminary X-ray crystallographic analysis of thioesterase I from Escherichia coli Acta Crystallogr D: Biol Crystallogr 2000 56 Pt 6 756 757 10818355 10.1107/s0907444900004339

[B25] Lo YC Lin SC Shaw JF Liaw YC Crystal structure of Escherichia coli thioesterase I/protease I/lysophospholipase L1: consensus sequence blocks constitute the catalytic center of SGNH-hydrolases through a conserved hydrogen bond network J Mol Biol 2003 330 3 539 551 10.1016/S0022-2836(03)00637-5 12842470

[B26] Cheeseman JD Tocilj A Park S Schrag JD Kazlauskas RJ Structure of an aryl esterase from Pseudomonas fluorescens Acta Crystallogr D: Biol Crystallogr 2004 60 Pt 7 1237 1243 15213385 10.1107/S0907444904010522

[B27] Mathews I Soltis M Saldajeno M Ganshaw G Sala R Weyler W Cervin MA Whited G Bott R Structure of a Novel Enzyme That Catalyzes Acyl Transfer to Alcohols in Aqueous Conditions‡ Biochemistry 2007 46 31 8969 8979 10.1021/bi7002444 17636869

[B28] van den Berg B Crystal structure of a full-length autotransporter J Mol Biol 2010 396 3 627 633 10.1016/j.jmb.2009.12.061 20060837

[B29] Ling H Sequence analysis of GDSL lipase gene family in Arabidopsis thaliana Pak J Biol Sci 2008 11 5 763 767 10.3923/pjbs.2008.763.767 18819574

[B30] Volokita M Rosilio-Brami T Rivkin N Zik M Combining comparative sequence and genomic data to ascertain phylogenetic relationships and explore the evolution of the large GDSL-lipase family in land-plants Mol Biol Evol 2010 28 1 551 565 20801908 10.1093/molbev/msq226

[B31] Youens-Clark K Buckler E Casstevens T Chen C Declerck G Derwent P Dharmawardhana P Jaiswal P Kersey P Karthikeyan AS Gramene database in 2010: updates and extensions Nucleic Acids Res 2010 39 Database issue D1085 D1094 21076153 10.1093/nar/gkq1148 PMC3013721

[B32] Ouyang S Zhu W Hamilton J Lin H Campbell M Childs K Thibaud-Nissen F Malek RL Lee Y Zheng L The TIGR Rice Genome Annotation Resource: improvements and new features Nucleic Acids Res 2007 35 Database issue D883 D887 17145706 10.1093/nar/gkl976 PMC1751532

[B33] Zhang Z Ober JA Kliebenstein DJ The gene controlling the quantitative trait locus EPITHIOSPECIFIER MODIFIER1 alters glucosinolate hydrolysis and insect resistance in Arabidopsis Plant Cell 2006 18 6 1524 1536 10.1105/tpc.105.039602 16679459 PMC1475484

[B34] Agee AE Surpin M Sohn EJ Girke T Rosado A Kram BW Carter C Wentzell AM Kliebenstein DJ Jin HC MODIFIED VACUOLE PHENOTYPE1 is an Arabidopsis myrosinase-associated protein involved in endomembrane protein trafficking Plant Physiol 2010 152 1 120 132 10.1104/pp.109.145078 19880612 PMC2799351

[B35] Oh IS Park AR Bae MS Kwon SJ Kim YS Lee JE Kang NY Lee S Cheong H Park OK Secretome analysis reveals an Arabidopsis lipase involved in defense against Alternaria brassicicola Plant Cell 2005 17 10 2832 2847 10.1105/tpc.105.034819 16126835 PMC1242276

[B36] Kwon SJ Jin HC Lee S Nam MH Chung JH Kwon SI Ryu CM Park OK GDSL lipase-like 1 regulates systemic resistance associated with ethylene signaling in Arabidopsis The Plant Journal 2009 58 2 235 245 10.1111/j.1365-313X.2008.03772.x 19077166

[B37] Lee DS Kim BK Kwon SJ Jin HC Park OK Arabidopsis GDSL lipase 2 plays a role in pathogen defense via negative regulation of auxin signaling Biochem Biophys Res Commun 2009 379 4 1038 1042 10.1016/j.bbrc.2009.01.006 19146828

[B38] Lee KA Cho TJ Characterization of a salicylic acid- and pathogen-induced lipase-like gene in Chinese cabbage J Biochem Mol Biol 2003 36 5 433 441 10.5483/BMBRep.2003.36.5.433 14536025

[B39] Hong JK Choi HW Hwang IS Kim DS Kim NH du Choi S Kim YJ Hwang BK Function of a novel GDSL-type pepper lipase gene, CaGLIP1, in disease susceptibility and abiotic stress tolerance Planta 2008 227 3 539 558 10.1007/s00425-007-0637-5 17929052

[B40] Kim KJ Lim JH Kim MJ Kim T Chung HM Paek KH GDSL-lipase1 (CaGL1) contributes to wound stress resistance by modulation of CaPR-4 expression in hot pepper Biochem Biophys Res Commun 2008 374 4 693 698 10.1016/j.bbrc.2008.07.120 18680725

[B41] Naranjo MA Forment J Roldan M Serrano R Vicente O Overexpression of Arabidopsis thaliana LTL1, a salt-induced gene encoding a GDSL-motif lipase, increases salt tolerance in yeast and transgenic plants Plant Cell Environ 2006 29 10 1890 1900 10.1111/j.1365-3040.2006.01565.x 16930315

[B42] Takahashi K Shimada T Kondo M Tamai A Mori M Nishimura M Hara-Nishimura I Ectopic expression of an esterase, which is a candidate for the unidentified plant cutinase, causes cuticular defects in Arabidopsis thaliana Plant Cell Physiol 2010 51 1 123 131 10.1093/pcp/pcp173 19996150

[B43] Updegraff EP Zhao F Preuss D The extracellular lipase EXL4 is required for efficient hydration of Arabidopsis pollen Sex Plant Reprod 2009 22 3 197 204 10.1007/s00497-009-0104-5 20033440

[B44] Kram BW Bainbridge EA Perera MA Carter C Identification, cloning and characterization of a GDSL lipase secreted into the nectar of Jacaranda mimosifolia Plant Mol Biol 2008 68 1–2 173 183 18553138 10.1007/s11103-008-9361-1

[B45] Reina JJ Guerrero C Heredia A Isolation, characterization, and localization of AgaSGNH cDNA: a new SGNH-motif plant hydrolase specific to Agave americana L. leaf epidermis J Exp Bot 2007 58 11 2717 2731 10.1093/jxb/erm136 17609535

[B46] Pringle D Dickstein R Purification of ENOD8 proteins from Medicago sativa root nodules and their characterization as esterases Plant Physiol Biochem 2004 42 1 73 79 10.1016/j.plaphy.2003.10.004 15061087

[B47] Cummins I Edwards R Purification and cloning of an esterase from the weed black-grass (Alopecurus myosuroides), which bioactivates aryloxyphenoxypropionate herbicides The Plant Journal 2004 39 6 894 904 10.1111/j.1365-313X.2004.02174.x 15341632

[B48] Clauss K Baumert A Nimtz M Milkowski C Strack D Role of a GDSL lipase-like protein as sinapine esterase in Brassicaceae Plant J 2008 53 5 802 813 10.1111/j.1365-313X.2007.03374.x 18036206

[B49] Ruppert M Woll J Giritch A Genady E Ma X Stockigt J Functional expression of an ajmaline pathway-specific esterase from Rauvolfia in a novel plant-virus expression system Planta 2005 222 5 888 898 10.1007/s00425-005-0031-0 16133216

[B50] de La Torre F Sampedro J Zarra I Revilla G AtFXG1, an Arabidopsis gene encoding alpha-L-fucosidase active against fucosylated xyloglucan oligosaccharides Plant Physiol 2002 128 1 247 255 10.1104/pp.010508 11788770 PMC148987

[B51] Kandzia R Grimm R Eckerskorn C Lindemann P Luckner M Purification and characterization of lanatoside 15'-O-acetylesterase from Digitalis lanata Ehrh Planta 1998 204 3 383 389 10.1007/s004250050270 9530881

[B52] Yamamoto K Oguri S Momonoki YS Characterization of trimeric acetylcholinesterase from a legume plant, Macroptilium atropurpureum Urb Planta 2008 227 4 809 822 10.1007/s00425-007-0658-0 18046576

[B53] Sagane Y Nakagawa T Yamamoto K Michikawa S Oguri S Momonoki YS Molecular characterization of maize acetylcholinesterase: a novel enzyme family in the plant kingdom Plant Physiol 2005 138 3 1359 1371 10.1104/pp.105.062927 15980188 PMC1176409

[B54] Yamamoto K Momonoki YS Subcellular localization of overexpressed maize AChE gene in rice plant Plant Signal Behav 2008 3 8 576 577 10.4161/psb.3.8.5732 19704473 PMC2634501

[B55] Yamamoto K Oguri S Chiba S Momonoki YS Molecular cloning of acetylcholinesterase gene from Salicornia europaea L Plant Signal Behav 2009 4 5 361 366 10.4161/psb.4.5.8360 19816117 PMC2676743

[B56] Riemann M Gutjahr C Korte A Danger B Muramatsu T Bayer U Waller F Furuya M Nick P GER1, a GDSL motif-encoding gene from rice is a novel early light- and jasmonate-induced gene Plant Biol (Stuttg) 2007 9 1 32 40 10.1055/s-2006-924561 17048141

[B57] Park JJ Jin P Yoon J Yang JI Jeong HJ Ranathunge K Schreiber L Franke R Lee IJ An G Mutation in Wilted Dwarf and Lethal 1 (WDL1) causes abnormal cuticle formation and rapid water loss in rice Plant Mol Biol 2010 74 1–2 91 103 20593223 10.1007/s11103-010-9656-x

[B58] Hruz T Laule O Szabo G Wessendorp F Bleuler S Oertle L Widmayer P Gruissem W Zimmermann P Genevestigator v3: a reference expression database for the meta-analysis of transcriptomes Adv Bioinformatics 2008 2008 420747 19956698 10.1155/2008/420747 PMC2777001

[B59] Campbell MA Haas BJ Hamilton JP Mount SM Buell CR Comprehensive analysis of alternative splicing in rice and comparative analyses with Arabidopsis BMC Genomics 2006 7 327 10.1186/1471-2164-7-327 17194304 PMC1769492

[B60] Kikuchi S Satoh K Nagata T Kawagashira N Doi K Kishimoto N Yazaki J Ishikawa M Yamada H Ooka H Collection, mapping, and annotation of over 28,000 cDNA clones from japonica rice Science 2003 301 5631 376 379 10.1126/science.1081288 12869764

[B61] Reddy AS Alternative splicing of pre-messenger RNAs in plants in the genomic era Annu Rev Plant Biol 2007 58 267 294 10.1146/annurev.arplant.58.032806.103754 17222076

[B62] Koonin EV Orthologs, paralogs, and evolutionary genomics Annu Rev Genet 2005 39 309 338 10.1146/annurev.genet.39.073003.114725 16285863

[B63] Abdelkafi S Ogata H Barouh N Fouquet B Lebrun R Pina M Scheirlinckx F Villeneuve P Carriere F Identification and biochemical characterization of a GDSL-motif carboxylester hydrolase from Carica papaya latex Biochim Biophys Acta 2009 1791 11 1048 1056 10.1016/j.bbalip.2009.06.002 19555778

[B64] Sigrist CJ Cerutti L de Castro E Langendijk-Genevaux PS Bulliard V Bairoch A Hulo N PROSITE, a protein domain database for functional characterization and annotation Nucleic Acids Res 2010 38 Database issue D161 D166 19858104 10.1093/nar/gkp885 PMC2808866

[B65] UniProt Consortium Reorganizing the protein space at the Universal Protein Resource (UniProt) Nucleic Acids Res 2012 40 Database issue D71 D75 22102590 10.1093/nar/gkr981 PMC3245120

[B66] Trifonov EN Frenkel ZM Evolution of protein modularity Curr Opin Struct Biol 2009 19 3 335 340 10.1016/j.sbi.2009.03.007 19386484

[B67] Bhattacharyya RP Remenyi A Yeh BJ Lim WA Domains, motifs, and scaffolds: the role of modular interactions in the evolution and wiring of cell signaling circuits Annu Rev Biochem 2006 75 655 680 10.1146/annurev.biochem.75.103004.142710 16756506

[B68] Kelley LA Sternberg MJE Protein structure prediction on the Web: a case study using the Phyre server Nat Protoc 2009 4 3 363 371 10.1038/nprot.2009.2 19247286

[B69] Cannon SB Mitra A Baumgarten A Young ND May G The roles of segmental and tandem gene duplication in the evolution of large gene families in Arabidopsis thaliana BMC Plant Biol 2004 4 10 10.1186/1471-2229-4-10 15171794 PMC446195

[B70] Kurata N Hirano H-Y, Sano Y, Hirai A, Sasaki T Chromosome and Genome Evolution in Rice Rice Biology in the Genomics Era 2008 Springer, Berlin Heidelberg 235 245 vol. 62

[B71] Yu J Wang J Lin W Li S Li H Zhou J Ni P Dong W Hu S Zeng C The Genomes of Oryza sativa: a history of duplications PLoS Biol 2005 3 2 e38 10.1371/journal.pbio.0030038 15685292 PMC546038

[B72] Guo X Xu G Zhang Y Wen X Hu W Fan L Incongruent evolution of chromosomal size in rice Genet Mol Res 2006 5 2 373 389 16819716

[B73] Rose AB Intron-mediated regulation of gene expression Curr Top Microbiol Immunol 2008 326 277 290 10.1007/978-3-540-76776-3_15 18630758

[B74] Lin H Zhu W Silva JC Gu X Buell CR Intron gain and loss in segmentally duplicated genes in rice Genome Biol 2006 7 5 R41 10.1186/gb-2006-7-5-r41 16719932 PMC1779517

[B75] Roy SW Penny D Patterns of intron loss and gain in plants: intron loss-dominated evolution and genome-wide comparison of O. sativa and A. thaliana Mol Biol Evol 2007 24 1 171 181 17065597 10.1093/molbev/msl159

[B76] Irimia M Roy SW Spliceosomal introns as tools for genomic and evolutionary analysis Nucleic Acids Res 2008 36 5 1703 1712 10.1093/nar/gkn012 18263615 PMC2275149

[B77] Lecharny A Boudet N Gy I Aubourg S Kreis M Introns in, introns out in plant gene families: a genomic approach of the dynamics of gene structure J Struct Funct Genomics 2003 3 1–4 111 116 12836690

[B78] Argueso CT Ferreira FJ Kieber JJ Environmental perception avenues: the interaction of cytokinin and environmental response pathways Plant Cell Environ 2009 32 9 1147 1160 10.1111/j.1365-3040.2009.01940.x 19183294

[B79] Hu H You J Fang Y Zhu X Qi Z Xiong L Characterization of transcription factor gene SNAC2 conferring cold and salt tolerance in rice Plant Mol Biol 2008 67 1–2 169 181 18273684 10.1007/s11103-008-9309-5

[B80] Arif SA Hamilton RG Yusof F Chew NP Loke YH Nimkar S Beintema JJ Yeang HY Isolation and characterization of the early nodule-specific protein homologue (Hev b 13), an allergenic lipolytic esterase from Hevea brasiliensis latex J Biol Chem 2004 279 23 23933 23941 10.1074/jbc.M309800200 15024009

[B81] Lin H Ouyang S Egan A Nobuta K Haas BJ Zhu W Gu X Silva JC Meyers BC Buell CR Characterization of paralogous protein families in rice BMC Plant Biol 2008 8 18 10.1186/1471-2229-8-18 18284697 PMC2275729

[B82] Benson DA Karsch-Mizrachi I Lipman DJ Ostell J Sayers EW GenBank Nucleic Acids Res 2010 38 Database issue D46 D51 19910366 10.1093/nar/gkp1024 PMC2808980

[B83] Pagni M Ioannidis V Cerutti L Zahn-Zabal M Jongeneel CV Hau J Martin O Kuznetsov D Falquet L MyHits: improvements to an interactive resource for analyzing protein sequences Nucleic Acids Res 2007 35 Web Server issue W433 W437 17545200 10.1093/nar/gkm352 PMC1933190

[B84] Satoh K Doi K Nagata T Kishimoto N Suzuki K Otomo Y Kawai J Nakamura M Hirozane-Kishikawa T Kanagawa S Gene organization in rice revealed by full-length cDNA mapping and gene expression analysis through microarray PLoS One 2007 2 11 e1235 10.1371/journal.pone.0001235 18043742 PMC2084198

[B85] Kanehisa M Goto S Hattori M Aoki-Kinoshita KF Itoh M Kawashima S Katayama T Araki M Hirakawa M From genomics to chemical genomics: new developments in KEGG Nucleic Acids Res 2006 34 Database issue D354 D357 16381885 10.1093/nar/gkj102 PMC1347464

[B86] Rouard M Guignon V Aluome C Laporte MA Droc G Walde C Zmasek CM Perin C Conte MG GreenPhylDB v2.0: comparative and functional genomics in plants Nucleic Acids Res 2011 39 Database issue D1095 D1102 20864446 10.1093/nar/gkq811 PMC3013755

[B87] Guo AY Zhu QH Chen X Luo JC GSDS: a gene structure display server Yi Chuan 2007 29 8 1023 1026 17681935

[B88] RepeatMasker Open-3.0, http://www.repeatmasker.org

[B89] Tamura K Dudley J Nei M Kumar S MEGA4: Molecular Evolutionary Genetics Analysis (MEGA) software version 4.0 Mol Biol Evol 2007 24 8 1596 1599 10.1093/molbev/msm092 17488738

[B90] Bailey TL Elkan C Fitting a mixture model by expectation maximization to discover motifs in biopolymers Proc Int Conf Intell Syst Mol Biol 1994 2 28 36 7584402

[B91] Bailey TL Boden M Buske FA Frith M Grant CE Clementi L Ren J Li WW Noble WS MEME SUITE: tools for motif discovery and searching Nucleic Acids Res 2009 37 Web Server issue W202 W208 19458158 10.1093/nar/gkp335 PMC2703892

[B92] The PyMOL Molecular Graphics System, http://www.pymol.org/

[B93] Bond CS TopDraw: a sketchpad for protein structure topology cartoons Bioinformatics 2003 19 2 311 312 10.1093/bioinformatics/19.2.311 12538265

[B94] Rice MPSS Database, http://mpss.udel.edu/rice/

[B95] Hirose N Makita N Kojima M Kamada-Nobusada T Sakakibara H Overexpression of a Type-A Response Regulator Alters Rice Morphology and Cytokinin Metabolism Plant and Cell Physiology 2007 48 3 523 539 10.1093/pcp/pcm022 17293362

[B96] Yoshida S Forno DA Cock JH Gomez KA Laboratory Manual for Physiological Studies of Rice 1976 The International Rice Research Institute, Los Baños, Philippines

[B97] Jain M Nijhawan A Tyagi AK Khurana JP Validation of housekeeping genes as internal control for studying gene expression in rice by quantitative real-time PCR Biochem Biophys Res Commun 2006 345 2 646 651 10.1016/j.bbrc.2006.04.140 16690022

