# Supplementary material for: Multifunctionality and diversity of GDSL esterase/lipase gene family in rice (Oryza sativa L. japonica) genome: new insights from bioinformatics analysis
Source: BMC Genomics. 2012 Jul 15;13:309. doi: 10.1186/1471-2164-13-309 (PMC3412167; doi:10.1186/1471-2164-13-309)
Supplement: Additional file 1 — Characteristics of the rice GDSL esterase/lipase gene family. The gene name, locus ID MSU Osa1 RGAP Release 6.1, open reading frame length, protein length, FL-cDNA, genomic sequences and CDS accession numbers, and isoelectric points of all 114 OsGELP genes are given. [file 1471-2164-13-309-S1.doc]

**Additional file 1.** Characteristics of the rice GDSL esterase/lipase gene family.

| **Gene**  **name** | **Locus ID** | **CDS accession number** | **Genomic**  **DNA accession number** | **cDNA Accession number** | **Nucleotide length (bp)** | **Predicted protein**  **length (aa)** | **Protein Mass (Da)** | **Predicted isoelectric point** | **Pham E -value of PF00657** | **Other name** |
| --- | --- | --- | --- | --- | --- | --- | --- | --- | --- | --- |
| *OsGELP1* | [Os01g11570](http://rice.plantbiology.msu.edu/cgi-bin/ORF_infopage.cgi?&orf=LOC_Os01g11570) | 13101.[m01240](http://rice.plantbiology.msu.edu/cgi-bin/sequence_display.cgi?orf=13101.m01240) | [13101](http://rice.plantbiology.msu.edu/cgi-bin/sequence_display.cgi?orf=13101.m01240).t01018 | [AK061229.1](http://www.ncbi.nlm.nih.gov/UniGene/seq.cgi?ORG=Os&SID=16416774) | 717 | 239 | 26475.06 | 4.94 | 5-203 |  |
| *OsGELP2a* | [Os01g11620](http://rice.plantbiology.msu.edu/cgi-bin/ORF_infopage.cgi?&orf=LOC_Os01g11620).1 | [13101](http://rice.plantbiology.msu.edu/cgi-bin/sequence_display.cgi?orf=13101.m01248).m01248 | [13101](http://rice.plantbiology.msu.edu/cgi-bin/sequence_display.cgi?orf=13101.m01248).t01023 | n/a | 1101 | 367 | 38951.22 | 7.15 | 168-354 |  |
| *OsGELP2b* | [Os01g11620](http://rice.plantbiology.msu.edu/cgi-bin/ORF_infopage.cgi?orf=13101.m01247).2 | [13101.m01247](http://rice.plantbiology.msu.edu/cgi-bin/sequence_display.cgi?orf=13101.m01247) | [13101.t01023](http://rice.plantbiology.msu.edu/cgi-bin/sequence_display.cgi?orf=13101.m01247) | [AK103614](http://rapdb.dna.affrc.go.jp/cgi-bin/gbrowse_details/IRGSP40?name=AK103614;class=seq_id) | 1050 | 367 | 37181.12 | 6.70 | 203-337 |  |
| *OsGELP3a* | [Os01g11650](http://rice.plantbiology.msu.edu/cgi-bin/ORF_infopage.cgi?&orf=LOC_Os01g11650).1 | [13101.m01251](http://rice.plantbiology.msu.edu/cgi-bin/sequence_display.cgi?orf=13101.m01251) | [13101.t01026](http://rice.plantbiology.msu.edu/cgi-bin/sequence_display.cgi?orf=13101.m01251) | [AK065001](http://www.ncbi.nlm.nih.gov/entrez/viewer.fcgi?view=gb&query_key=98&db=nuccore&dopt=GenBank&WebEnv=0z1zaJcVb-hzBOfhdkleeTpiTCa_yPl4zFJw_wMf7PinAwjwZs-7lGd3mcR@26434B207772B780_0086SID&WebEnvRq=1&term=&tool=query&qty=1) | 1119 | 373 | 41102.99 | 7.97 | 31-356 |  |
| *OsGELP3b* | [Os01g11650.2](http://rice.plantbiology.msu.edu/cgi-bin/ORF_infopage.cgi?orf=13101.m01252) | [13101.m01252](http://rice.plantbiology.msu.edu/cgi-bin/sequence_display.cgi?orf=13101.m01252) | [13101.t01026](http://rice.plantbiology.msu.edu/cgi-bin/sequence_display.cgi?orf=13101.m01252) | [AK069801](http://www.ncbi.nlm.nih.gov/nuccore/32979825?report=genbank) | 1032 | 373 | 38083.13 | 9.12 | 31-261 |  |
| *OsGELP4* | [Os01g11660](http://rice.plantbiology.msu.edu/cgi-bin/ORF_infopage.cgi?&orf=LOC_Os01g11660) | [13101.m01253](http://rice.plantbiology.msu.edu/cgi-bin/sequence_display.cgi?orf=13101.m01253) | [13101.t01027](http://rice.plantbiology.msu.edu/cgi-bin/sequence_display.cgi?orf=13101.m01253) | [AK100754](http://www.ncbi.nlm.nih.gov/entrez/viewer.fcgi?db=nucleotide&val=AK100754) | 1095 | 365 | 39520.07 | 6.50 | 32-357 |  |
| *OsGELP5a* | [Os01g11700](http://rice.plantbiology.msu.edu/cgi-bin/ORF_infopage.cgi?&orf=LOC_Os01g11700).1 | [13101.m01258](http://rice.plantbiology.msu.edu/cgi-bin/sequence_display.cgi?orf=13101.m01258) | [13101.t01031](http://rice.plantbiology.msu.edu/cgi-bin/sequence_display.cgi?orf=13101.m01258) | n/a | 1131 | 377 | 40365.64 | 5.15 | 169-356 |  |
| *OsGELP5b* | [Os01g11700](http://rice.plantbiology.msu.edu/cgi-bin/ORF_infopage.cgi?orf=13101.m01257).2 | [12001.m07786](http://www.tigr.org/tigr-scripts/euk_manatee/shared/seq_display.cgi?db=osa1r5&orf=12001.m07786) | 13101.t01031 | [AK102384](http://www.ncbi.nlm.nih.gov/entrez/viewer.fcgi?db=nucleotide&val=AK102384)  [AK059318](http://www.ncbi.nlm.nih.gov/nuccore/32969336?report=genbank) | 915 | 377 | 32936.38 | 5.32 | 169-285 |  |
| *OsGELP6* | [Os01g11710](http://rice.plantbiology.msu.edu/cgi-bin/ORF_infopage.cgi?&orf=LOC_Os01g11710) | [13101.m01260](http://rice.plantbiology.msu.edu/cgi-bin/sequence_display.cgi?orf=13101.m01260) | [13101.t01033](http://rice.plantbiology.msu.edu/cgi-bin/sequence_display.cgi?orf=13101.m01260) | [AK059088](http://www.ncbi.nlm.nih.gov/entrez/viewer.fcgi?db=nucleotide&val=AK059088) | 1137 | 379 | 40748.06 | 8.51 | 44-367 |  |
| *OsGELP7* | [Os01g11730](http://rice.plantbiology.msu.edu/cgi-bin/ORF_infopage.cgi?&orf=LOC_Os01g11730) | [13101](http://rice.plantbiology.msu.edu/cgi-bin/sequence_display.cgi?orf=13101.m01262).m01262 | [13101.t01035](http://rice.plantbiology.msu.edu/cgi-bin/sequence_display.cgi?orf=13101.m01262) | [AK058429](http://www.ncbi.nlm.nih.gov/entrez/viewer.fcgi?db=nucleotide&val=AK058429) | 1194 | 398 | 42429.06 | 5.98 | 36-364 |  |
| *OsGELP8* | [Os01g11740](http://rice.plantbiology.msu.edu/cgi-bin/ORF_infopage.cgi?&orf=LOC_Os01g11740) | [13101](http://rice.plantbiology.msu.edu/cgi-bin/sequence_display.cgi?orf=13101.m01263).m01263 | [13101.t01036](http://rice.plantbiology.msu.edu/cgi-bin/sequence_display.cgi?orf=13101.m01263) | [AK107557](http://www.ncbi.nlm.nih.gov/entrez/viewer.fcgi?db=nucleotide&val=AK107557) | 1161 | 387 | 41946.88 | 7.16 | 179-368 |  |
| *OsGELP9* | [Os01g11750](http://rice.plantbiology.msu.edu/cgi-bin/ORF_infopage.cgi?&orf=LOC_Os01g11750) | [13101.m01264](http://rice.plantbiology.msu.edu/cgi-bin/sequence_display.cgi?orf=13101.m01264) | [13101](http://rice.plantbiology.msu.edu/cgi-bin/sequence_display.cgi?orf=13101.m01264).t01037 | n/a | 1149 | 383 | 41089.78 | 7.02 | 39-366 |  |
| *OsGELP10* | [Os01g11760](http://rice.plantbiology.msu.edu/cgi-bin/ORF_infopage.cgi?&orf=LOC_Os01g11760) | [13101.m01265](http://rice.plantbiology.msu.edu/cgi-bin/sequence_display.cgi?orf=13101.m01265) | [13101.t01038](http://rice.plantbiology.msu.edu/cgi-bin/sequence_display.cgi?orf=13101.m01265) | n/a | 1245 | 415 | 45341.88 | 8.57 | 240-398 |  |
| *OsGELP11* | [Os01g11790](http://rice.plantbiology.msu.edu/cgi-bin/ORF_infopage.cgi?&orf=LOC_Os01g11790) | [13101.m01268](http://rice.plantbiology.msu.edu/cgi-bin/sequence_display.cgi?orf=13101.m01268) | [13101.t01041](http://rice.plantbiology.msu.edu/cgi-bin/sequence_display.cgi?orf=13101.m01268) | n/a | 1230 | 410 | 44837.62 | 8.85 | 240-393 |  |
| *OsGELP12a* | [Os01g12320](http://rice.plantbiology.msu.edu/cgi-bin/ORF_infopage.cgi?&orf=LOC_Os01g12320) | [13101.m01328](http://rice.plantbiology.msu.edu/cgi-bin/sequence_display.cgi?orf=13101.m01328) | [13101.t01094](http://rice.plantbiology.msu.edu/cgi-bin/sequence_display.cgi?orf=13101.m01328) | [AK067352](http://www.ncbi.nlm.nih.gov/entrez/viewer.fcgi?db=nucleotide&val=AK067352) | 1140 | 380 | 40970.18 | 5.03 | 39-367 |  |
| *OsGELP12b* | [Os01g12320](http://rice.plantbiology.msu.edu/cgi-bin/ORF_infopage.cgi?orf=13101.m01329) | [13101.m01329](http://rice.plantbiology.msu.edu/cgi-bin/sequence_display.cgi?orf=13101.m01329) | [13101.t01094](http://rice.plantbiology.msu.edu/cgi-bin/sequence_display.cgi?orf=13101.m01329) | n/a | 981 | 380 | 34707.89 | 5.10 | 40-314 |  |
| *OsGELP12c* | [Os01g12320](http://rice.plantbiology.msu.edu/cgi-bin/ORF_infopage.cgi?orf=13101.m01330) | [13101.m01330](http://rice.plantbiology.msu.edu/cgi-bin/sequence_display.cgi?orf=13101.m01330) | [13101.t01094](http://rice.plantbiology.msu.edu/cgi-bin/sequence_display.cgi?orf=13101.m01330) | n/a | 450 | 150 | 16281.24 | 4.76 | 21-137 |  |
| *OsGELP13* | [Os01g12381](http://rice.plantbiology.msu.edu/cgi-bin/ORF_infopage.cgi?&orf=LOC_Os01g12381) | [13101.m01336](http://rice.plantbiology.msu.edu/cgi-bin/sequence_display.cgi?orf=13101.m01336) | [13101.t01100](http://rice.plantbiology.msu.edu/cgi-bin/sequence_display.cgi?orf=13101.m01336) | n/a | 915 | 305 | 32382.78 | 7.27 | 45-132 |  |
| *OsGELP14* | [Os01g22640](http://rice.plantbiology.msu.edu/cgi-bin/ORF_infopage.cgi?&orf=LOC_Os01g22640) | [13101.m02401](http://rice.plantbiology.msu.edu/cgi-bin/sequence_display.cgi?orf=13101.m02401) | [13101.t01978](http://rice.plantbiology.msu.edu/cgi-bin/sequence_display.cgi?orf=13101.m02401) | [AK120056](http://www.ncbi.nlm.nih.gov/entrez/viewer.fcgi?db=nucleotide&val=AK120056) | 1311 | 437 | 45706.59 | 7.73 | 101-411 |  |
| *OsGELP15a* | [Os01g22660.1](http://rice.plantbiology.msu.edu/cgi-bin/ORF_infopage.cgi?&orf=LOC_Os01g22660) | [13101.m02404](http://rice.plantbiology.msu.edu/cgi-bin/sequence_display.cgi?orf=13101.m02404) | [13101.t01980](http://rice.plantbiology.msu.edu/cgi-bin/sequence_display.cgi?orf=13101.m02404) | n/a | 1155 | 385 | 42092.81 | 8.46 | 56-366 |  |
| *OsGELP15b* | [Os01g22660.2](http://rice.plantbiology.msu.edu/cgi-bin/ORF_infopage.cgi?orf=13101.m02403) | [13101.m02403](http://rice.plantbiology.msu.edu/cgi-bin/sequence_display.cgi?orf=13101.m02403) | [13101.t01980](http://rice.plantbiology.msu.edu/cgi-bin/sequence_display.cgi?orf=13101.m02403) | [AK100480](http://www.ncbi.nlm.nih.gov/entrez/viewer.fcgi?db=nucleotide&val=AK100480) | 864 | 288 | 31613.84 | 6.50 | 8-269 |  |
| *OsGELP15c* | [Os01g22660](http://rice.plantbiology.msu.edu/cgi-bin/ORF_infopage.cgi?orf=13101.m02405).3 | [13101.m02405](http://rice.plantbiology.msu.edu/cgi-bin/sequence_display.cgi?orf=13101.m02405) | [13101.t01980](http://rice.plantbiology.msu.edu/cgi-bin/sequence_display.cgi?orf=13101.m02405) | n/a | 612 | 204 | 22395.34 | 5.76 | 1-185 |  |
| *OsGELP16a* | [Os01g22780.1](http://rice.plantbiology.msu.edu/cgi-bin/ORF_infopage.cgi?&orf=LOC_Os01g22780) | [13101.m02416](http://rice.plantbiology.msu.edu/cgi-bin/sequence_display.cgi?orf=13101.m02416) | [13101.t01991](http://rice.plantbiology.msu.edu/cgi-bin/sequence_display.cgi?orf=13101.m02416) | [AK100992](http://www.ncbi.nlm.nih.gov/entrez/viewer.fcgi?db=nucleotide&val=AK100992) | 1254 | 418 | 45652.14 | 9.39 | 52-307 |  |
| *OsGELP16b* | [Os01g22780.2](http://rice.plantbiology.msu.edu/cgi-bin/ORF_infopage.cgi?orf=13101.m02417) | [13101.m02417](http://rice.plantbiology.msu.edu/cgi-bin/sequence_display.cgi?orf=13101.m02417) | [13101.t01991](http://rice.plantbiology.msu.edu/cgi-bin/sequence_display.cgi?orf=13101.m02417) | n/a | 1254 | 385 | 40742.80 | 4.78 | 52-366 |  |
| *OsGELP17* | [Os01g42730](http://rice.plantbiology.msu.edu/cgi-bin/ORF_infopage.cgi?&orf=LOC_Os01g42730) | [13101.m04398](http://rice.plantbiology.msu.edu/cgi-bin/sequence_display.cgi?orf=13101.m04398) | [13101.t03709](http://rice.plantbiology.msu.edu/cgi-bin/sequence_display.cgi?orf=13101.m04398) | [AK243338.1](http://www.ncbi.nlm.nih.gov/sites/entrez?cmd=Retrieve&db=nucleotide&dopt=GenBank&list_uids=116012703) | 1227 | 409 | 44313.99 | 4.60 | 43-365 |  |
| *OsGELP18a* | [Os01g46080.1](http://rice.plantbiology.msu.edu/cgi-bin/ORF_infopage.cgi?&orf=13101.t03980) | [13101.m04730](http://rice.plantbiology.msu.edu/cgi-bin/sequence_display.cgi?orf=13101.m04730) | [13101.t03980](http://rice.plantbiology.msu.edu/cgi-bin/sequence_display.cgi?orf=13101.m04730) | n/a | 1221 | 407 | 43669.84 | 5.09 | 186-371 |  |
| *OsGELP18b* | [Os01g46080.2](http://rice.plantbiology.msu.edu/cgi-bin/ORF_infopage.cgi?orf=13101.m04731) | [13101.m04731](http://rice.plantbiology.msu.edu/cgi-bin/sequence_display.cgi?orf=13101.m04731) | [13101.t03980](http://rice.plantbiology.msu.edu/cgi-bin/sequence_display.cgi?orf=13101.m04731) | n/a | 1221 | 403 | 43371.46 | 4.97 | 186-368 |  |
| *OsGELP19* | [Os01g46090](http://rice.plantbiology.msu.edu/cgi-bin/ORF_infopage.cgi?&orf=LOC_Os01g46090) | [13101.m04732](http://rice.plantbiology.msu.edu/cgi-bin/sequence_display.cgi?orf=13101.m04732) | [13101.t03981](http://rice.plantbiology.msu.edu/cgi-bin/sequence_display.cgi?orf=13101.m04732) | n/a | 933 | 311 | 33665.28 | 4.19 | 86-275 |  |
| *OsGELP20a* | [Os01g46120.1](http://rice.plantbiology.msu.edu/cgi-bin/ORF_infopage.cgi?&orf=LOC_Os01g46120) | [13101.m04735](http://rice.plantbiology.msu.edu/cgi-bin/sequence_display.cgi?orf=13101.m04735) | [13101.t03984](http://rice.plantbiology.msu.edu/cgi-bin/sequence_display.cgi?orf=13101.m04735) | [AK068077](http://www.ncbi.nlm.nih.gov/entrez/viewer.fcgi?db=nucleotide&val=AK068077) | 1203 | 401 | 43381.58 | 4.71 | 180-365 |  |
| *OsGELP20b* | [Os01g46120.2](http://rice.plantbiology.msu.edu/cgi-bin/ORF_infopage.cgi?orf=13101.m04737) | [13101.m04737](http://rice.plantbiology.msu.edu/cgi-bin/sequence_display.cgi?orf=13101.m04737) | [13101.t03984](http://rice.plantbiology.msu.edu/cgi-bin/sequence_display.cgi?orf=13101.m04737) | n/a | 924 | 308 | 33618.50 | 4.50 | 87-272 |  |
| *OsGELP20c* | [Os01g46120.3](http://rice.plantbiology.msu.edu/cgi-bin/ORF_infopage.cgi?orf=13101.m04736) | [13101.m04736](http://rice.plantbiology.msu.edu/cgi-bin/sequence_display.cgi?orf=13101.m04736) | [13101.t03984](http://rice.plantbiology.msu.edu/cgi-bin/sequence_display.cgi?orf=13101.m04736) | n/a | 879 | 293 | 31940.89 | 6.86 | 38-125 |  |
| *OsGELP21a* | [Os01g46169.1](http://rice.plantbiology.msu.edu/cgi-bin/ORF_infopage.cgi?&orf=LOC_Os01g46169) | [13101.m04742](http://rice.plantbiology.msu.edu/cgi-bin/sequence_display.cgi?orf=13101.m04742) | [13101.t03987](http://rice.plantbiology.msu.edu/cgi-bin/sequence_display.cgi?orf=13101.m04742) | [AK064049](http://www.ncbi.nlm.nih.gov/entrez/viewer.fcgi?db=nucleotide&val=AK064049) | 888 | 296 | 32090.37 | 5.38 | 213-276 |  |
| *OsGELP21b* | [Os01g46169.2](http://rice.plantbiology.msu.edu/cgi-bin/ORF_infopage.cgi?orf=13101.m04741) | [13101.m04741](http://rice.plantbiology.msu.edu/cgi-bin/sequence_display.cgi?orf=13101.m04741) | [13101.t03987](http://rice.plantbiology.msu.edu/cgi-bin/sequence_display.cgi?orf=13101.m04741) | [AK102591](http://www.ncbi.nlm.nih.gov/entrez/viewer.fcgi?db=nucleotide&val=AK102591) | 783 | 261 | 28277.84 | 5.08 | 54-141 |  |
| *OsGELP21c* | [Os01g46169.3](http://rice.plantbiology.msu.edu/cgi-bin/ORF_infopage.cgi?orf=13101.m04743) | [13101.m04743](http://rice.plantbiology.msu.edu/cgi-bin/sequence_display.cgi?orf=13101.m04743) | [13101.t03987](http://rice.plantbiology.msu.edu/cgi-bin/sequence_display.cgi?orf=13101.m04743) | [AK062412](http://www.ncbi.nlm.nih.gov/entrez/viewer.fcgi?db=nucleotide&val=AK062412) | 669 | 223 | 24070.45 | 4.21 | 54-141 |  |
| *OsGELP22* | [Os01g46210](http://rice.plantbiology.msu.edu/cgi-bin/ORF_infopage.cgi?&orf=LOC_Os01g46210) | [13101.m04747](http://rice.plantbiology.msu.edu/cgi-bin/sequence_display.cgi?orf=13101.m04747) | [13101.t03989](http://rice.plantbiology.msu.edu/cgi-bin/sequence_display.cgi?orf=13101.m04747) | n/a | 1143 | 381 | 41346.91 | 7.29 | 48-368 |  |
| *OsGELP23* | [Os01g46220](http://rice.plantbiology.msu.edu/cgi-bin/ORF_infopage.cgi?&orf=LOC_Os01g46220) | [13101.m04748](http://rice.plantbiology.msu.edu/cgi-bin/sequence_display.cgi?orf=13101.m04748) | [13101.t03990](http://rice.plantbiology.msu.edu/cgi-bin/sequence_display.cgi?orf=13101.m04748) | [AK242032.1](http://www.ncbi.nlm.nih.gov/UniGene/seq.cgi?ORG=Os&SID=35192668) | 1113 | 371 | 40822.48 | 7.59 | 37-356 |  |
| *OsGELP24* | [Os01g52770](http://rice.plantbiology.msu.edu/cgi-bin/ORF_infopage.cgi?&orf=LOC_Os01g52770) | [13101.m05527](http://rice.plantbiology.msu.edu/cgi-bin/sequence_display.cgi?orf=13101.m05527) | [13101.t04624](http://rice.plantbiology.msu.edu/cgi-bin/sequence_display.cgi?orf=13101.m05527) | [AK070946](http://www.ncbi.nlm.nih.gov/entrez/viewer.fcgi?db=nucleotide&val=AK070946) | 1092 | 364 | 39083.81 | 4.57 | 32-343 |  |
| *OsGELP25* | [Os01g54470](http://rice.plantbiology.msu.edu/cgi-bin/ORF_infopage.cgi?&orf=LOC_Os01g54470) | [13101.m05723](http://rice.plantbiology.msu.edu/cgi-bin/sequence_display.cgi?orf=13101.m05723) | [13101.t04786](http://rice.plantbiology.msu.edu/cgi-bin/sequence_display.cgi?orf=13101.m05723) | [AK241862.1](http://www.ncbi.nlm.nih.gov/sites/entrez?cmd=Retrieve&db=nucleotide&dopt=GenBank&list_uids=116011227) | 1236 | 412 | 44971.00 | 9.08 | 79-391 |  |
| *OsGELP26* | [Os01g61200](http://rice.plantbiology.msu.edu/cgi-bin/ORF_infopage.cgi?&orf=LOC_Os01g61200) | [13101.m06513](http://rice.plantbiology.msu.edu/cgi-bin/sequence_display.cgi?orf=13101.m06513) | [13101.t05423](http://rice.plantbiology.msu.edu/cgi-bin/sequence_display.cgi?orf=13101.m06513) | n/a | 1053 | 351 | 39050.46 | 8.61 | 32-341 |  |
| *OsGELP27* | [Os01g61570](http://rice.plantbiology.msu.edu/cgi-bin/ORF_infopage.cgi?&orf=LOC_Os01g61570) | [13101.m06557](http://rice.plantbiology.msu.edu/cgi-bin/sequence_display.cgi?orf=13101.m06557) | [13101.t05460](http://rice.plantbiology.msu.edu/cgi-bin/sequence_display.cgi?orf=13101.m06557) | [AK064727](http://www.ncbi.nlm.nih.gov/entrez/viewer.fcgi?db=nucleotide&val=AK064727) | 1095 | 365 | 38499.06 | 7.13 | 44-354 |  |
| *OsGELP28* | [Os01g72850](http://rice.plantbiology.msu.edu/cgi-bin/ORF_infopage.cgi?&orf=LOC_Os01g72850) | [13101.m07944](http://rice.plantbiology.msu.edu/cgi-bin/sequence_display.cgi?orf=13101.m07944) | [13101.t06500](http://rice.plantbiology.msu.edu/cgi-bin/sequence_display.cgi?orf=13101.m07944) | n/a | 627 | 209 | 22988.26 | 8.39 | 84-188 |  |
| *OsGELP29* | [Os02g01140](http://rice.plantbiology.msu.edu/cgi-bin/ORF_infopage.cgi?&orf=LOC_Os02g01140) | [13102.m00020](http://rice.plantbiology.msu.edu/cgi-bin/sequence_display.cgi?orf=13102.m00020) | [13102.t00014](http://rice.plantbiology.msu.edu/cgi-bin/sequence_display.cgi?orf=13102.m00020) | [AK067036](http://www.ncbi.nlm.nih.gov/entrez/viewer.fcgi?db=nucleotide&val=AK067036) | 1080 | 360 | 38931.9 | 7.76 | 35-348 |  |
| *OsGELP30* | [Os02g01980](http://rice.plantbiology.msu.edu/cgi-bin/ORF_infopage.cgi?&orf=LOC_Os02g01980) | [13102.m00127](http://rice.plantbiology.msu.edu/cgi-bin/sequence_display.cgi?orf=13102.m00127) | [13102.t00102](http://rice.plantbiology.msu.edu/cgi-bin/sequence_display.cgi?orf=13102.m00127) | [AK106946](http://www.ncbi.nlm.nih.gov/entrez/viewer.fcgi?db=nucleotide&val=AK106946) | 1362 | 454 | 49540 | 8.63 | 135-443 |  |
| *OsGELP31* | [Os02g09610](http://rice.plantbiology.msu.edu/cgi-bin/ORF_infopage.cgi?&orf=LOC_Os02g09610) | [13102.m01053](http://rice.plantbiology.msu.edu/cgi-bin/sequence_display.cgi?orf=13102.m01053) | [13102.t00820](http://rice.plantbiology.msu.edu/cgi-bin/sequence_display.cgi?orf=13102.m01053) | [AK108567](http://www.ncbi.nlm.nih.gov/entrez/viewer.fcgi?db=nucleotide&val=AK108567) | 1134 | 378 | 39229.37 | 4.81 | 39-368 |  |
| *OsGELP32* | [Os02g09620](http://rice.plantbiology.msu.edu/cgi-bin/ORF_infopage.cgi?&orf=LOC_Os02g09620) | [13102.m01054](http://rice.plantbiology.msu.edu/cgi-bin/sequence_display.cgi?orf=13102.m01054) | [13102.t00821](http://rice.plantbiology.msu.edu/cgi-bin/sequence_display.cgi?orf=13102.m01054) | [AK060691](http://www.ncbi.nlm.nih.gov/entrez/viewer.fcgi?db=nucleotide&val=AK060691) | 1095 | 365 | 39307.9 | 7.1 | 37-354 |  |
| *OsGELP33a* | [Os02g15230](http://rice.plantbiology.msu.edu/cgi-bin/ORF_infopage.cgi?&orf=LOC_Os02g15230).1 | [13102.m01699](http://rice.plantbiology.msu.edu/cgi-bin/sequence_display.cgi?orf=13102.m01699) | [13102.t01330](http://rice.plantbiology.msu.edu/cgi-bin/sequence_display.cgi?orf=13102.m01699) | n/a | 1149 | 383 | 41310 | 6.8 | 39-368 | *GER1* |
| *OsGELP33b* | [Os02g15230.2](http://rice.plantbiology.msu.edu/cgi-bin/ORF_infopage.cgi?orf=13102.m01698) | [13102.m01698](http://rice.plantbiology.msu.edu/cgi-bin/sequence_display.cgi?orf=13102.m01698) | [13102.t01330](http://rice.plantbiology.msu.edu/cgi-bin/sequence_display.cgi?orf=13102.m01698) | [AK070671](http://www.ncbi.nlm.nih.gov/entrez/viewer.fcgi?db=nucleotide&val=AK070671) | 735 | 245 | 25177.7 | 11.9 | 39-88 |  |
| *OsGELP33c* | [Os02g15230.3](http://rice.plantbiology.msu.edu/cgi-bin/ORF_infopage.cgi?orf=13102.m01700) | [13102.m01700](http://rice.plantbiology.msu.edu/cgi-bin/sequence_display.cgi?orf=13102.m01700) | [13102.t01330](http://rice.plantbiology.msu.edu/cgi-bin/sequence_display.cgi?orf=13102.m01700) | n/a | 654 | 218 | 22995.2 | 4.98 | 39-154 |  |
| *OsGELP34* | [Os02g18870](http://rice.plantbiology.msu.edu/cgi-bin/ORF_infopage.cgi?&orf=LOC_Os02g18870) | [13102.m02115](http://rice.plantbiology.msu.edu/cgi-bin/sequence_display.cgi?orf=13102.m02115) | [13102.t01694](http://www.tigr.org/tigr-scripts/euk_manatee/shared/seq_display.cgi?db=osa1r5&orf=12002.m07130) | n/a | 1200 | 400 | 44141.4 | 5.62 | 56-378 |  |
| *OsGELP35* | [Os02g18954](http://rice.plantbiology.msu.edu/cgi-bin/ORF_infopage.cgi?&orf=LOC_Os02g18954) | [13102.m02123](http://rice.plantbiology.msu.edu/cgi-bin/sequence_display.cgi?orf=13102.m02123) | 3102.t01702 | [AK101883](http://www.ncbi.nlm.nih.gov/entrez/viewer.fcgi?db=nucleotide&val=AK101883) | 1128 | 376 | 39806 | 8.23 | 36-354 |  |
| *OsGELP36* | [Os02g18990](http://rice.plantbiology.msu.edu/cgi-bin/ORF_infopage.cgi?&orf=LOC_Os02g18990) | [13102.m02126](http://rice.plantbiology.msu.edu/cgi-bin/sequence_display.cgi?orf=13102.m02126) | 13102.t01705 | n/a | 1182 | 394 | 42096.20 | 4.74 | 44-371 |  |
| *OsGELP37* | [Os02g19040](http://rice.plantbiology.msu.edu/cgi-bin/ORF_infopage.cgi?&orf=LOC_Os02g19040) | [13102.m02131](http://rice.plantbiology.msu.edu/cgi-bin/sequence_display.cgi?orf=13102.m02131) | 13102.t01710 | [AK106688](http://www.ncbi.nlm.nih.gov/entrez/viewer.fcgi?db=nucleotide&val=AK106688) | 1230 | 410 | 44988.3 | 9.06 | 84-389 |  |
| *OsGELP38* | [Os02g39170](http://rice.plantbiology.msu.edu/cgi-bin/ORF_infopage.cgi?&orf=LOC_Os02g39170) | [13102.m04329](http://rice.plantbiology.msu.edu/cgi-bin/sequence_display.cgi?orf=13102.m04329) | 13102.t03578 | n/a | 1221 | 407 | 42889 | 8.98 | 66-387 |  |
| *OsGELP39* | [Os02g39590](http://rice.plantbiology.msu.edu/cgi-bin/ORF_infopage.cgi?&orf=LOC_Os02g39590) | [13102.m04389](http://rice.plantbiology.msu.edu/cgi-bin/sequence_display.cgi?orf=13102.m04389) | 13102.t03621 | n/a | 1212 | 404 | 42450.3 | 6.5 | 45-387 |  |
| *OsGELP40* | [Os02g40440](http://rice.plantbiology.msu.edu/cgi-bin/ORF_infopage.cgi?&orf=LOC_Os02g40440) | [13102.m04506](http://rice.plantbiology.msu.edu/cgi-bin/sequence_display.cgi?orf=13102.m04506) | 13102.t03705 | [AK108108](http://www.ncbi.nlm.nih.gov/entrez/viewer.fcgi?db=nucleotide&val=AK108108) | 1104 | 368 | 40154.7 | 5.96 | 29-341 |  |
| *OsGELP41* | [Os02g44850](http://rice.plantbiology.msu.edu/cgi-bin/ORF_infopage.cgi?&orf=LOC_Os02g44850) | [13102.m05025](http://rice.plantbiology.msu.edu/cgi-bin/sequence_display.cgi?orf=13102.m05025) | 13102.t04108 | n/a | 1092 | 364 | 38880.8 | 7.7 | 29-344 |  |
| *OsGELP42* | [Os02g44860](http://rice.plantbiology.msu.edu/cgi-bin/ORF_infopage.cgi?&orf=LOC_Os02g44860) | [13102.m05026](http://rice.plantbiology.msu.edu/cgi-bin/sequence_display.cgi?orf=13102.m05026) | 13102.t04109 | [AK059531](http://www.ncbi.nlm.nih.gov/entrez/viewer.fcgi?db=nucleotide&val=AK059531)  AK103585 | 1089 | 363 | 38583.9 | 9.24 | 29-341 |  |
| *OsGELP43* | [Os02g50000](http://rice.plantbiology.msu.edu/cgi-bin/ORF_infopage.cgi?&orf=LOC_Os02g50000) | [13102.m05680](http://rice.plantbiology.msu.edu/cgi-bin/sequence_display.cgi?orf=13102.m05680) | 13102.t04627 | n/a | 969 | 323 | 33775.5 | 7.4 | 153-293 |  |
| *OsGELP44a* | [Os02g50690](http://rice.plantbiology.msu.edu/cgi-bin/ORF_infopage.cgi?&orf=LOC_Os02g50690).1 | [13102.m05752](http://rice.plantbiology.msu.edu/cgi-bin/sequence_display.cgi?orf=13102.m05752) | 13102.t04696 | [AK070261](http://www.ncbi.nlm.nih.gov/entrez/viewer.fcgi?db=nucleotide&val=AK070261) | 1152 | 384 | 40026.2 | 9.94 | 40-362 |  |
| *OsGELP44b* | [Os02g50690.2](http://rice.plantbiology.msu.edu/cgi-bin/ORF_infopage.cgi?orf=13102.m12338) | [13102.m12338](http://rice.plantbiology.msu.edu/cgi-bin/sequence_display.cgi?orf=13102.m12338) | 13102.t04696 | [AK111360](http://www.ncbi.nlm.nih.gov/entrez/viewer.fcgi?db=nucleotide&val=AK111360) | 813 | 270 | 28185.9 | 9.67 | **16-249** |  |
| *OsGELP45a* | [Os02g57110](http://rice.plantbiology.msu.edu/cgi-bin/ORF_infopage.cgi?&orf=LOC_Os02g57110) | [13102.m06569](http://rice.plantbiology.msu.edu/cgi-bin/sequence_display.cgi?orf=13102.m06569) | 13102.t05337 | [AK060243](http://www.ncbi.nlm.nih.gov/entrez/viewer.fcgi?db=nucleotide&val=AK060243) | 1110 | 370 | 40293.4 | 5.35 | 30-342 |  |
| *OsGELP45b* | [Os02g57110.2](http://rice.plantbiology.msu.edu/cgi-bin/ORF_infopage.cgi?orf=13102.m12344) | [13102.m12344](http://rice.plantbiology.msu.edu/cgi-bin/sequence_display.cgi?orf=13102.m12344) | 13102.t05337 | n/a | 879 | 292 | 32185.4 | 5.0 | **14**-**265** |  |
| *OsGELP46a* | [Os03g19670.1](http://rice.plantbiology.msu.edu/cgi-bin/ORF_infopage.cgi?&orf=LOC_Os03g19670) | [13103.m02358](http://rice.plantbiology.msu.edu/cgi-bin/sequence_display.cgi?orf=13103.m02358) | 13103.t01756 | [AK060625](http://www.ncbi.nlm.nih.gov/entrez/viewer.fcgi?db=nucleotide&val=AK060625) | 1104 | 368 | 39871.52 | 6.66 | 34-348 |  |
| *OsGELP46b* | [Os03g19670.2](http://rice.plantbiology.msu.edu/cgi-bin/ORF_infopage.cgi?orf=13103.m02359) | [13103.m02359](http://rice.plantbiology.msu.edu/cgi-bin/sequence_display.cgi?orf=13103.m02359) | 13103.t01756 | n/a | 846 | 282 | 30733.94 | 6.90 | 5-262 |  |
| *OsGELP47* | [Os03g25000](http://rice.plantbiology.msu.edu/cgi-bin/ORF_infopage.cgi?&orf=LOC_Os03g25000) | [13103.m02947](http://rice.plantbiology.msu.edu/cgi-bin/sequence_display.cgi?orf=13103.m02947) | 13103.t02222 | n/a | 831 | 277 | 31272.24 | 6.51 | 7-263 |  |
| *OsGELP48* | [Os03g25010](http://rice.plantbiology.msu.edu/cgi-bin/ORF_infopage.cgi?&orf=LOC_Os03g25010) | [13103.m02948](http://rice.plantbiology.msu.edu/cgi-bin/sequence_display.cgi?orf=13103.m02948) | [13103.t02223](http://www.tigr.org/tigr-scripts/euk_manatee/shared/seq_display.cgi?db=osa1r5&orf=12003.m07844) | n/a | 1020 | 340 | 37243.52 | 8.22 | 55-291 |  |
| *OsGELP49* | [Os03g25030](http://rice.plantbiology.msu.edu/cgi-bin/ORF_infopage.cgi?&orf=LOC_Os03g25030) | [13103.m02950](http://rice.plantbiology.msu.edu/cgi-bin/sequence_display.cgi?orf=13103.m02950) | 3103.t02225 | [AK063672](http://www.ncbi.nlm.nih.gov/entrez/viewer.fcgi?db=nucleotide&val=AK063672) | 1164 | 388 | 42553.59 | 7.31 | 47-373 |  |
| *OsGELP50a* | [Os03g25040](http://rice.plantbiology.msu.edu/cgi-bin/ORF_infopage.cgi?&orf=LOC_Os03g25040).1 | [13103.m02952](http://rice.plantbiology.msu.edu/cgi-bin/sequence_display.cgi?orf=13103.m02952) | 13103.t02226 | n/a | 1179 | 393 | 43174.5 | 8.15 | 51-376 |  |
| *OsGELP50b* | [Os03g25040.2](http://rice.plantbiology.msu.edu/cgi-bin/ORF_infopage.cgi?orf=13103.m02951) | [13103.m02951](http://rice.plantbiology.msu.edu/cgi-bin/sequence_display.cgi?orf=13103.m02951) | 13103.t02226 | [AK069388](http://www.ncbi.nlm.nih.gov/entrez/viewer.fcgi?db=nucleotide&val=AK069388) | 621 | 207 | 22989.40 | 8.28 | 3-190 |  |
| *OsGELP51* | [Os03g38390](http://rice.plantbiology.msu.edu/cgi-bin/ORF_infopage.cgi?&orf=LOC_Os03g38390) | [13103.m04166](http://rice.plantbiology.msu.edu/cgi-bin/sequence_display.cgi?orf=13103.m04166) | 13103.t03300 | [AK070980](http://www.ncbi.nlm.nih.gov/entrez/viewer.fcgi?db=nucleotide&val=AK070980) | 1140 | 380 | 41093.04 | 6.94 | 48-360 |  |
| *OsGELP52* | [Os03g38470](http://rice.plantbiology.msu.edu/cgi-bin/ORF_infopage.cgi?&orf=LOC_Os03g38470) | [13103.m04172](http://rice.plantbiology.msu.edu/cgi-bin/sequence_display.cgi?orf=13103.m04172) | 13103.t03306 | n/a | 1104 | 368 | 39820.2 | 7.58 | 41-348 |  |
| *OsGELP53* | [Os03g47940](http://rice.plantbiology.msu.edu/cgi-bin/ORF_infopage.cgi?&orf=LOC_Os03g47940) | [13103.m05170](http://rice.plantbiology.msu.edu/cgi-bin/sequence_display.cgi?orf=13103.m05170) | 13103.t04167 | [AK099266](http://www.ncbi.nlm.nih.gov/entrez/viewer.fcgi?db=nucleotide&val=AK099266) | 1074 | 358 | 37411.2 | 5.31 | 31-348 |  |
| *OsGELP54* | [Os03g62740](http://rice.plantbiology.msu.edu/cgi-bin/ORF_infopage.cgi?&orf=LOC_Os03g62740) | [13103.m06945](http://rice.plantbiology.msu.edu/cgi-bin/sequence_display.cgi?orf=13103.m06945) | 13103.t05514 | [AK059168.1](http://www.ncbi.nlm.nih.gov/UniGene/seq.cgi?ORG=Os&SID=16418835) | 1104 | 368 | 39934.4 | 8.21 | 27-311 |  |
| *OsGELP55a* | [Os03g64170.1](http://rice.plantbiology.msu.edu/cgi-bin/ORF_infopage.cgi?&orf=LOC_Os03g64170) | [13103.m07121](http://rice.plantbiology.msu.edu/cgi-bin/sequence_display.cgi?orf=13103.m07121) | 13103.t05643 | [AK060992](http://www.ncbi.nlm.nih.gov/entrez/viewer.fcgi?db=nucleotide&val=AK060992) | 1098 | 366 | 39806.48 | 5.21 | 44-355 |  |
| *OsGELP55b* | [Os03g64170.2](http://rice.plantbiology.msu.edu/cgi-bin/ORF_infopage.cgi?orf=13103.m07120) | [13103.m07120](http://rice.plantbiology.msu.edu/cgi-bin/sequence_display.cgi?orf=13103.m07120) | 13103.t05643 | [AK120038](http://www.ncbi.nlm.nih.gov/entrez/viewer.fcgi?db=nucleotide&val=AK120038) | 831 | 277 | 30548.75 | 4.68 | 6-266 |  |
| *OsGELP56* | [Os04g42860](http://rice.plantbiology.msu.edu/cgi-bin/ORF_infopage.cgi?&orf=LOC_Os04g42860) | [13104.m04241](http://rice.plantbiology.msu.edu/cgi-bin/sequence_display.cgi?orf=13104.m04241) | 13104.t03817 | [AK121383](http://www.ncbi.nlm.nih.gov/entrez/viewer.fcgi?db=nucleotide&val=AK121383) | 1107 | 369 | 39958.34 | 4.96 | 32-344 |  |
| *OsGELP57* | [Os04g47390](http://rice.plantbiology.msu.edu/cgi-bin/ORF_infopage.cgi?&orf=LOC_Os04g47390) | [13104.m04799](http://rice.plantbiology.msu.edu/cgi-bin/sequence_display.cgi?orf=13104.m04799) | 13104.t04254 | n/a | 1104 | 368 | 39239.4 | 6.38 | 33-346 |  |
| *OsGELP58a* | [Os04g48800.1](http://rice.plantbiology.msu.edu/cgi-bin/ORF_infopage.cgi?&orf=LOC_Os04g48800) | [13104.m04973](http://rice.plantbiology.msu.edu/cgi-bin/sequence_display.cgi?orf=13104.m04973) | 13104.t04390 | n/a | 969 | 323 | 35666.9 | 7.77 | 44-300 |  |
| *OsGELP58b* | [Os04g48800.2](http://rice.plantbiology.msu.edu/cgi-bin/ORF_infopage.cgi?orf=13104.m04972) | [13104.m04972](http://rice.plantbiology.msu.edu/cgi-bin/sequence_display.cgi?orf=13104.m04972) | 13104.t04390 | [AK120597](http://www.ncbi.nlm.nih.gov/entrez/viewer.fcgi?db=nucleotide&val=AK120597) | 969 | 269 | 29940.2 | 8.07 | 5-246 |  |
| *OsGELP58c* | [Os04g48800.3](http://rice.plantbiology.msu.edu/cgi-bin/ORF_infopage.cgi?orf=13104.m04974) | [13104.m04974](http://rice.plantbiology.msu.edu/cgi-bin/sequence_display.cgi?orf=13104.m04974) | 13104.t04390 | n/a | 807 | 269 | 29940.2 | 8.07 | 5-246 |  |
| *OsGELP58d* | [Os04g48800.4](http://rice.plantbiology.msu.edu/cgi-bin/ORF_infopage.cgi?orf=13104.m04975) | [13104.m04975](http://rice.plantbiology.msu.edu/cgi-bin/sequence_display.cgi?orf=13104.m04975) | 13104.t04390 | n/a | 693 | 231 | 25788.7 | 8.32 | 5-191 |  |
| *OsGELP59* | [Os04g55660](http://rice.plantbiology.msu.edu/cgi-bin/ORF_infopage.cgi?&orf=LOC_Os04g55660) | [13104.m05775](http://rice.plantbiology.msu.edu/cgi-bin/sequence_display.cgi?orf=13104.m05775) | 13104.t05043 | [AK109786](http://www.ncbi.nlm.nih.gov/entrez/viewer.fcgi?db=nucleotide&val=AK109786) | 1068 | 356 | 39679.6 | 8.64 | 318-344 |  |
| *OsGELP60* | [Os05g04240](http://rice.plantbiology.msu.edu/cgi-bin/ORF_infopage.cgi?&orf=LOC_Os05g04240) | [13105.m00423](http://rice.plantbiology.msu.edu/cgi-bin/sequence_display.cgi?orf=13105.m00423) | 13105.t00321 | [AK289158](http://www.ncbi.nlm.nih.gov/nuccore/156765773?report=genbank) | 1098 | 366 | 39402.9 | 8.20 | 31-348 |  |
| *OsGELP61* | [Os05g06710](http://rice.plantbiology.msu.edu/cgi-bin/ORF_infopage.cgi?&orf=LOC_Os05g06710) | [13105.m00724](http://rice.plantbiology.msu.edu/cgi-bin/sequence_display.cgi?orf=13105.m00724) | 13105.t00566 | [AK067837](http://www.ncbi.nlm.nih.gov/entrez/viewer.fcgi?db=nucleotide&val=AK067837) | 1296 | 432 | 45678.7 | 7.54 | 101-405 |  |
| *OsGELP62a* | [Os05g06720.1](http://rice.plantbiology.msu.edu/cgi-bin/ORF_infopage.cgi?orf=13105.m00727) | [13105.m00727](http://rice.plantbiology.msu.edu/cgi-bin/sequence_display.cgi?orf=13105.m00727) | 13105.t00567 | n/a | 636 | 212 | 23513.2 | 8.87 | 2-184 |  |
| *OsGELP62b* | [Os05g06720.2](http://rice.plantbiology.msu.edu/cgi-bin/ORF_infopage.cgi?orf=13105.m00726) | [13105.m00726](http://rice.plantbiology.msu.edu/cgi-bin/sequence_display.cgi?orf=13105.m00726) | 13105.t00567 | n/a | 636 | 212 | 23513.20 | 8.87 | 2-184 |  |
| *OsGELP62c* | [Os05g06720.3](http://rice.plantbiology.msu.edu/cgi-bin/ORF_infopage.cgi?orf=13105.m00725) | [13105.m00725](http://rice.plantbiology.msu.edu/cgi-bin/sequence_display.cgi?orf=13105.m00725) | 13105.t00567 | [AK070904](http://www.ncbi.nlm.nih.gov/entrez/viewer.fcgi?db=nucleotide&val=AK070904) | 306 | 102 | 11149.90 | 9.57 | 2-74 |  |
| *OsGELP63* | [Os05g11910](http://rice.plantbiology.msu.edu/cgi-bin/ORF_infopage.cgi?&orf=LOC_Os05g11910) | [13105.m01290](http://rice.plantbiology.msu.edu/cgi-bin/sequence_display.cgi?orf=13105.m01290) | 13105.t01034 | [AK059511](http://www.ncbi.nlm.nih.gov/entrez/viewer.fcgi?db=nucleotide&val=AK059511) | 1086 | 362 | 38958.5 | 8.62 | 163-349 |  |
| *OsGELP64a* | [Os05g11950](http://rice.plantbiology.msu.edu/cgi-bin/ORF_infopage.cgi?&orf=LOC_Os05g11950).1 | [13105.m01294](http://rice.plantbiology.msu.edu/cgi-bin/sequence_display.cgi?orf=13105.m01294) | 13105.t01038 | [AK122049](http://www.ncbi.nlm.nih.gov/entrez/viewer.fcgi?db=nucleotide&val=AK122049) | 1113 | 371 | 40996.7 | 7.11 | 33-350 |  |
| *OsGELP64b* | [Os05g11950.2](http://rice.plantbiology.msu.edu/cgi-bin/ORF_infopage.cgi?orf=13105.m01295) | [13105.m01295](http://rice.plantbiology.msu.edu/cgi-bin/sequence_display.cgi?orf=13105.m01295) | 13105.t01038 | n/a | 948 | 316 | 35081.9 | 6.15 | 21-295 |  |
| *OsGELP65* | [Os05g11970](http://rice.plantbiology.msu.edu/cgi-bin/ORF_infopage.cgi?&orf=LOC_Os05g11970) | [13105.m01297](http://rice.plantbiology.msu.edu/cgi-bin/sequence_display.cgi?orf=13105.m01297) | 13105.t01040 | n/a | 1143 | 381 | 40480.1 | 9.36 | 34-364 |  |
| *OsGELP66* | [Os05g33270](http://rice.plantbiology.msu.edu/cgi-bin/ORF_infopage.cgi?&orf=LOC_Os05g33270) | [13105.m03438](http://rice.plantbiology.msu.edu/cgi-bin/sequence_display.cgi?orf=13105.m03438) | 13105.t02948 | [AK240941.1](http://www.ncbi.nlm.nih.gov/UniGene/seq.cgi?ORG=Os&SID=35188358) | 1167 | 389 | 42546.2 | 7.35 | 39-366 |  |
| *OsGELP67a* | [Os05g34700.1](http://rice.plantbiology.msu.edu/cgi-bin/ORF_infopage.cgi?&orf=LOC_Os05g34700) | [13105.m03613](http://rice.plantbiology.msu.edu/cgi-bin/sequence_display.cgi?orf=13105.m03613) | 13105.t03092 | [AK100958](http://www.ncbi.nlm.nih.gov/entrez/viewer.fcgi?db=nucleotide&val=AK100958) | 1182 | 394 | 42229.0 | 8.72 | 62-372 |  |
| *OsGELP67b* | [Os05g34700.2](http://rice.plantbiology.msu.edu/cgi-bin/ORF_infopage.cgi?orf=13105.m03614) | [13105.m03614](http://rice.plantbiology.msu.edu/cgi-bin/sequence_display.cgi?orf=13105.m03614) | 13105.t03092 | n/a | 963 | 321 | 34040.6 | 8.58 | 62-310 |  |
| *OsGELP68* | [Os05g39220](http://rice.plantbiology.msu.edu/cgi-bin/ORF_infopage.cgi?&orf=LOC_Os05g39220) | [13105.m04129](http://rice.plantbiology.msu.edu/cgi-bin/sequence_display.cgi?orf=13105.m04129) | 13105.t03496 | [AK064681](http://www.ncbi.nlm.nih.gov/entrez/viewer.fcgi?db=nucleotide&val=AK064681) | 1083 | 361 | 37482.5 | 5.60 | 43-350 |  |
| *OsGELP69* | [Os05g43090](http://rice.plantbiology.msu.edu/cgi-bin/ORF_infopage.cgi?&orf=LOC_Os05g43090) | [13105.m04535](http://rice.plantbiology.msu.edu/cgi-bin/sequence_display.cgi?orf=13105.m04535) | 13105.t03837 | n/a | 855 | 285 | 31835.7 | 8.77 | 28-277 |  |
| *OsGELP70* | [Os05g43100](http://rice.plantbiology.msu.edu/cgi-bin/ORF_infopage.cgi?&orf=LOC_Os05g43100) | [13105.m04536](http://rice.plantbiology.msu.edu/cgi-bin/sequence_display.cgi?orf=13105.m04536) | 13105.t03838 | n/a | 1116 | 372 | 40229 | 5.56 | 169-355 |  |
| *OsGELP71* | [Os05g43110](http://rice.plantbiology.msu.edu/cgi-bin/ORF_infopage.cgi?&orf=LOC_Os05g43110) | [13105.m04537](http://rice.plantbiology.msu.edu/cgi-bin/sequence_display.cgi?orf=13105.m04537) | 13105.t03839 | n/a | 966 | 322 | 36063.6 | 10.02 | 186-305 |  |
| *OsGELP72* | [Os05g43120](http://rice.plantbiology.msu.edu/cgi-bin/ORF_infopage.cgi?&orf=LOC_Os05g43120) | [13105.m04538](http://rice.plantbiology.msu.edu/cgi-bin/sequence_display.cgi?orf=13105.m04538) | 13105.t03840 | [AK241401.1](http://www.ncbi.nlm.nih.gov/UniGene/seq.cgi?ORG=Os&SID=35193957) | 1128 | 376 | 40950.1 | 7.24 | 171-357 |  |
| *OsGELP73* | [Os05g44200](http://rice.plantbiology.msu.edu/cgi-bin/ORF_infopage.cgi?&orf=LOC_Os05g44200) | [13105.m04665](http://rice.plantbiology.msu.edu/cgi-bin/sequence_display.cgi?orf=13105.m04665) | 13105.t03946 | [AK061147](http://www.ncbi.nlm.nih.gov/entrez/viewer.fcgi?db=nucleotide&val=AK061147) | 1098 | 366 | 38955.7 | 5.27 | 33-345 |  |
| *OsGELP74* | [Os06g05550](http://rice.plantbiology.msu.edu/cgi-bin/ORF_infopage.cgi?&orf=LOC_Os06g05550) | [13106.m00542](http://rice.plantbiology.msu.edu/cgi-bin/sequence_display.cgi?orf=13106.m00542) | 13106.t00440 | [AK060175](http://www.ncbi.nlm.nih.gov/entrez/viewer.fcgi?db=nucleotide&val=AK060175) | 1056 | 352 | 37624.6 | 5.31 | 29-342 |  |
| *OsGELP75a* | [Os06g05630.1](http://rice.plantbiology.msu.edu/cgi-bin/ORF_infopage.cgi?&orf=LOC_Os06g05630) | [13106.m00551](http://rice.plantbiology.msu.edu/cgi-bin/sequence_display.cgi?orf=13106.m00551) | 13106.t00448 | n/a | 1083 | 361 | 38654.8 | 8.23 | 37-351 |  |
| *OsGELP75b* | [Os06g05630.2](http://rice.plantbiology.msu.edu/cgi-bin/ORF_infopage.cgi?orf=13106.m00553) | [13106.m00553](http://rice.plantbiology.msu.edu/cgi-bin/sequence_display.cgi?orf=13106.m00553) | [113106.t0044](http://www.tigr.org/tigr-scripts/euk_manatee/shared/seq_display.cgi?db=osa1r5&orf=12006.m71414) | n/a | 909 | 303 | 32546.9 | 9.08 | 37-295 |  |
| *OsGELP75c* | [Os06g05630.3](http://rice.plantbiology.msu.edu/cgi-bin/ORF_infopage.cgi?orf=13106.m00552) | [13106.m00552](http://rice.plantbiology.msu.edu/cgi-bin/sequence_display.cgi?orf=13106.m00552) | 13106.t00448 | [AK067418](http://www.ncbi.nlm.nih.gov/entrez/viewer.fcgi?db=nucleotide&val=AK067418) | 597 | 199 | 20795.7 | 9.22 | 54-189 |  |
| *OsGELP76* | [Os06g06230](http://rice.plantbiology.msu.edu/cgi-bin/ORF_infopage.cgi?&orf=LOC_Os06g06230) | [13106.m00638](http://rice.plantbiology.msu.edu/cgi-bin/sequence_display.cgi?orf=13106.m00638) | 13106.t00509 | n/a | 570 | 190 | 20039.93 | 4.97 | 14-154 |  |
| *OsGELP77a* | [Os06g06250.1](http://rice.plantbiology.msu.edu/cgi-bin/ORF_infopage.cgi?orf=13106.m00640) | [13106.m00640](../../../RICE%20lipase%202008/MOtif%20distrib%20MEME%20SALAD%20poster%2015.11http:/rice.plantbiology.msu.edu/cgi-bin/sequence_display.cgi%3Forf=13106.m00640) | 13106.t00511 | n/a | 864 | 287 | 30939.9 | 7.89 | **90**-**275** |  |
| *OsGELP77b* | [Os06g06250](http://rice.plantbiology.msu.edu/cgi-bin/ORF_infopage.cgi?&orf=LOC_Os06g06250).2 | [13106.m10511](http://rice.plantbiology.msu.edu/cgi-bin/sequence_display.cgi?orf=13106.m10511) | 13106.t00511 | [AK121780](http://www.ncbi.nlm.nih.gov/nuccore/37991403)  [AK104883](http://www.ncbi.nlm.nih.gov/nuccore/32990092)  [AK104394](http://www.ncbi.nlm.nih.gov/nuccore/32989603)  [AK061497](http://www.ncbi.nlm.nih.gov/nuccore/32971515) | 1083 | 361 | 38478.6 | 7.98 | 163-348 |  |
| *OsGELP78* | [Os06g06260](http://rice.plantbiology.msu.edu/cgi-bin/ORF_infopage.cgi?&orf=LOC_Os06g06260) | [13106.m00641](http://rice.plantbiology.msu.edu/cgi-bin/sequence_display.cgi?orf=13106.m00641) | 13106.t00512 | [AK107226](http://www.ncbi.nlm.nih.gov/entrez/viewer.fcgi?db=nucleotide&val=AK107226) | 1215 | 405 | 43053.6 | 7.97 | 198-391 |  |
| *OsGELP79* | [Os06g06290](http://rice.plantbiology.msu.edu/cgi-bin/ORF_infopage.cgi?&orf=LOC_Os06g06290) | [13106.m00645](http://rice.plantbiology.msu.edu/cgi-bin/sequence_display.cgi?orf=13106.m00645) | 13106.t00515 | [AK066113](http://www.ncbi.nlm.nih.gov/entrez/viewer.fcgi?db=nucleotide&val=AK066113) | 1026 | 341 | 39477.4 | 7.91 | 34-355 |  |
| *OsGELP80* | [Os06g06520](http://rice.plantbiology.msu.edu/cgi-bin/ORF_infopage.cgi?&orf=LOC_Os06g06520) | [13106.m00673](http://rice.plantbiology.msu.edu/cgi-bin/sequence_display.cgi?orf=13106.m00673) | 13106.t00537 | [AK073443](http://www.ncbi.nlm.nih.gov/entrez/viewer.fcgi?db=nucleotide&val=AK073443) | 1140 | 380 | 40957.5 | 8.24 | 32-357 |  |
| *OsGELP81* | [Os06g12410](http://rice.plantbiology.msu.edu/cgi-bin/ORF_infopage.cgi?&orf=LOC_Os06g12410) | [13106.m01356](http://rice.plantbiology.msu.edu/cgi-bin/sequence_display.cgi?orf=13106.m01356) | 13106.t01116 | n/a | 1041 | 363 | 39918.6 | 8.60 | 19-330 |  |
| *OsGELP82* | [Os06g14630](http://rice.plantbiology.msu.edu/cgi-bin/ORF_infopage.cgi?&orf=LOC_Os06g14630) | [13106.m01624](http://rice.plantbiology.msu.edu/cgi-bin/sequence_display.cgi?orf=13106.m01624) | 13106.t01337 | [AK108686](http://www.ncbi.nlm.nih.gov/entrez/viewer.fcgi?db=nucleotide&val=AK108686) | 1173 | 391 | 41241.7 | 4.48 | 33-343 |  |
| *OsGELP83a* | [Os06g24404.1](http://rice.plantbiology.msu.edu/cgi-bin/ORF_infopage.cgi?&orf=LOC_Os06g24404) | [13106.m02619](http://rice.plantbiology.msu.edu/cgi-bin/sequence_display.cgi?orf=13106.m02619) | 13106.t02247 | [AK067876](http://www.ncbi.nlm.nih.gov/entrez/viewer.fcgi?db=nucleotide&val=AK067876) | 1137 | 379 | 41784.6 | 5.41 | 61-369 |  |
| *OsGELP83b* | [Os06g24404.2](http://rice.plantbiology.msu.edu/cgi-bin/ORF_infopage.cgi?orf=13106.m02620) | [13106.m02620](http://rice.plantbiology.msu.edu/cgi-bin/sequence_display.cgi?orf=13106.m02620) | 13106.t02247 | n/a | 741 | 247 | 26525.3 | 6.26 | 61-221 |  |
| *OsGELP84* | [Os06g34070](http://rice.plantbiology.msu.edu/cgi-bin/ORF_infopage.cgi?&orf=LOC_Os06g34070) | [13106.m03520](http://rice.plantbiology.msu.edu/cgi-bin/sequence_display.cgi?orf=13106.m03520) | 13106.t03083 | [AK059898](http://www.ncbi.nlm.nih.gov/entrez/viewer.fcgi?db=nucleotide&val=AK059898) | 1128 | 376 | 40837.5 | 9.01 | 36-361 |  |
| *OsGELP85* | [Os06g34120](http://rice.plantbiology.msu.edu/cgi-bin/ORF_infopage.cgi?&orf=LOC_Os06g34120) | [13106.m03525](http://rice.plantbiology.msu.edu/cgi-bin/sequence_display.cgi?orf=13106.m03525) | 13106.t03088 | [AK067909](http://www.ncbi.nlm.nih.gov/entrez/viewer.fcgi?db=nucleotide&val=AK067909) | 1188 | 396 | 42825.8 | 8.94 | 8-383 |  |
| *OsGELP86* | [Os06g36520](http://rice.plantbiology.msu.edu/cgi-bin/ORF_infopage.cgi?&orf=LOC_Os06g36520) | [13106.m03778](http://rice.plantbiology.msu.edu/cgi-bin/sequence_display.cgi?orf=13106.m03778) | 13106.t03327 | [AK063332](http://www.ncbi.nlm.nih.gov/entrez/viewer.fcgi?db=nucleotide&val=AK063332) | 1224 | 408 | 43460.9 | 9.95 | 53-387 |  |
| *OsGELP87* | [Os06g43044](http://rice.plantbiology.msu.edu/cgi-bin/ORF_infopage.cgi?&orf=LOC_Os06g43044) | [13106.m04505](http://rice.plantbiology.msu.edu/cgi-bin/sequence_display.cgi?orf=13106.m04505) | 13106.t03974 | [AK058562](http://www.ncbi.nlm.nih.gov/entrez/viewer.fcgi?db=nucleotide&val=AK058562) | 1149 | 383 | 41050.1 | 7.91 | 62-372 |  |
| *OsGELP88* | [Os06g47910](http://rice.plantbiology.msu.edu/cgi-bin/ORF_infopage.cgi?&orf=LOC_Os06g47910) | [13106.m05092](http://rice.plantbiology.msu.edu/cgi-bin/sequence_display.cgi?orf=13106.m05092) | 13106.t04458 | [AK106962](http://www.ncbi.nlm.nih.gov/entrez/viewer.fcgi?db=nucleotide&val=AK106962) | 1140 | 380 | 41071.5 | 5.41 | 38-362 |  |
| *OsGELP89* | [Os06g50940](http://rice.plantbiology.msu.edu/cgi-bin/ORF_infopage.cgi?&orf=LOC_Os06g50940) | [13106.m05484](http://rice.plantbiology.msu.edu/cgi-bin/sequence_display.cgi?orf=13106.m05484) | 13106.t04762 | [AK110292](http://www.ncbi.nlm.nih.gov/entrez/viewer.fcgi?db=nucleotide&val=AK110292) | 1146 | 382 | 39639 | 9.57 | 43-360 |  |
| *OsGELP90* | [Os06g50950](http://rice.plantbiology.msu.edu/cgi-bin/ORF_infopage.cgi?&orf=LOC_Os06g50950) | [13106.m05485](http://rice.plantbiology.msu.edu/cgi-bin/sequence_display.cgi?orf=13106.m05485) | [13106.t04763](http://www.tigr.org/tigr-scripts/euk_manatee/shared/seq_display.cgi?db=osa1r5&orf=12006.m09619) | [AK065261](http://www.ncbi.nlm.nih.gov/entrez/viewer.fcgi?db=nucleotide&val=AK065261) | 1167 | 389 | 40871.8 | 8.99 | 44-367 |  |
| *OsGELP91* | [Os07g39740](http://rice.plantbiology.msu.edu/cgi-bin/ORF_infopage.cgi?&orf=LOC_Os07g39740) | [13107.m04121](http://rice.plantbiology.msu.edu/cgi-bin/sequence_display.cgi?orf=13107.m04121) | 13107.t03624 | [AK071404](http://www.ncbi.nlm.nih.gov/entrez/viewer.fcgi?db=nucleotide&val=AK071404) | 1221 | 406 | 43767.5 | 7.90 | 50-382 |  |
| *OsGELP92a* | [Os07g39750.1](http://rice.plantbiology.msu.edu/cgi-bin/ORF_infopage.cgi?&orf=LOC_Os07g39750) | [13107.m04124](http://rice.plantbiology.msu.edu/cgi-bin/sequence_display.cgi?orf=13107.m04124) | 3107.t03625 | [AK073754](http://www.ncbi.nlm.nih.gov/entrez/viewer.fcgi?db=nucleotide&val=AK073754) | 1176 | 392 | 42490.1 | 7.33 | 37-369 |  |
| *OsGELP92b* | [Os07g39750.2](http://rice.plantbiology.msu.edu/cgi-bin/ORF_infopage.cgi?orf=13107.m04122) | [13107.m04122](http://rice.plantbiology.msu.edu/cgi-bin/sequence_display.cgi?orf=13107.m04122) | 13107.t03625 | n/a | 795 | 265 | 29112.12 | 9.26 | 37-207 |  |
| *OsGELP92c* | [Os07g39750.3](http://rice.plantbiology.msu.edu/cgi-bin/ORF_infopage.cgi?orf=13107.m04123) | [13107.m04123](http://rice.plantbiology.msu.edu/cgi-bin/sequence_display.cgi?orf=13107.m04123) | 13107.t03625 | n/a | 516 | 172 | 18120.4 | 9.14 | 37-148 |  |
| *OsGELP93* | [Os07g44780](http://rice.plantbiology.msu.edu/cgi-bin/ORF_infopage.cgi?&orf=LOC_Os07g44780) | [13107.m04714](http://rice.plantbiology.msu.edu/cgi-bin/sequence_display.cgi?orf=13107.m04714) | 13107.t04106 | [AK058442](http://www.ncbi.nlm.nih.gov/entrez/viewer.fcgi?db=nucleotide&val=AK058442) | 1176 | 392 | 42013.0 | 8.46 | 49-374 |  |
| *OsGELP94a* | [Os07g47210](http://rice.plantbiology.msu.edu/cgi-bin/ORF_infopage.cgi?&orf=LOC_Os07g47210).1 | [13107.m05016](http://rice.plantbiology.msu.edu/cgi-bin/sequence_display.cgi?orf=13107.m05016) | 13107.t04351 | n/a | 1155 | 385 | 41187.1 | 5.80 | 47-365 |  |
| *OsGELP94b* | [Os07g47210.2](http://rice.plantbiology.msu.edu/cgi-bin/ORF_infopage.cgi?orf=13107.m05017) | [13107.m05017](http://rice.plantbiology.msu.edu/cgi-bin/sequence_display.cgi?orf=13107.m05017) | 13107.t04351 | [AK061122](http://www.ncbi.nlm.nih.gov/entrez/viewer.fcgi?db=nucleotide&val=AK061122) | 678 | 226 | 23683.1 | 4.92 | 47-216 |  |
| *OsGELP95* | [Os08g02094](http://rice.plantbiology.msu.edu/cgi-bin/ORF_infopage.cgi?&orf=LOC_Os08g02094) | [13108.m00143](http://rice.plantbiology.msu.edu/cgi-bin/sequence_display.cgi?orf=13108.m00143) | 13108.t00110 | [AK105876](http://www.ncbi.nlm.nih.gov/entrez/viewer.fcgi?db=nucleotide&val=AK105876) | 1146 | 382 | 40006.7 | 5.77 | 51-362 |  |
| *OsGELP96a* | [Os08g45150](http://rice.plantbiology.msu.edu/cgi-bin/ORF_infopage.cgi?&orf=LOC_Os08g45150).1 | [13108.m04922](http://rice.plantbiology.msu.edu/cgi-bin/sequence_display.cgi?orf=13108.m04922) | 13108.t04247 | [AK106778](http://www.ncbi.nlm.nih.gov/entrez/viewer.fcgi?db=nucleotide&val=AK106778) | 1164 | 388 | 41812.5 | 6.98 | 46-359 |  |
| *OsGELP96b* | [Os08g45150](http://rice.plantbiology.msu.edu/cgi-bin/ORF_infopage.cgi?orf=13108.m09402).2 | [13108.m09402](http://rice.plantbiology.msu.edu/cgi-bin/sequence_display.cgi?orf=13108.m09402) | 13108.t04247 | n/a | 987 | 328 | 35325.2 | 12.59 | 46-359 |  |
| *OsGELP97a* | [Os09g04624.1](http://rice.plantbiology.msu.edu/cgi-bin/ORF_infopage.cgi?&orf=LOC_Os09g04624) | [13109.m00394](http://rice.plantbiology.msu.edu/cgi-bin/sequence_display.cgi?orf=13109.m00394) | 13109.t00361 | [AK061026](http://www.ncbi.nlm.nih.gov/entrez/viewer.fcgi?db=nucleotide&val=AK061026) | 1056 | 352 | 37690.4 | 4.37 | 30-342 |  |
| *OsGELP97b* | [Os09g04624.2](http://rice.plantbiology.msu.edu/cgi-bin/ORF_infopage.cgi?orf=13109.m00395) | [13109.m00395](http://rice.plantbiology.msu.edu/cgi-bin/sequence_display.cgi?orf=13109.m00395) | [13109.t00361](http://www.tigr.org/tigr-scripts/euk_manatee/shared/seq_display.cgi?db=osa1r5&orf=12009.m22215) | [AK060124](http://www.ncbi.nlm.nih.gov/entrez/viewer.fcgi?db=nucleotide&val=AK060124) | 867 | 289 | 30756.5 | 4.32 | 30-288 |  |
| *OsGELP98a* | [Os09g04710.1](http://rice.plantbiology.msu.edu/cgi-bin/ORF_infopage.cgi?orf=13109.m00404) | [13109.m00404](http://rice.plantbiology.msu.edu/cgi-bin/sequence_display.cgi?orf=13109.m00404) | 13109.t00369 | [AK059766](http://www.ncbi.nlm.nih.gov/entrez/viewer.fcgi?db=nucleotide&val=AK059766) | 1059 | 353 | 38245.3 | 4.59 | 31-343 |  |
| *OsGELP98b* | [Os09g04710.2](http://rice.plantbiology.msu.edu/cgi-bin/ORF_infopage.cgi?orf=13109.m00405) | [13109.m00405](http://rice.plantbiology.msu.edu/cgi-bin/sequence_display.cgi?orf=13109.m00405) | 13109.t00369 | [AK059484](http://www.ncbi.nlm.nih.gov/entrez/viewer.fcgi?db=nucleotide&val=AK059484) | 1035 | 345 | 37417.4 | 4.64 | 31-335 |  |
| *OsGELP99* | [Os09g07290](http://rice.plantbiology.msu.edu/cgi-bin/ORF_infopage.cgi?&orf=LOC_Os09g07290) | [13109.m00573](http://rice.plantbiology.msu.edu/cgi-bin/sequence_display.cgi?orf=13109.m00573) | 13109.t00525 | [AK107173](http://www.ncbi.nlm.nih.gov/entrez/viewer.fcgi?db=nucleotide&val=AK107173) | 1071 | 357 | 38214.6 | 7.59 | 35-347 |  |
| *OsGELP100a* | [Os09g36880.1](http://rice.plantbiology.msu.edu/cgi-bin/ORF_infopage.cgi?&orf=LOC_Os09g36880) | [13109.m03643](http://rice.plantbiology.msu.edu/cgi-bin/sequence_display.cgi?orf=13109.m03643) | 13109.t03172 | [AK112006](http://www.ncbi.nlm.nih.gov/entrez/viewer.fcgi?db=nucleotide&val=AK112006) | 1101 | 367 | 39471.8 | 4.82 | 41-357 |  |
| *OsGELP100b* | [Os09g36880.2](http://rice.plantbiology.msu.edu/cgi-bin/ORF_infopage.cgi?orf=13109.m03644) | [13109.m03644](http://rice.plantbiology.msu.edu/cgi-bin/sequence_display.cgi?orf=13109.m03644) | 13109.t03172 | [AK111927](http://www.ncbi.nlm.nih.gov/entrez/viewer.fcgi?db=nucleotide&val=AK111927) | 975 | 325 | 34490.1 | 4.85 | 41-306 |  |
| *OsGELP101* | [Os09g39430](http://rice.plantbiology.msu.edu/cgi-bin/ORF_infopage.cgi?&orf=LOC_Os09g39430) | [13109.m03952](http://rice.plantbiology.msu.edu/cgi-bin/sequence_display.cgi?orf=13109.m03952) | 13109.t03427 | n/a | 1149 | 383 | 41322.4 | 6.37 | 74-372 |  |
| *OsGELP102* | [Os10g05088](http://rice.plantbiology.msu.edu/cgi-bin/ORF_infopage.cgi?&orf=LOC_Os10g05088) | [13110.m00425](http://rice.plantbiology.msu.edu/cgi-bin/sequence_display.cgi?orf=13110.m00425) | 13110.t00381 | [AK100609](http://www.ncbi.nlm.nih.gov/entrez/viewer.fcgi?db=nucleotide&val=AK100609) | 1050 | 350 | 37473.5 | 8.82 | 40-341 |  |
| *OsGELP103* | [Os10g25340](http://rice.plantbiology.msu.edu/cgi-bin/ORF_infopage.cgi?&orf=LOC_Os10g25340) | [13110.m02137](http://rice.plantbiology.msu.edu/cgi-bin/sequence_display.cgi?orf=13110.m02137) | 13110.t01949 | n/a | 1230 | 410 | 43259.5 | 8.47 | 1.47-373 |  |
| *OsGELP104* | [Os10g25380](http://rice.plantbiology.msu.edu/cgi-bin/ORF_infopage.cgi?&orf=LOC_Os10g25380) | [13110.m02140](http://rice.plantbiology.msu.edu/cgi-bin/sequence_display.cgi?orf=13110.m02140) | 13110.t01952 | n/a | 1161 | 387 | 40213.5 | 8.13 | 52-382 |  |
| *OsGELP105* | [Os10g25400](http://rice.plantbiology.msu.edu/cgi-bin/ORF_infopage.cgi?&orf=LOC_Os10g25400) | [13110.m02141](http://rice.plantbiology.msu.edu/cgi-bin/sequence_display.cgi?orf=13110.m02141) | 13110.t01953 | [AK108245](http://www.ncbi.nlm.nih.gov/entrez/viewer.fcgi?db=nucleotide&val=AK108245) | 1206 | 402 | 40772 | 6.50 | 34-369 |  |
| *OsGELP106* | [Os10g25420](http://rice.plantbiology.msu.edu/cgi-bin/ORF_infopage.cgi?&orf=LOC_Os10g25420) | [13110.m02143](http://rice.plantbiology.msu.edu/cgi-bin/sequence_display.cgi?orf=13110.m02143) | 13110.t01955 | [AK110763](http://www.ncbi.nlm.nih.gov/entrez/viewer.fcgi?db=nucleotide&val=AK110763) | 1017 | 339 | 36177.3 | 9.20 | 185-338 |  |
| *OsGELP107* | [Os10g30290](http://rice.plantbiology.msu.edu/cgi-bin/ORF_infopage.cgi?&orf=LOC_Os10g30290) | [13110.m02604](http://rice.plantbiology.msu.edu/cgi-bin/sequence_display.cgi?orf=13110.m02604) | 13110.t02362 | [AK065583](http://www.ncbi.nlm.nih.gov/entrez/viewer.fcgi?db=nucleotide&val=AK065583) | 1077 | 359 | 39079.6 | 6.72 | 39-352 |  |
| *OsGELP108* | [Os10g32580](http://rice.plantbiology.msu.edu/cgi-bin/ORF_infopage.cgi?&orf=LOC_Os10g32580) | [13110.m02846](http://rice.plantbiology.msu.edu/cgi-bin/sequence_display.cgi?orf=13110.m02846) | [13110.t02558](http://www.tigr.org/tigr-scripts/euk_manatee/shared/seq_display.cgi?db=osa1r5&orf=12010.m06095) | [AK108201](http://www.ncbi.nlm.nih.gov/entrez/viewer.fcgi?db=nucleotide&val=AK108201) | 1173 | 391 | 42066.1 | 9.48 | 29-342 |  |
| *sGELP109* | [Os10g33690](http://rice.plantbiology.msu.edu/cgi-bin/ORF_infopage.cgi?&orf=LOC_Os10g33690) | [13110.m02972](http://rice.plantbiology.msu.edu/cgi-bin/sequence_display.cgi?orf=13110.m02972) | 13110.t02662 | [AK070445](http://www.ncbi.nlm.nih.gov/entrez/viewer.fcgi?db=nucleotide&val=AK070445) | 813 | 271 | 29769.9 | 6.78 | 37-236 |  |
| *OsGELP110* | [Os11g03520](http://rice.plantbiology.msu.edu/cgi-bin/ORF_infopage.cgi?&orf=LOC_Os11g03520) | [13111.m00292](http://rice.plantbiology.msu.edu/cgi-bin/sequence_display.cgi?orf=13111.m00292) | 13111.t00245 | [AK107889](http://www.ncbi.nlm.nih.gov/nuccore/32993098?report=genbank) | 1089 | 363 | 38244.2 | 7.68 | 182-330 |  |
| *OsGELP111* | [Os11g31940](http://rice.plantbiology.msu.edu/cgi-bin/ORF_infopage.cgi?&orf=LOC_Os11g31940) | [13111.m03104](http://rice.plantbiology.msu.edu/cgi-bin/sequence_display.cgi?orf=13111.m03104) | 13111.t02786 | [AK108542](http://www.ncbi.nlm.nih.gov/entrez/viewer.fcgi?db=nucleotide&val=AK108542) | 1122 | 374 | 39948.6 | 4.97 | 31-355 |  |
| *OsGELP112* | [Os11g48070](http://rice.plantbiology.msu.edu/cgi-bin/ORF_infopage.cgi?&orf=LOC_Os11g48070) | [13111.m04805](http://rice.plantbiology.msu.edu/cgi-bin/sequence_display.cgi?orf=13111.m04805) | 13111.t04295 | [AK067429](http://www.ncbi.nlm.nih.gov/entrez/viewer.fcgi?db=nucleotide&val=AK067429) | 783 | 261 | 29115.1 | 5.16 | 13-207 | *WDL1* |
| *OsGELP113* | [Os12g17570](http://rice.plantbiology.msu.edu/cgi-bin/ORF_infopage.cgi?&orf=LOC_Os12g17570) | [13112.m01861](http://rice.plantbiology.msu.edu/cgi-bin/sequence_display.cgi?orf=13112.m01861) | 13112.t01609 | [AK063071](http://www.ncbi.nlm.nih.gov/entrez/viewer.fcgi?db=nucleotide&val=AK063071) | 846 | 281 | 43882.2 | 7.40 | 29-342 |  |
| *OsGELP114a* | [Os12g37910](http://rice.plantbiology.msu.edu/cgi-bin/ORF_infopage.cgi?orf=13112.m03904).1 | [13112.m03904](http://rice.plantbiology.msu.edu/cgi-bin/sequence_display.cgi?orf=13112.m03904) | 13112.t03486 | n/a | 1176 | 391 | 41665.4 | 9.49 | 46-250 |  |
| *OsGELP114b* | [Os12g37910.2](http://rice.plantbiology.msu.edu/cgi-bin/ORF_infopage.cgi?orf=13112.m03903) | [13112.m03903](http://rice.plantbiology.msu.edu/cgi-bin/sequence_display.cgi?orf=13112.m03903) | 13112.t03486 | [AK060201](http://www.ncbi.nlm.nih.gov/nuccore/32970219?report=genbank) | 1209 | 402 | 42416.8 | 6.50 | 46-365 |  |
