# Supplementary material for: Multifunctionality and diversity of GDSL esterase/lipase gene family in rice (Oryza sativa L. japonica) genome: new insights from bioinformatics analysis
Source: BMC Genomics. 2012 Jul 15;13:309. doi: 10.1186/1471-2164-13-309 (PMC3412167; doi:10.1186/1471-2164-13-309)
Supplement: Additional file 2 — Expression evidence for the OsGELP rice genes. The OsGELP gene names, locus ID, MPSS signature sequences, FL-cDNA number, total quantity of mapped ESTs, and the presence of microarray data from Genevestigator for each of 153 transcripts (including alternative spliced models) of the 114 OsGELP genes are given. [file 1471-2164-13-309-S2.doc]

**Additional file 2.** Expression evidence for the *OsGELP* rice genes.

| **Gene Name** | **MSU Osa1 Release 6.1 Locus ID** | **Full length cDNA** | **Total number of mapped ESTs** |
| --- | --- | --- | --- |
| *OsGELP1* | LOC_Os01g11570.1 | [AK061229](http://www.ncbi.nlm.nih.gov/entrez/viewer.fcgi?db=nucleotide&val=AK061229) | 29 |
| *OsGELP2a* | LOC_Os01g11620.1 | n/a | 82 |
| *OsGELP2b* | LOC_Os01g11620.2 | [AK103614](http://www.ncbi.nlm.nih.gov/entrez/viewer.fcgi?db=nucleotide&val=AK103614) | 80 |
| *OsGELP3a* | LOC_Os01g11650.1 | [AK065001](http://www.ncbi.nlm.nih.gov/entrez/viewer.fcgi?db=nucleotide&val=AK065001) | 101 |
| *OsGELP3b* | LOC_Os01g11650.2 | [AK069801](http://www.ncbi.nlm.nih.gov/entrez/viewer.fcgi?db=nucleotide&val=AK069801) | 31 |
| *OsGELP4* | LOC_Os01g11660.1 | [AK100754](http://www.ncbi.nlm.nih.gov/entrez/viewer.fcgi?db=nucleotide&val=AK100754) | 3 |
| *OsGELP5a* | LOC_Os01g11700.1 | n/a | 5 |
| *OsGELP5b* | LOC_Os01g11700.2 | [AK059318](http://www.ncbi.nlm.nih.gov/entrez/viewer.fcgi?db=nucleotide&val=AK059318) [AK102384](http://www.ncbi.nlm.nih.gov/entrez/viewer.fcgi?db=nucleotide&val=AK102384) | 7 |
| *OsGELP6* | LOC_Os01g11710.1 | [AK059088](http://www.ncbi.nlm.nih.gov/entrez/viewer.fcgi?db=nucleotide&val=AK059088) [AK061118](http://www.ncbi.nlm.nih.gov/entrez/viewer.fcgi?db=nucleotide&val=AK061118) [AK119598](http://www.ncbi.nlm.nih.gov/entrez/viewer.fcgi?db=nucleotide&val=AK119598) [AK119642](http://www.ncbi.nlm.nih.gov/entrez/viewer.fcgi?db=nucleotide&val=AK119642) | 142 |
| *OsGELP7* | LOC_Os01g11730.1 | [AK058429](http://www.ncbi.nlm.nih.gov/entrez/viewer.fcgi?db=nucleotide&val=AK058429)  [AK104443](http://www.ncbi.nlm.nih.gov/entrez/viewer.fcgi?db=nucleotide&val=AK104443) | 23 |
| *OsGELP8* | LOC_Os01g11740.1 | [AK107557](http://www.ncbi.nlm.nih.gov/entrez/viewer.fcgi?db=nucleotide&val=AK107557) | 2 |
| *OsGELP9* | LOC_Os01g11750.1 | n/a | 0 |
| *OsGELP10* | LOC_Os01g11760.1 | n/a | 4 |
| *OsGELP11* | LOC_Os01g11790.1 | n/a | 1 |
| *OsGELP12a* | LOC_Os01g12320.1 | [AK067352](http://www.ncbi.nlm.nih.gov/entrez/viewer.fcgi?db=nucleotide&val=AK067352) | 29 |
| *OsGELP12b* | LOC_Os01g12320.2 | n/a | 28 |
| *OsGELP12c* | LOC_Os01g12320.3 | n/a | 26 |
| *OsGELP13* | LOC_Os01g12381.1 | n/a | 0 |
| *OsGELP14* | LOC_Os01g22640.1 | [AK120056](http://www.ncbi.nlm.nih.gov/entrez/viewer.fcgi?db=nucleotide&val=AK120056) | 18 |
| *OsGELP15a* | LOC_Os01g22660.1 | n/a | 24 |
| *OsGELP15b* | LOC_Os01g22660.2 | [AK100480](http://www.ncbi.nlm.nih.gov/entrez/viewer.fcgi?db=nucleotide&val=AK100480) | 23 |
| *OsGELP15c* | LOC_Os01g22660.3 | n/a | 5 |
| *OsGELP16a* | LOC_Os01g22780.1 | [AK100992](http://www.ncbi.nlm.nih.gov/entrez/viewer.fcgi?db=nucleotide&val=AK100992) | 4 |
| *OsGELP16b* | LOC_Os01g22780.2 | n/a | 5 |
| *OsGELP17* | LOC_Os01g42730.1 | n/a | 36 |
| *OsGELP18a* | LOC_Os01g46080.1 | [AK105142](http://www.ncbi.nlm.nih.gov/entrez/viewer.fcgi?db=nucleotide&val=AK105142) | 25 |
| *OsGELP18b* | LOC_Os01g46080.2 | n/a | 3 |
| *OsGELP19* | LOC_Os01g46090.1 | n/a | 0 |
| *OsGELP20a* | LOC_Os01g46120.1 | n/a | 0 |
| *OsGELP20b* | LOC_Os01g46120.2 | n/a | 8 |
| *OsGELP20c* | LOC_Os01g46120.3 | n/a | 9 |
| *OsGELP21a* | LOC_Os01g46169.1 | [AK064049](http://www.ncbi.nlm.nih.gov/entrez/viewer.fcgi?db=nucleotide&val=AK064049) | 23 |
| *OsGELP21b* | LOC_Os01g46169.2 | [AK102591](http://www.ncbi.nlm.nih.gov/entrez/viewer.fcgi?db=nucleotide&val=AK102591) | 19 |
| *OsGELP21c* | LOC_Os01g46169.3 | [AK062412](http://www.ncbi.nlm.nih.gov/entrez/viewer.fcgi?db=nucleotide&val=AK062412) | 12 |
| *OsGELP22* | LOC_Os01g46210.1 | n/a | 0 |
| *OsGELP23* | LOC_Os01g46220.1 | n/a | 2 |
| *OsGELP24* | LOC_Os01g52770.1 | [AK104682](http://www.ncbi.nlm.nih.gov/entrez/viewer.fcgi?db=nucleotide&val=AK104682) | 36 |
| *OsGELP25* | LOC_Os01g54470.1 | n/a | 10 |
| *OsGELP26* | LOC_Os01g61200.1 | n/a | 9 |
| *OsGELP27* | LOC_Os01g61570.1 | [AK064727](http://www.ncbi.nlm.nih.gov/entrez/viewer.fcgi?db=nucleotide&val=AK064727) | 13 |
| *OsGELP28* | LOC_Os01g72850.1 | n/a | 0 |
| *OsGELP29* | LOC_Os02g01140.1 | [AK067036](http://www.ncbi.nlm.nih.gov/entrez/viewer.fcgi?db=nucleotide&val=AK067036) | 11 |
| *OsGELP30* | LOC_Os02g01980.1 | [AK106946](http://www.ncbi.nlm.nih.gov/entrez/viewer.fcgi?db=nucleotide&val=AK106946) | 86 |
| *OsGELP31* | LOC_Os02g09610.1 | [AK108567](http://www.ncbi.nlm.nih.gov/entrez/viewer.fcgi?db=nucleotide&val=AK108567) | 2 |
| *OsGELP32* | LOC_Os02g09620.1 | [AK060691](http://www.ncbi.nlm.nih.gov/entrez/viewer.fcgi?db=nucleotide&val=AK060691) | 3 |
| *OsGELP33a* | LOC_Os02g15230.1 | n/a | 90 |
| *OsGELP33b* | LOC_Os02g15230.2 | [AK061300](http://www.ncbi.nlm.nih.gov/entrez/viewer.fcgi?db=nucleotide&val=AK061300) [AK070671](http://www.ncbi.nlm.nih.gov/entrez/viewer.fcgi?db=nucleotide&val=AK070671) | 59 |
| *OsGELP33c* | LOC_Os02g15230.3 | [AK061300](http://www.ncbi.nlm.nih.gov/entrez/viewer.fcgi?db=nucleotide&val=AK061300) | 70 |
| *OsGELP34* | LOC_Os02g18870.1 | n/a | 1 |
| *OsGELP35* | LOC_Os02g18954.1 | [AK101883](http://www.ncbi.nlm.nih.gov/entrez/viewer.fcgi?db=nucleotide&val=AK101883) | 2 |
| *OsGELP36* | LOC_Os02g18990.1 | n/a | 0 |
| *OsGELP37* | LOC_Os02g19040.1 | [AK106688](http://www.ncbi.nlm.nih.gov/entrez/viewer.fcgi?db=nucleotide&val=AK106688) | 4 |
| *OsGELP38* | LOC_Os02g39170.1 | n/a | 0 |
| *OsGELP39* | LOC_Os02g39590.1 | n/a | 0 |
| *OsGELP40* | LOC_Os02g40440.1 | [AK108108](http://www.ncbi.nlm.nih.gov/entrez/viewer.fcgi?db=nucleotide&val=AK108108) | 10 |
| *OsGELP41* | LOC_Os02g44850.1 | n/a | 0 |
| *OsGELP42* | LOC_Os02g44860.1 | [AK059531](http://www.ncbi.nlm.nih.gov/entrez/viewer.fcgi?db=nucleotide&val=AK059531) [AK103585](http://www.ncbi.nlm.nih.gov/entrez/viewer.fcgi?db=nucleotide&val=AK103585) | 35 |
| *OsGELP43* | LOC_Os02g50000.1 | n/a | 6 |
| *OsGELP44a* | LOC_Os02g50690.1 | [AK070261](http://www.ncbi.nlm.nih.gov/entrez/viewer.fcgi?db=nucleotide&val=AK070261) [AK099325](http://www.ncbi.nlm.nih.gov/entrez/viewer.fcgi?db=nucleotide&val=AK099325) | 52 |
| *OsGELP44b* | LOC_Os02g50690.2 | [AK111360](http://www.ncbi.nlm.nih.gov/entrez/viewer.fcgi?db=nucleotide&val=AK111360) | 0 |
| *OsGELP45* | LOC_Os02g57110.1 | [AK060243](http://www.ncbi.nlm.nih.gov/entrez/viewer.fcgi?db=nucleotide&val=AK060243) [AK099251](http://www.ncbi.nlm.nih.gov/entrez/viewer.fcgi?db=nucleotide&val=AK099251) [AK106084](http://www.ncbi.nlm.nih.gov/entrez/viewer.fcgi?db=nucleotide&val=AK106084) | 59 |
| *OsGELP46a* | LOC_Os03g19670.1 | [AK060625](http://www.ncbi.nlm.nih.gov/entrez/viewer.fcgi?db=nucleotide&val=AK060625) | 15 |
| *OsGELP46b* | LOC_Os03g19670.2 | n/a | 3 |
| *OsGELP47* | LOC_Os03g25000.1 | n/a | 0 |
| *OsGELP48* | LOC_[Os03g25010](http://rice.plantbiology.msu.edu/cgi-bin/ORF_infopage.cgi?&orf=LOC_Os03g25010).1 | n/a | 0 |
| *OsGELP49* | LOC_Os03g25030.1 | [AK063672](http://www.ncbi.nlm.nih.gov/entrez/viewer.fcgi?db=nucleotide&val=AK063672) | 25 |
| *OsGELP50a* | LOC_Os03g25040.1 | n/a | 9 |
| *OsGELP50b* | LOC_Os03g25040.2 | [AK069388](http://www.ncbi.nlm.nih.gov/entrez/viewer.fcgi?db=nucleotide&val=AK069388) | 13 |
| *OsGELP51* | LOC_Os03g38390.1 | [AK070980](http://www.ncbi.nlm.nih.gov/entrez/viewer.fcgi?db=nucleotide&val=AK070980) | 12 |
| *OsGELP52* | LOC_Os03g38470.1 | n/a | 1 |
| *OsGELP53* | LOC_Os03g47940.1 | [AK099266](http://www.ncbi.nlm.nih.gov/entrez/viewer.fcgi?db=nucleotide&val=AK099266) | 170 |
| *OsGELP54* | LOC_Os03g62740.1 | n/a | 0 |
| *OsGELP55a* | LOC_Os03g64170.1 | [AK060992](http://www.ncbi.nlm.nih.gov/entrez/viewer.fcgi?db=nucleotide&val=AK060992) [AK120038](http://www.ncbi.nlm.nih.gov/entrez/viewer.fcgi?db=nucleotide&val=AK120038) | 35 |
| *OsGELP55b* | LOC_Os03g64170.2 | [AK120038](http://www.ncbi.nlm.nih.gov/entrez/viewer.fcgi?db=nucleotide&val=AK120038) | 30 |
| *OsGELP56* | LOC_Os04g42860.1 | [AK121383](http://www.ncbi.nlm.nih.gov/entrez/viewer.fcgi?db=nucleotide&val=AK121383) | 22 |
| *OsGELP57* | LOC_Os04g47390.1 | n/a | 0 |
| *OsGELP58a* | LOC_Os04g48800.1 | n/a | 38 |
| *OsGELP58b* | LOC_Os04g48800.2 | [AK120597](http://www.ncbi.nlm.nih.gov/entrez/viewer.fcgi?db=nucleotide&val=AK120597) | 41 |
| *OsGELP58c* | LOC_Os04g48800.3 | n/a | 46 |
| *OsGELP58d* | LOC_Os04g48800.4 | n/a | 24 |
| *OsGELP59* | LOC_Os04g55660.1 | [AK072573](http://www.ncbi.nlm.nih.gov/entrez/viewer.fcgi?db=nucleotide&val=AK072573) [AK109786](http://www.ncbi.nlm.nih.gov/entrez/viewer.fcgi?db=nucleotide&val=AK109786) | 2 |
| *OsGELP60* | LOC_Os05g04240.1 | n/a | 0 |
| *OsGELP61* | LOC_Os05g06710.1 | [AK067837](http://www.ncbi.nlm.nih.gov/entrez/viewer.fcgi?db=nucleotide&val=AK067837) | 26 |
| *OsGELP62a* | LOC_Os05g06720.1 | n/a | 27 |
| *OsGELP62b* | LOC_Os05g06720.2 | n/a | 25 |
| *OsGELP62c* | LOC_Os05g06720.3 | [AK070904](http://www.ncbi.nlm.nih.gov/entrez/viewer.fcgi?db=nucleotide&val=AK070904) | 22 |
| *OsGELP63* | LOC_Os05g11910.1 | [AK059511](http://www.ncbi.nlm.nih.gov/entrez/viewer.fcgi?db=nucleotide&val=AK059511) [AK100289](http://www.ncbi.nlm.nih.gov/entrez/viewer.fcgi?db=nucleotide&val=AK100289) [AY580163](http://www.ncbi.nlm.nih.gov/entrez/viewer.fcgi?db=nucleotide&val=AY580163) | 118 |
| *OsGELP64a* | LOC_Os05g11950.1 | [AK122049](http://www.ncbi.nlm.nih.gov/entrez/viewer.fcgi?db=nucleotide&val=AK122049) | 39 |
| *OsGELP64b* | LOC_Os05g11950.2 | n/a | 1 |
| *OsGELP65* | LOC_Os05g11970.1 | n/a | 2 |
| *OsGELP66* | LOC_Os05g33270.1 | n/a | 23 |
| *OsGELP67a* | LOC_Os05g34700.1 | [AK100958](http://www.ncbi.nlm.nih.gov/entrez/viewer.fcgi?db=nucleotide&val=AK100958) | 58 |
| *OsGELP67b* | LOC_Os05g34700.2 | n/a | 32 |
| *OsGELP68* | LOC_Os05g39220.1 | [AK064681](http://www.ncbi.nlm.nih.gov/entrez/viewer.fcgi?db=nucleotide&val=AK064681) | 1 |
| *OsGELP69* | LOC_Os05g43090.1 | n/a | 0 |
| *OsGELP70* | LOC_Os05g43100.1 | n/a | 0 |
| *OsGELP71* | LOC_Os05g43110.1 | n/a | 0 |
| *OsGELP72* | LOC_Os05g43120.1 | n/a | 2 |
| *OsGELP73* | LOC_Os05g44200.1 | [AK061147](http://www.ncbi.nlm.nih.gov/entrez/viewer.fcgi?db=nucleotide&val=AK061147) | 74 |
| *OsGELP74* | LOC_Os06g05550.1 | [AK059204](http://www.ncbi.nlm.nih.gov/entrez/viewer.fcgi?db=nucleotide&val=AK059204) [AK060175](http://www.ncbi.nlm.nih.gov/entrez/viewer.fcgi?db=nucleotide&val=AK060175) [AK073593](http://www.ncbi.nlm.nih.gov/entrez/viewer.fcgi?db=nucleotide&val=AK073593) [AK109420](http://www.ncbi.nlm.nih.gov/entrez/viewer.fcgi?db=nucleotide&val=AK109420) | 86 |
| *OsGELP75a* | LOC_Os06g05630.1 | n/a | 54 |
| *OsGELP75b* | LOC_Os06g05630.2 | n/a | 34 |
| *OsGELP75c* | LOC_Os06g05630.3 | [AK067418](http://www.ncbi.nlm.nih.gov/entrez/viewer.fcgi?db=nucleotide&val=AK067418) [AK099064](http://www.ncbi.nlm.nih.gov/entrez/viewer.fcgi?db=nucleotide&val=AK099064) | 45 |
| *OsGELP76* | LOC_Os06g06230.1 | n/a | 0 |
| *OsGELP77* | LOC_Os06g06250.1 | [AK061497](http://www.ncbi.nlm.nih.gov/entrez/viewer.fcgi?db=nucleotide&val=AK061497) [AK104394](http://www.ncbi.nlm.nih.gov/entrez/viewer.fcgi?db=nucleotide&val=AK104394) [AK104883](http://www.ncbi.nlm.nih.gov/entrez/viewer.fcgi?db=nucleotide&val=AK104883) [AK121780](http://www.ncbi.nlm.nih.gov/entrez/viewer.fcgi?db=nucleotide&val=AK121780) | 155 |
| *OsGELP78* | LOC_Os06g06260.1 | [AK107226](http://www.ncbi.nlm.nih.gov/entrez/viewer.fcgi?db=nucleotide&val=AK107226) | 43 |
| *OsGELP79* | LOC_Os06g06290.1 | [AK066113](http://www.ncbi.nlm.nih.gov/entrez/viewer.fcgi?db=nucleotide&val=AK066113) | 246 |
| *OsGELP80* | LOC_Os06g06520.1 | [AK073443](http://www.ncbi.nlm.nih.gov/entrez/viewer.fcgi?db=nucleotide&val=AK073443) | 29 |
| *OsGELP81* | LOC_Os06g12410.1 | n/a | 2 |
| *OsGELP82* | LOC_Os06g14630.1 | [AK108686](http://www.ncbi.nlm.nih.gov/entrez/viewer.fcgi?db=nucleotide&val=AK108686) | 1 |
| *OsGELP83a* | LOC_Os06g24404.1 | [AK061188](http://www.ncbi.nlm.nih.gov/entrez/viewer.fcgi?db=nucleotide&val=AK061188) [AK067876](http://www.ncbi.nlm.nih.gov/entrez/viewer.fcgi?db=nucleotide&val=AK067876) [AK099072](http://www.ncbi.nlm.nih.gov/entrez/viewer.fcgi?db=nucleotide&val=AK099072) | 24 |
| *OsGELP83b* | LOC_Os06g24404.2 | n/a | 11 |
| *OsGELP84* | LOC_Os06g34070.1 | [AK059898](http://www.ncbi.nlm.nih.gov/entrez/viewer.fcgi?db=nucleotide&val=AK059898) | 25 |
| *OsGELP85* | LOC_Os06g34120.1 | [AK061422](http://www.ncbi.nlm.nih.gov/entrez/viewer.fcgi?db=nucleotide&val=AK061422) [AK067909](http://www.ncbi.nlm.nih.gov/entrez/viewer.fcgi?db=nucleotide&val=AK067909) | 114 |
| *OsGELP86* | LOC_Os06g36520.1 | [AK063332](http://www.ncbi.nlm.nih.gov/entrez/viewer.fcgi?db=nucleotide&val=AK063332) | 6 |
| *OsGELP87* | LOC_Os06g43044.1 | [AK058562](http://www.ncbi.nlm.nih.gov/entrez/viewer.fcgi?db=nucleotide&val=AK058562) | 21 |
| *OsGELP88* | LOC_Os06g47910.1 | [AK106962](http://www.ncbi.nlm.nih.gov/entrez/viewer.fcgi?db=nucleotide&val=AK106962) | 3 |
| *OsGELP89* | LOC_Os06g50940.1 | [AK110292](http://www.ncbi.nlm.nih.gov/entrez/viewer.fcgi?db=nucleotide&val=AK110292) | 1 |
| *OsGELP90* | LOC_Os06g50950.1 | n/a | 81 |
| *OsGELP91* | LOC_Os07g39740.1 | [AK071404](http://www.ncbi.nlm.nih.gov/entrez/viewer.fcgi?db=nucleotide&val=AK071404) | 11 |
| *OsGELP92a* | LOC_Os07g39750.1 | [AK060131](http://www.ncbi.nlm.nih.gov/entrez/viewer.fcgi?db=nucleotide&val=AK060131) [AK073754](http://www.ncbi.nlm.nih.gov/entrez/viewer.fcgi?db=nucleotide&val=AK073754) [AK099293](http://www.ncbi.nlm.nih.gov/entrez/viewer.fcgi?db=nucleotide&val=AK099293) | 89 |
| *OsGELP92b* | LOC_Os07g39750.2 | n/a | 18 |
| *OsGELP92c* | LOC_Os07g39750.3 | n/a | 17 |
| *OsGELP93* | LOC_Os07g44780.1 | [AK058442](http://www.ncbi.nlm.nih.gov/entrez/viewer.fcgi?db=nucleotide&val=AK058442) | 6 |
| *OsGELP94a* | LOC_Os07g47210.1 | n/a | 7 |
| *OsGELP94b* | LOC_Os07g47210.2 | [AK061122](http://www.ncbi.nlm.nih.gov/entrez/viewer.fcgi?db=nucleotide&val=AK061122) | 2 |
| *OsGELP95* | LOC_Os08g02094.1 | [AK069672](http://www.ncbi.nlm.nih.gov/entrez/viewer.fcgi?db=nucleotide&val=AK069672) [AK105876](http://www.ncbi.nlm.nih.gov/entrez/viewer.fcgi?db=nucleotide&val=AK105876) | 9 |
| *OsGELP96* | LOC_Os08g45150.1 | [AK106778](http://www.ncbi.nlm.nih.gov/entrez/viewer.fcgi?db=nucleotide&val=AK106778) | 63 |
| *OsGELP97a* | LOC_Os09g04624.1 | [AK061026](http://www.ncbi.nlm.nih.gov/entrez/viewer.fcgi?db=nucleotide&val=AK061026) | 12 |
| *OsGELP97b* | LOC_Os09g04624.2 | [AK060124](http://www.ncbi.nlm.nih.gov/entrez/viewer.fcgi?db=nucleotide&val=AK060124) | 11 |
| *OsGELP98a* | LOC_Os09g04710.1 | [AK059766](http://www.ncbi.nlm.nih.gov/entrez/viewer.fcgi?db=nucleotide&val=AK059766) | 40 |
| *OsGELP98b* | LOC_Os09g04710.2 | [AK059484](http://www.ncbi.nlm.nih.gov/entrez/viewer.fcgi?db=nucleotide&val=AK059484) | 38 |
| *OsGELP99* | LOC_Os09g07290.1 | [AK107173](http://www.ncbi.nlm.nih.gov/entrez/viewer.fcgi?db=nucleotide&val=AK107173) | 2 |
| *OsGELP100a* | LOC_Os09g36880.1 | [AK112006](http://www.ncbi.nlm.nih.gov/entrez/viewer.fcgi?db=nucleotide&val=AK112006) | 10 |
| *OsGELP100b* | LOC_Os09g36880.2 | [AK111927](http://www.ncbi.nlm.nih.gov/entrez/viewer.fcgi?db=nucleotide&val=AK111927) | 5 |
| *OsGELP101* | LOC_Os09g39430.1 | n/a | 11 |
| *OsGELP102* | LOC_Os10g05088.1 | [AK100609](http://www.ncbi.nlm.nih.gov/entrez/viewer.fcgi?db=nucleotide&val=AK100609) | 1 |
| *OsGELP103* | LOC_Os10g25340.1 | n/a | 0 |
| *OsGELP104* | LOC_Os10g25380.1 | n/a | 0 |
| *OsGELP105* | LOC_Os10g25400.1 | [AK108245](http://www.ncbi.nlm.nih.gov/entrez/viewer.fcgi?db=nucleotide&val=AK108245) | 5 |
| *OsGELP106* | LOC_Os10g25420.1 | [AK110763](http://www.ncbi.nlm.nih.gov/entrez/viewer.fcgi?db=nucleotide&val=AK110763) | 2 |
| *OsGELP107* | LOC_Os10g30290.1 | [AK065583](http://www.ncbi.nlm.nih.gov/entrez/viewer.fcgi?db=nucleotide&val=AK065583) | 0 |
| *OsGELP108* | LOC_Os10g32580.1 | [AK108201](http://www.ncbi.nlm.nih.gov/entrez/viewer.fcgi?db=nucleotide&val=AK108201) | 33 |
| *OsGEL109* | LOC_Os10g33690.1 | [AK060164](http://www.ncbi.nlm.nih.gov/entrez/viewer.fcgi?db=nucleotide&val=AK060164) [AK070445](http://www.ncbi.nlm.nih.gov/entrez/viewer.fcgi?db=nucleotide&val=AK070445) [AK121743](http://www.ncbi.nlm.nih.gov/entrez/viewer.fcgi?db=nucleotide&val=AK121743) | 23 |
| *OsGELP110* | LOC_Os11g03520.1 | n/a | 2 |
| *OsGELP111* | LOC_Os11g31940.1 | [AK108542](http://www.ncbi.nlm.nih.gov/entrez/viewer.fcgi?db=nucleotide&val=AK108542) | 6 |
| *OsGELP112* | LOC_Os11g48070.1 | [AB110185](http://www.ncbi.nlm.nih.gov/entrez/viewer.fcgi?db=nucleotide&val=AB110185) [AK062013](http://www.ncbi.nlm.nih.gov/entrez/viewer.fcgi?db=nucleotide&val=AK062013) [AK067429](http://www.ncbi.nlm.nih.gov/entrez/viewer.fcgi?db=nucleotide&val=AK067429) [AK109413](http://www.ncbi.nlm.nih.gov/entrez/viewer.fcgi?db=nucleotide&val=AK109413) | 37 |
| *OsGELP113* | LOC_Os12g17570.1 | [AK063071](http://www.ncbi.nlm.nih.gov/entrez/viewer.fcgi?db=nucleotide&val=AK063071) | 4 |
| *OsGELP114a* | LOC_Os12g37910.1 | n/a | 1 |
| *OsGELP114b* | LOC_Os12g37910.2 | [AK060201](http://www.ncbi.nlm.nih.gov/entrez/viewer.fcgi?db=nucleotide&val=AK060201) [AK070277](http://www.ncbi.nlm.nih.gov/entrez/viewer.fcgi?db=nucleotide&val=AK070277) [AK071697](http://www.ncbi.nlm.nih.gov/entrez/viewer.fcgi?db=nucleotide&val=AK071697) | 59 |
