# Supplementary material for: Multifunctionality and diversity of GDSL esterase/lipase gene family in rice (Oryza sativa L. japonica) genome: new insights from bioinformatics analysis
Source: BMC Genomics. 2012 Jul 15;13:309. doi: 10.1186/1471-2164-13-309 (PMC3412167; doi:10.1186/1471-2164-13-309)

**Additional file 3.** The pattern of the *OsGELP* gene clusters on rice chromosomes.

A.

| **Cluster #** | **Clusters’ structure and order of *OsGELP* genes** |
| --- | --- |
| **Cluster I** | *OsGELP*2, Hyp*, Hyp, *OsGELP3*, *4*, Ser Carboxylase, Trans**, Hyp, *OsGELP5*, Hyp, *OsGELP6*, Trans, *OsGELP7*, *8*, *9*, *10*, Hyp, Hyp, *OsGELP11* |
| **Cluster II** | *OsGELP12*, Exp***, RTrans****, RTrans, RTrans, Hyp, *OsGELP13* |
| **Cluster III** | *OsGELP14*, Hyp, Os*GELP15* |
| **Cluster IV** | *OsGELP18*, *19*, Trans, Trans, *OsGELP20*, Exp, Exp, *OsGELP21*, Exp, *OsGELP22*, *23* |
| **Cluster V** | *OsGELP31*, *32* |
| **Cluster VI** | *OsGELP35*, RTrans, RTrans, *OsGELP36*, Hyp, Hyp, Hyp, Hyp, *OsGELP37* |
| **Cluster VII** | *OsGELP41*, *42* |
| **Cluster VIII** | *OsGELP47*, *48*, Hyp, *OsGELP49*, *50* |
| **Cluster IX** | Small GDSL protein, Exp, Trans, Hyp, *OsGELP51*, hypothetic small GDSL lipase protein, Hyp, RTrans, Trans, Hyp, *OsGELP52* |
| **Cluster X** | *OsGELP*61, 62 |
| **Cluster XI** | *OsGELP63*, Hyp, Hyp, RTrans, *OsGELP64*, Hyp, *OsGELP65* |
| **Cluster XII** | *OsGELP69*, *70*, *71*, *72* |
| **Cluster XIII** | *OsGELP76*, Hyp, *OsGELP77*, *78* |
| **Cluster XIV** | *OsGELP84*, Hyp, RTrans, Hyp, *OsGELP85* |
| **Cluster XV** | *OsGELP89*, *90* |
| **Cluster XVI** | *OsGELP* *91*, *92* |
| **Cluster XVII** | *OsGELP* *103*, Hyp, Signal Peptidase Prot, *OsGELP104*, *105*, Hyp, *OsGELP106* |

***Hyp** - Hypothetical protein;

****Trans** - Transposon protein;

*****Exp** - Expressed protein;

******Rtrans** - Retrotransposon protein;

B.


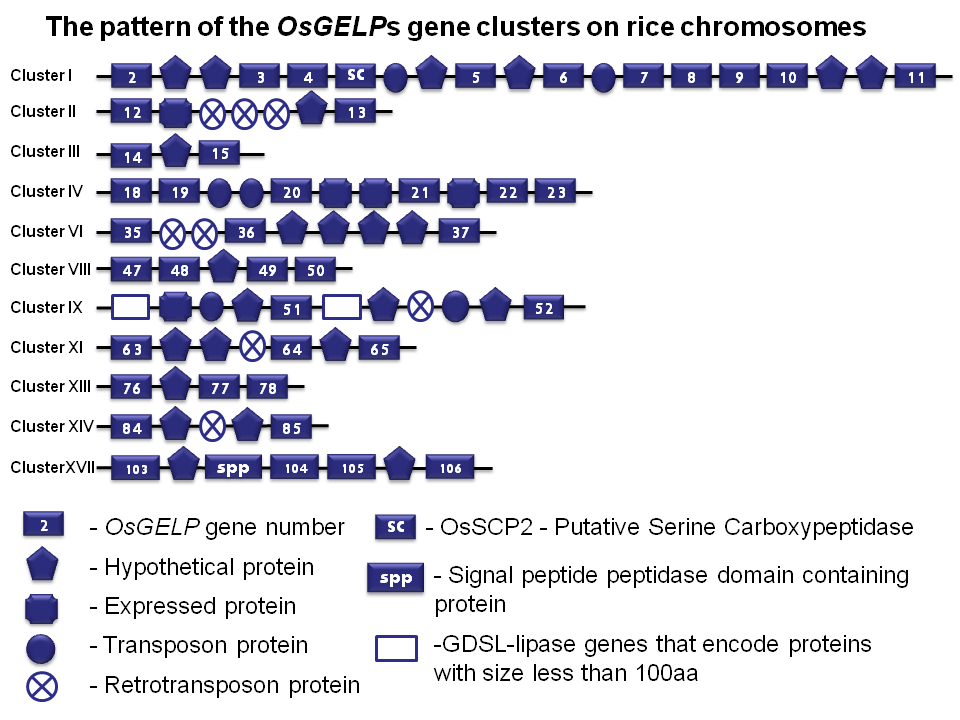

Supplement: Additional file 3 — Pattern of the OsGELP gene clusters on rice chromosomes. (A) The order and clusters’ structures of 54 OsGELP genes on rice chromosomes. (B) The pattern of the OsGELP gene clusters on rice chromosomes, which are interrupted by unrelated genes. [file 1471-2164-13-309-S3.doc]
