# Supplementary material for: Multifunctionality and diversity of GDSL esterase/lipase gene family in rice (Oryza sativa L. japonica) genome: new insights from bioinformatics analysis
Source: BMC Genomics. 2012 Jul 15;13:309. doi: 10.1186/1471-2164-13-309 (PMC3412167; doi:10.1186/1471-2164-13-309)
Supplement: Additional file 4 — The OsGELP genes present on duplicated chromosomal segments of rice O. sativa L. ssp. japonica. The segmental duplicated of the OsGELP genes, with their BLASTP E-value, locus ID, and chromosome coordinates, are present according to the RGAP Segmental Genome Duplication of Rice, with the maximal length distance permitted between collinear gene pairs of 500 kb. [file 1471-2164-13-309-S4.doc]

**Additional file 4.** The *OsGELP* genes present on duplicated chromosomal segments of rice *Oryza sativa* L. ssp. japonica.

| **Gene Name** | **Chromosome** | **CDS Coordinates (5'-3')** | **Gene Name** | **Chromosome** | **CDS Coordinates (5'-3')** | **BLASTP E-value** |
| --- | --- | --- | --- | --- | --- | --- |
| ***OsGELP2*** | 1 | 6266560-6269528 | ***OsGELP85*** | 6 | 19868946-19865402 | 4.300000e-116 |
| ***OsGELP2*** | 1 | 6266560-6269528 | ***OsGELP63*** | 5 | 6787695-6791095 | 4.200000e-157 |
| ***OsGELP3*** | 1 | 6277670-6282247 | ***OsGELP33*** | 2 | 8501883-8504704 | 1.900000e-131 |
| ***OsGELP9*** | 1 | 6360526-6362457 | ***OsGELP64*** | 5 | 6824594-6826885 | 5.900000e-103 |
| ***OsGELP9*** | 1 | 6360526-6362457 | ***OsGELP84*** | 6 | 19841474-19838653 | 1.400000e-92 |
| ***OsGELP14*** | 1 | 12733316-12736715 | ***OsGELP61*** | 5 | 3496645-3493047 | 5.100000e-113 |
| ***OsGELP24*** | 1 | 30360915-30362641 | ***OsGELP25*** | 1 | 31330344-31332023 | 2.100000e-130 |
| ***OsGELP25*** | 1 | 31330344-31332023 | ***OsGELP73*** | 5 | 25622409-25620529 | 2.100000e-146 |
| ***OsGELP27*** | 1 | 35604706-35606486 | ***OsGELP68*** | 5 | 22931853-22930228 | 5.600000e-107 |
| ***OsGELP32*** | 2 | 4944756-4948239 | ***OsGELP87*** | 6 | 25875287-25869456 | 6.800000e-134 |
| ***OsGELP33*** | 2 | 8501883-8504704 | ***OsGELP84*** | 6 | 19841474-19838653 | 6.300000e-154 |
| ***OsGELP40*** | 2 | 24510607-24512161 | ***OsGELP45*** | 2 | 34963314-34962098 | 1.600000e-132 |
| ***OsGELP40*** | 2 | 24510607-24512161 | ***OsGELP56*** | 4 | 25183736-25186581 | 1.500000e-145 |
| ***OsGELP41*** | 2 | 27152112-27150720 | ***OsGELP57*** | 4 | 27939959-27938494 | 2.100000e-82 |
| ***OsGELP42*** | 2 | 27156810-27152805 | ***OsGELP89*** | 6 | 30829225-30827543 | 6.200000e-76 |
| ***OsGELP43*** | 2 | 30550383-30548761 | ***OsGELP82*** | 6 | 8235961-8237505 | 3.600000e-60 |
| ***OsGELP49*** | 3 | 14288310-14291772 | ***OsGELP93*** | 7 | 26728948-26726714 | 6.200000e-92 |
| ***OsGELP57*** | 4 | 27939959-27938494 | ***OsGELP89*** | 6 | 30829225-30827543 | 1.000000e-59 |
