# Supplementary material for: Multifunctionality and diversity of GDSL esterase/lipase gene family in rice (Oryza sativa L. japonica) genome: new insights from bioinformatics analysis
Source: BMC Genomics. 2012 Jul 15;13:309. doi: 10.1186/1471-2164-13-309 (PMC3412167; doi:10.1186/1471-2164-13-309)
Supplement: Additional file 5 — The OsGELP genes resulting from duplications after the eudicots-monocots split, and preceding the sorghum and rice speciation. Such OsGELP genes with their gene names and chromosome locations are presented. [file 1471-2164-13-309-S5.doc]

**Additional file 5.** The *OsGELP* genes resulting from duplications after the eudicots-monocots split, and preceding the rice and sorghum speciation.

| **Gene Name** | **Chromosome** | **Predicted paralogs** | | **Classes of paralogs with respect to speciation events** |
| --- | --- | --- | --- | --- |
|  |  | **Gene Name** | **Chromosome** |  |
| ***OsGELP2*** | 1 | ***OsGELP3-5***  ***OsGELP12***  ***OsGELP33***  ***OsGELP63-65***  ***OsGELP77-79***  ***OsGELP84***  ***OsGELP85*** | 1  1  2  5  6  6  6 | Outparalogs |
| ***OsGELP14*** | 1 | ***OsGELP15***  ***OsGELP16***  ***OsGELP61***  ***OsGELP62*** | 1  1  5  5 | Outparalogs |
| ***OsGELP17*** | 1 | ***OsGELP93***  ***OsGELP105***  ***OsGELP106*** | 7  10  10 | Outparalogs |
| ***OsGELP22*** | 1 | ***OsGELP23*** | 1 | Outparalogs |
| ***OsGELP24*** | 1 | ***OsGELP25***  ***OsGELP72*** | 1  5 | Outparalogs |
| ***OsGELP27*** | 1 | ***OsGELP68*** | 5 | Outparalogs |
| ***OsGELP29*** | 2 | ***OsGELP54***  ***OsGELP83***  ***OsGELP100***  ***OsGELP107*** | 3  6  9  10 | Outparalogs |
| ***OsGELP32*** | 2 | ***OsGELP87*** | 6 | Outparalogs |
| ***OsGELP39*** | 2 | ***OsGELP88*** | 6 | Outparalogs |
| ***OsGELP40*** | 2 | ***OsGELP45***  ***OsGELP56***  ***OsGELP96***  ***OsGELP108*** | 2  4  8  10 | Outparalogs |
| ***OsGELP42*** | 2 | ***OsGELP44*** | 2 | Outparalogs |
| ***OsGELP43*** | 2 | ***OsGELP82*** | 6 | Outparalogs |
| ***OsGELP59*** | 4 | ***OsGELP101*** | 9 | Outparalogs |
| ***OsGELP97*** | 9 | ***OsGELP98***  ***OsGELP99*** | 9  9 | Outparalogs |
