# Supplementary figures and images for: Multifunctionality and diversity of GDSL esterase/lipase gene family in rice (Oryza sativa L. japonica) genome: new insights from bioinformatics analysis
Source: BMC Genomics. 2012 Jul 15;13:309. doi: 10.1186/1471-2164-13-309 (PMC3412167; doi:10.1186/1471-2164-13-309)

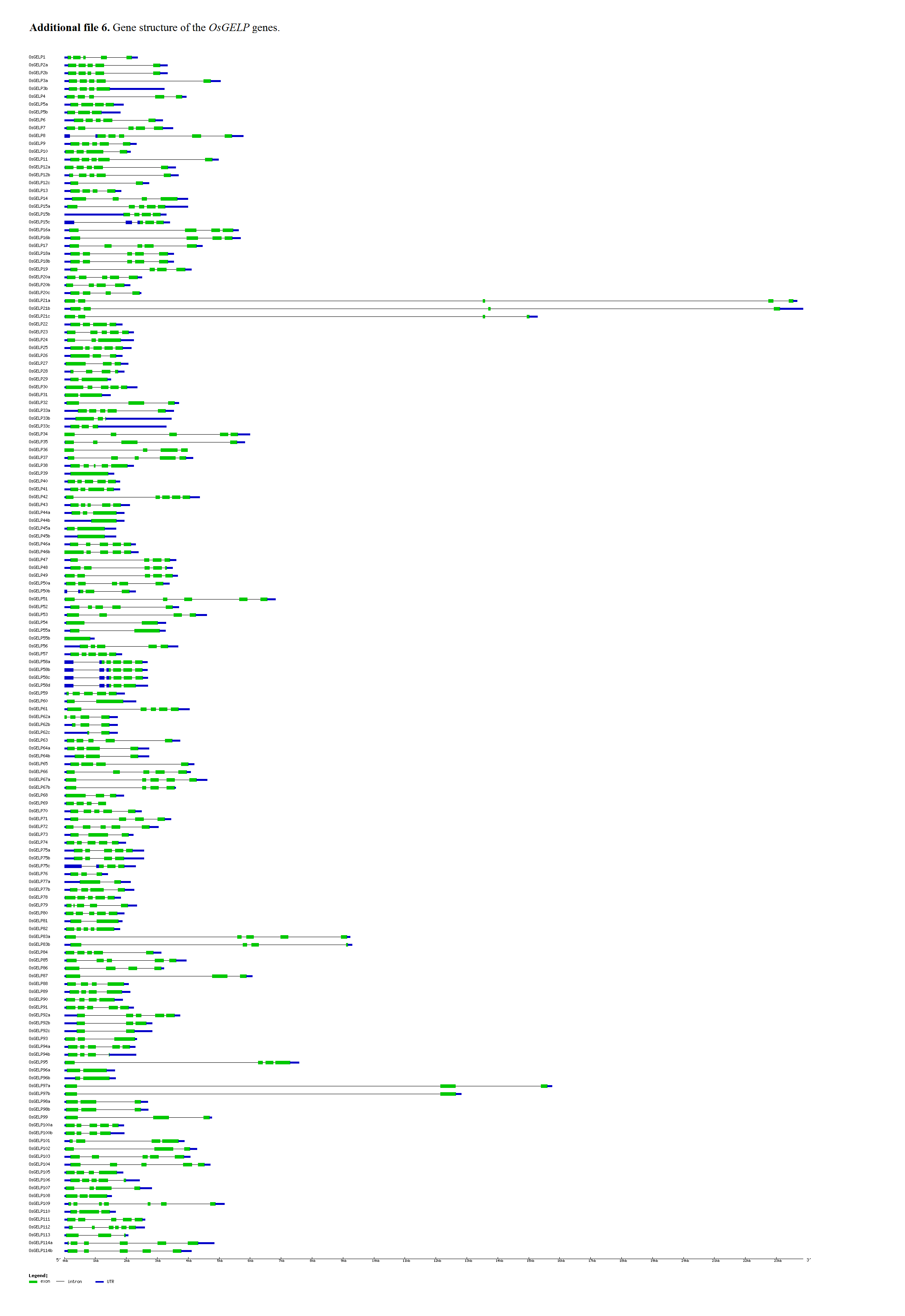

Supplement: Additional file 6 — Gene structure of the OsGELP genes. The exon/intron structures of a total of 153 transcripts (including alternative spliced models) of the 114 OsGELP genes are presented. Green and blue boxes represent exon and UTR regions, respectively, and solid lines indicate intron regions. The length of the boxes and lines are scaled based on the length of genes. [file 1471-2164-13-309-S6.png]
