# Supplementary material for: Multifunctionality and diversity of GDSL esterase/lipase gene family in rice (Oryza sativa L. japonica) genome: new insights from bioinformatics analysis
Source: BMC Genomics. 2012 Jul 15;13:309. doi: 10.1186/1471-2164-13-309 (PMC3412167; doi:10.1186/1471-2164-13-309)
Supplement: Additional file 7 — Chromosomal location and exon/intron number for the OsGELP rice genes. The OsGELP gene names, locus ID, chromosomal location, open reading frame and genomic sequence length, and numbers of exons/introns for each 114 GDSL esterase/lipase genes are given. [file 1471-2164-13-309-S7.doc]

**Additional file 7.** Chromosomal location and exon/intron number for the *OsGELP* rice genes.

| **Gene Name** | **MSU Rice Genome Annotation (Osa1) Release 6.1**  **CDS Coordinates (5'-3')** | **Genomic sequence length** | **ORF**  **Length (bp)** | **Number of Exons** | **Number of Introns** |
| --- | --- | --- | --- | --- | --- |
| ***OsGELP1*** | 6243774 – 6241710 | 2475 | 717 | 5 | 4 |
| ***OsGELP2a***  ***OsGELP2b*** | 6266560 – 6269528 | 3464 | 1101  1050 | 5  5 | 4  4 |
| ***OsGELP3a***  ***OsGELP3b*** | 6277670 – 6282247 | 5160 | 1119  1032 | 5  5 | 4  4 |
| ***OsGELP4*** | 6287428 – 6291165 | 3939 | 1095 | 5 | 4 |
| ***OsGELP5a***  ***OsGELP5b*** | 6303898 – 6305293 | 1807 | 1131  915 | 4  3 | 3  2 |
| ***OsGELP6*** | 6318441 – 6321064 | 3345 | 1137 | 5 | 4 |
| ***OsGELP7*** | 6339906 – 6343019 | 3510 | 1194 | 5 | 4 |
| ***OsGELP8*** | 6352634 – 6356998 | 5778 | 1161 | 6 | 5 |
| ***OsGELP9*** | 6360526 – 6362457 | 1932 | 1149 | 5 | 4 |
| ***OsGELP10*** | 6364063 – 6366057 | 2132 | 1245 | 4 | 3 |
| ***OsGELP11*** | 6376261 – 6380837 | 4777 | 1230 | 5 | 4 |
| ***OsGELP12a***  ***OsGELP12b***  ***OsGELP12b*** | 6727526 – 6724200 | 3688 | 1140  981  450 | 5  5  2 | 4  4  1 |
| ***OsGELP13*** | 6764888 – 6763450 | 1439 | 915 | 4 | 3 |
| ***OsGELP14*** | 12733316 – 12736715 | 4015 | 1311 | 4 | 3 |
| ***OsGELP15a***  ***OsGELP15b***  ***OsGELP15c*** | 2741124 – 12744281 | 3991 | 1155  864  612 | 5  4  5 | 4  3  4 |
| ***OsGELP16a***  ***OsGELP16b*** | 12803705 – 2809004 | 5646 | 1254  1254 | 4  4 | 3  3 |
| ***OsGELP17*** | 24311777 – 24315872 | 4953 | 1227 | 5 | 4 |
| ***OsGELP18a***  ***OsGELP18b*** | 26197070 – 26200211 | 3651 | 1221  1221 | 5  5 | 4  4 |
| ***OsGELP19*** | 26202171 – 26205872 | 3702 | 933 | 4 | 3 |
| ***OsGELP20a***  ***OsGELP20b***  ***OsGELP20c*** | 26221652 – 6223934 | 2508 | 1203  924  879 | 5  4  4 | 4  3  3 |
| ***OsGELP21a***  ***OsGELP21b***  ***OsGELP21c*** | 26248363 – 26271885 | 24799 | 888  783  669 | 6  5  4 | 5  4  3 |
| ***OsGELP22*** | 26285397 – 26286866 | 1470 | 1143 | 4 | 3 |
| ***OsGELP23*** | 26289190 – 26291183 | 2240 | 1113 | 5 | 4 |
| ***OsGELP24*** | 30360915 – 30362641 | 2347 | 1092 | 3 | 2 |
| ***OsGELP25*** | 31330344 – 31332023 | 2306 | 1236 | 5 | 4 |
| ***OsGELP26*** | 35416131 – 35414663 | 1879 | 1053 | 3 | 2 |
| ***OsGELP27*** | 35604706 – 35606486 | 2212 | 1095 | 3 | 2 |
| ***OsGELP28*** | 42256146 – 42254611 | 1536 | 627 | 4 | 3 |
| ***OsGELP29*** | 78232 – 79417 | 1410 | 1080 | 2 | 1 |
| ***OsGELP30*** | 548008 – 54603 | 2574 | 1362 | 5 | 4 |
| ***OsGELP31*** | 4942772 – 4943973 | 1494 | 1134 | 2 | 1 |
| ***OsGELP32*** | 4944756 – 4948239 | 3727 | 1095 | 3 | 2 |
| ***OsGELP33a***  ***OsGELP33b***  ***OsGELP33c*** | 8501883 – 8504704 | 3536 | 1149  735  654 | 5  3  3 | 4  2  2 |
| ***OsGELP34*** | 11009375 – 1014972 | 5604 | 1200 | 5 | 4 |
| ***OsGELP35*** | 11071707 – 11077265 | 5827 | 1128 | 4 | 3 |
| ***OsGELP36*** | 11093807 – 11097790 | 3984 | 1182 | 4 | 3 |
| ***OsGELP37*** | 11116284 – 11120101 | 4158 | 1230 | 5 | 4 |
| ***OsGELP38*** | 23658590 – 23656750 | 1841 | 1221 | 5 | 4 |
| ***OsGELP39*** | 23888600 – 23887389 | 1212 | 1212 | 1 | 0 |
| ***OsGELP40*** | 24510607 – 24512161 | 1827 | 1104 | 5 | 4 |
| ***OsGELP41*** | 27152112 – 27150720 | 1393 | 1092 | 4 | 3 |
| ***OsGELP42*** | 27156810 – 27152805 | 4419 | 1089 | 5 | 4 |
| ***OsGELP43*** | 30550383 – 30548761 | 1916 | 969 | 5 | 4 |
| ***OsGELP44a***  ***OsGELP44b*** | 30961491 – 30960046 | 2018 | 1152  813 | 3  1 | 2  n/a |
| ***OsGELP45a***  ***OsGELP45b*** | 34963314 – 34962098 | 1670 | 1110  879 | 2  1 | 1  n/a |
| ***OsGELP46a***  ***OsGELP46b*** | 11058753 – 11060718 | 2573 | 1104  846 | 5  5 | 4  4 |
| ***OsGELP47*** | 14267414 – 14270626 | 3213 | 831 | 4 | 3 |
| ***OsGELP48*** | 14275164 – 14278267 | 3104 | 1020 | 5 | 4 |
| ***OsGELP49*** | 14288310 – 14291772 | 3725 | 1164 | 5 | 4 |
| ***OsGELP50a***  ***OsGELP50b*** | 14293249 – 14296381 | 3392 | 1179  621 | 5  4 | 4  3 |
| ***OsGELP51*** | 21312011 – 21305472 | 6863 | 1140 | 5 | 4 |
| ***OsGELP52*** | 21352028 – 21355323 | 3296 | 1104 | 5 | 4 |
| ***OsGELP53*** | 27245072 – 27249239 | 4632 | 1074 | 4 | 3 |
| ***OsGELP54*** | 35499639 – 35496680 | 3768 | 1104 | 2 | 1 |
| ***OsGELP55*** | 36252071 – 36249186 | 3485 | 1098 | 2 | 1 |
| ***OsGELP56*** | 25183736 – 25186581 | 3743 | 1107 | 5 | 4 |
| ***OsGELP57*** | 27939959 – 27938494 | 1466 | 1104 | 5 | 4 |
| ***OsGELP58a***  ***OsGELP58b***  ***OsGELP58c***  ***OsGELP58d*** | 28926182 – 28927513 | 2728 | 969  969  807  693 | 6  6  6  5 | 5  5  5  4 |
| ***OsGELP59*** | 32949070 – 32947460 | 1946 | 1068 | 5 | 4 |
| ***OsGELP60*** | 1931960 – 1933764 | 2309 | 1098 | 2 | 1 |
| ***OsGELP61*** | 3496645 – 3493047 | 4144 | 1296 | 5 | 4 |
| ***OsGELP62a***  ***OsGELP62b***  ***OsGELP62c*** | 3500554 – 3499350 | 1730 | 636  636  306 | 4  3  2 | 3  2  1 |
| ***OsGELP63*** | 6787695 – 6791095 | 3916 | 1086 | 5 | 4 |
| ***OsGELP64a***  ***OsGELP64b*** | 6824594 – 6826885 | 2799 | 1113  948 | 4  3 | 3  2 |
| ***OsGELP65*** | 6838735 – 6842533 | 3958 | 1143 | 4 | 3 |
| ***OsGELP66*** | 19440658 – 19444547 | 4477 | 1167 | 5 | 4 |
| ***OsGELP67a***  ***OsGELP67b*** | 20523728 – 20527949 | 4711 | 1182  963 | 5  4 | 4  3 |
| ***OsGELP68*** | 22931853 – 22930228 | 1933 | 1083 | 3 | 2 |
| ***OsGELP69*** | 24961428 – 24960144 | 1285 | 855 | 4 | 3 |
| ***OsGELP70*** | 24964651 – 24962556 | 2096 | 1116 | 5 | 4 |
| ***OsGELP71*** | 24970257 – 24967215 | 3043 | 966 | 4 | 3 |
| ***OsGELP72*** | 24976320 – 24973629 | 3036 | 1128 | 5 | 4 |
| ***OsGELP73*** | 25622409 – 25620529 | 2243 | 1098 | 3 | 2 |
| ***OsGELP74*** | 2514840 – 2516505 | 2181 | 1056 | 5 | 4 |
| ***OsGELP75a***  ***OsGELP75b***  ***OsGELP75c*** | 2546004 – 2547888 | 2570 | 1083  909  597 | 5  4  4 | 4  3  3 |
| ***OsGELP76*** | 2883915 – 2882907 | 1009 | 570 | 3 | 2 |
| ***OsGELP77a***  ***OsGELP77b*** | 2892759 – 2894523 | 2289 | 864  1083 | 2  4 | 1  3 |
| ***OsGELP78*** | 2897811 – 2899406 | 1834 | 1215 | 5 | 4 |
| ***OsGELP79*** | 2909412 – 2907342 | 2893 | 1026 | 5 | 4 |
| ***OsGELP80*** | 3057968 – 3056311 | 2002 | 1140 | 5 | 4 |
| ***OsGELP81*** | 6735086 – 6736679 | 1751 | 1041 | 2 | 1 |
| ***OsGELP82*** | 8235961 – 8237505 | 1798 | 1173 | 5 | 4 |
| ***OsGELP83a***  ***OsGELP83b*** | 14280577 – 14289672 | 9418 | 1137  741 | 5  4 | 4  3 |
| ***OsGELP84*** | 19841474 – 19838653 | 3522 | 1128 | 5 | 4 |
| ***OsGELP85*** | 19868946 – 19865402 | 3937 | 1188 | 5 | 4 |
| ***OsGELP86*** | 21446806 – 21449900 | 3360 | 1224 | 4 | 3 |
| ***OsGELP87*** | 25875287 – 25869456 | 6238 | 1149 | 3 | 2 |
| ***OsGELP88*** | 28982343 – 28980518 | 2313 | 1140 | 4 | 3 |
| ***OsGELP89*** | 30829225 – 30827543 | 2164 | 1146 | 4 | 3 |
| ***OsGELP90*** | 30838904 – 30837339 | 1964 | 1167 | 4 | 3 |
| ***OsGELP91*** | 23815405 – 23813388 | 2325 | 1221 | 5 | 4 |
| ***OsGELP92a***  ***OsGELP92b***  ***OsGELP92c*** | 23822731 – 23819574 | 4332 | 1176  795  516 | 5  3  2 | 4  2  1 |
| ***OsGELP93*** | 26728948 – 26726714 | 2559 | 1176 | 3 | 2 |
| ***OsGELP94a***  ***OsGELP94b*** | 28223782 – 28221763 | 2344 | 1155  678 | 5  4 | 4  3 |
| ***OsGELP95*** | 685310 – 692575 | 7593 | 1146 | 4 | 3 |
| ***OsGELP96a***  ***OsGELP96b*** | 28343746 – 28345020 | 2421 | 1164  987 | 2  2 | 1  1 |
| ***OsGELP97a***  ***OsGELP97b*** | 2457950 – 2473516 | 15759 | 1056  867 | 3  2 | 2  1 |
| ***OsGELP98a***  ***OsGELP98b*** | 2500732 – 2503145 | 2755 | 1059  1035 | 3  3 | 2  2 |
| ***OsGELP99*** | 3585006 – 3580358 | 4761 | 1071 | 3 | 2 |
| ***OsGELP100a***  ***OsGELP100b*** | 21272059 – 21270362 | 1937 | 1101  975 | 5  4 | 4  3 |
| ***OsGELP101*** | 22674792 – 22671281 | 4018 | 1149 | 4 | 3 |
| ***O GELP102*** | 2491596 – 2495623 | 4277 | 1050 | 3 | 2 |
| ***OsGELP103*** | 13033585 – 13037252 | 3699 | 1230 | 5 | 4 |
| ***OsGELP104*** | 13051994 – 13056313 | 4320 | 1161 | 5 | 4 |
| ***OsGELP105*** | 13060267 – 13061895 | 1896 | 1206 | 4 | 3 |
| ***OsGELP106*** | 13071169 – 13072956 | 2233 | 1017 | 5 | 4 |
| ***OsGELP107*** | 15665635 – 15663236 | 2827 | 1077 | 4 | 3 |
| ***O GELP108*** | 16999740 – 17001069 | 1704 | 1173 | 3 | 2 |
| ***OsGELP109*** | 17695770 – 17700510 | 5168 | 813 | 7 | 6 |
| ***OsGELP110*** | 1359545 – 1358284 | 1357 | 1089 | 3 | 2 |
| ***OsGELP111*** | 18303979 – 18306396 | 2607 | 1122 | 5 | 4 |
| ***OsGELP112*** | 28460029 – 28462191 | 2820 | 783 | 6 | 5 |
| ***OsGELP113*** | 10069492 – 10067571 | 2442 | 846 | 2 | 1 |
| ***OsGELP114a***  ***OsGELP114b*** | 23265540 – 23261865 | 4344 | 1176  1209 | 6  5 | 5  4 |
