# Supplementary material for: Multifunctionality and diversity of GDSL esterase/lipase gene family in rice (Oryza sativa L. japonica) genome: new insights from bioinformatics analysis
Source: BMC Genomics. 2012 Jul 15;13:309. doi: 10.1186/1471-2164-13-309 (PMC3412167; doi:10.1186/1471-2164-13-309)
Supplement: Additional file 8 — Identification of the repetitive DNA sequences within the OsGELP rice gene family. Diverse types of repetitive sequences with names, length (bp), and their positions and numbers for the 71 OsGELP genes are shown. The list of the repetitive DNA sequences present in the OsGELP genes is displayed in the order of their appearance from 5′- to 3′-end. [file 1471-2164-13-309-S8.doc]

**Additional file 8.** **Identification of** the repetitive DNA sequences within *OsGELP* rice gene family.

| **Gene Name** | **List of repetitive DNA sequences in the *OsGELP* genes** | **Total number of repetitive DNA sequences in the *OsGELP* genes** | **RepeatMasker load_ID** | **Repetitive DNA sequences type** | **Position in the *OsGELP* genes** | **Length (bp)** |
| --- | --- | --- | --- | --- | --- | --- |
| ***OsGELP2*** | putative MITE, Wanderer-like | **1** | **repeat_230232** | Repeat | 4^th^ intron | 199 |
| ***OsGELP3*** | putative retrotransposon, Ty3-gypsy-like  putative MITE, Ditto-like  putative retrotransposon  SINE-like putative MITE, p-SINE1-like | **4** | **repeat_238110**  **repeat_209693**  **repeat_224100**  **repeat_228155** | Repeat  Repeat  Repeat | 4^th^ intron | 40  248  56  206 |
| ***OsGELP4*** | putative MITE, MITE-adh, type K-like | **1** | **repeat_227493** | Repeat | 4^th^ intron | 183 |
| ***OsGELP5*** | unclassified transposon | **1** | **repeat_213817** | Repeat | 3’ UTR | 324 |
| ***OsGELP7*** | putative MITE, MITE-adh, type H-like  putative MITE, MITE-adh, type K-like | **2** | **repeat_239677**  **repeat_227494** | Repeat  Repeat | 2^nd^ intron  4^th^ intron | 295  161 |
| ***OsGELP8*** | putative MITE, MITE-adh, type B-like  putative MITE, p-SINE1-like  putative MITE  Stowaway-lik  unclassified transposon  unclassified repetitive sequence  unclassified retrotransposon  putative MITE, Pangrangja-like  putative MITE, MITE-adh, type A-like  putative MITE, MITE-adh, type A-like  putative MITE, Pangrangja-like  putative MITE, p-SINE1-like  putative MITE, MITE-adh, type I-like  unclassified transposon | **13** | **repeat_225278**  **repeat_228158**  **repeat_226848**  **repeat_213828**  **repeat_218939**  **repeat_231164**  **repeat_209074**  **repeat_223106**  **repeat_223105**  **repeat_209073**  **repeat_228159**  **repeat_211914**  **repeat_213829** | Repeat  Repeat  Repeat  Repeat | 5’ UTR  4^th^ intron  5^th^ intron  3’UTR | 169  87  102  205  155  359  158  153  132  96  124  195  138 |
| ***OsGELP10*** | putative retrotransposon, SINE-like | **1** | **repeat_224102** | Repeat | 3^rd^ intron | 64 |
| ***OsGELP11*** | putative MITE, MITE-adh-8-like  putative MITE, MITE-adh, type A-like  unclassified transposable element  putative MITE, Micron-like  unclassified transposon  putative MITE, MITE-adh, type D-like | **6** | **repeat_238880**  **repeat_223108**  **repeat_212595**  **repeat_229243**  **repeat_213838** | Repeat | 4^th^ intron | 160  163  65  479  164  73 |
| ***OsGELP12*** | putative MITE, MITE-adh-5-like  putative MITE, MITE-adh, type B-like  unclassified transposon | **3** | **repeat_222644**  **repeat_225299**  **repeat_213869** | Repeat | 5’ UTR  4^th^ intron | 372  177  622 |
| ***OsGELP13*** | putative MITE, MITE-adh-3-like | **1** | **repeat_226785** | Repeat | 3^rd^ intron | 217 |
| ***OsGELP14*** | putative MITE, MITE-adh, type B-like | **1** | **repeat_225561** | Repeat | 2^nd^ intron | 244 |
| ***OsGELP16*** | putative retrotransposon, SINE-like  putative MITE, Castaway-like  putative MITE, MITE-adh-5-like  unclassified transposon  putative MITE, MITE-adh-5-like  putative retrotransposon, SINE-like  putative MITE, Tourist-like | **7** | **repeat_224251**  **repeat_211430**  **repeat_222700**  **repeat_214765**  **repeat_222701**  **repeat_224252**  **repeat_235902** | Repeat | 1^st^ intron  2^nd^ intron | 92  278  80  236  275  220  322 |
| ***OsGELP18*** | putative MITE, Stowaway-like  putative transposon, CACTA, En/Spm-like | **2** | **repeat_226938**  **repeat_239462** | Repeat | 2^nd^ intron | 65  88 |
| ***OsGELP19*** | unclassified repetitive sequence  unclassified repetitive sequence  unclassified repetitive sequence  unclassified repetitive sequence | **4** | **repeat_219837**  **repeat_219838**  **repeat_219839**  **repeat_219840** | Repeat | 1^st^ intron | 85  65  68  66 |
| ***OsGELP21*** | mcgill_packs**_**pack_mule  unclassified transposon  unclassified transposon  unclassified transposon  putative MITE, Ditto-like  putative MITE, Ditto-like  unclassified retrotransposon  unclassified repetitive sequence  putative retrotransposon, Ty3-gypsy-like  unclassified transposon  unclassified retrotransposon  putative retrotransposon, Ty3-gypsy-like  putative retrotransposon, Ty3-gypsy-like  putative retrotransposon, Ty3-gypsy-like  unclassified retrotransposon  telomere-like  putative MITE, MITE-adh-5-like  putative MITE, Ditto-like  putative MITE, Ditto-like  putative retrotransposon, Ty3-gypsy-like  putative retrotransposon, Ty3-gypsy-like  putative MITE, Micron-like  unclassified retrotransposon | **23** | **mcgill_packs_8598**  **repeat_216409**  **repeat_216410**  **repeat_216411**  **repeat_210055**  **repeat_210056**  **repeat_233631**  **repeat_219844**  **repeat_238669**  **repeat_216412**  **repeat_233632**  **repeat_238670**  **repeat_238671**  **repeat_238672**  **repeat_233633**  **repeat_238021**  **repeat_222789**  **repeat_210057**  **repeat_210058**  **repeat_238673**  **repeat_238674**  **repeat_229326**  **repeat_233634** | MCGILL_PACKS  Repeat | 1^st^ intron  1^st^ intron  1^st^ intron, 3^rd^ exon, 2^nd^ intron  2^nd^ intron  3^rd^ intron  3’ UTR | 518  243  197  192  74  41  1063  281  377  3895  279  471  58  2157  201  61  351  255  80  1037  208  165  425 |
| ***OsGELP27*** | putative MITE, ID-3-like | **1** | **repeat_221183** | Repeat | 3’UTR | 259 |
| ***OsGELP28*** | putative transposon, mutator (MULE)-like  putative MITE, MITE-adh, type I-like | **2** | **repeat_222298**  **repeat_212563** | Repeat | 2^nd^ intron | 214  188 |
| ***OsGELP31*** | putative retrotransposon, SINE-like | **1** | **repeat_251896** | Repeat | 3’UTR | 93 |
| ***OsGELP32*** | putative MITE, MITE-adh, type A-like  putative MITE, MITE-adh-2-like  putative MITE, Pangrangja-like  putative MITE, MITE-adh, type A-like | **4** | **repeat_251166**  **repeat_249607**  **repeat_240119**  **repeat_251167** | Repeat | 1^st^ intron  2^nd^ intron | 245  226  140  170 |
| ***OsGELP35*** | putative MITE, Tourist-like  unclassified transposon  unclassified transposable element  putative MITE, MITE-adh, type D-like  putative retrotransposon, SINE-like  unclassified retrotransposon  putative retrotransposon, Ty3-gypsy-like  unclassified retrotransposon  putative retrotransposon, Ty3-gypsy-like | **9** | **repeat_261726**  **repeat_244533**  **repeat_242897**  **repeat_262790**  **repeat_252027**  **repeat_258432**  **repeat_263772**  **repeat_258433**  **repeat_263773** | Repeat | 1^st^ intron  2^nd^ intron  3^rd^ intron | 266  119  81  97  93  166  360  290  49 |
| ***OsGELP36*** | putative MITE, MITE-adh, type B-like  putative MITE, Gaijin/Gaigin-like  putative MITE, Tourist-like  putative MITE, MITE-adh, type J-like  unclassified transposon  putative MITE, Wanderer-like  putative MITE, MITE-adh-5-like  putative MITE, Wanderer-like  putative transposon, mutator (MULE)-like | **9** | **repeat_253023**  **repeat_241389**  **repeat_261727**  **repeat_261077**  **repeat_244534**  **repeat_257138**  **repeat_250859**  **repeat_257139**  **repeat_250325** | Repeat | 1^st^ intron  2^nd^ Intron | 231  61  316  146  173  191  243  160  219 |
| ***OsGELP38*** | putative retrotransposon, SINE-like | **1** | **repeat_252258** | Repeat | 2^nd^ intron | 81 |
| ***OsGELP42*** | putative retrotransposon, SINE-like  putative MITE, Ditto-like | **2** | **repeat_252328**  **repeat_240962** | Repeat | 1^st^ intron | 45  258 |
| ***OsGELP44*** | unclassified repetitive sequence | **1** | **repeat_249094** | Repeat | 3’UTR | 201 |
| ***OsGELP47*** | putative MITE, MITE-adh-3-like  putative MITE, MITE-adh-3-like | **2** | **repeat_138654**  **repeat_138655** | Repeat | 1^st^ intron | 123  89 |
| ***OsGELP48*** | putative transposon, CACTA, En/Spm-like  putative transposon, CACTA, En/Spm-like | **2** | **repeat_149124**  **repeat_149125** | Repeat | 2^nd^ intron | 525  403 |
| ***OsGELP49*** | unclassified transposon | **1** | **repeat_129330** | Repeat | 2^nd^ intron | 884 |
| ***OsGELP50*** | putative transposon, CACTA, En/Spm-like  putative MITE, MITE-adh-4-like  putative MITE, MITE-adh-4-like  putative retrotransposon, SINE-like | **4** | **repeat_149127**  **repeat_139623**  **repeat_139624**  **repeat_136513** | Repeat | 4^th^ intron | 143  122  73  138 |
| ***OsGELP51*** | putative MITE, MITE-adh, type K-like  putative MITE, MITE-adh, type B-like  putative MITE, p-SINE1-like  putative MITE, Ditto-like  unclassified transposon  putative MITE, p-SINE1-like  putative MITE, MITE-adh, type A-like  putative MITE, MITE-adh, type B-like | **8** | **repeat_139400**  **repeat_137911**  **repeat_140266**  **repeat_124940**  **repeat_130154**  **repeat_140265**  **repeat_135691**  **repeat_137910** | Repeat | 1^st^ intron  3^rd^ intron  4^th^ intron | 187  245  275  35  39  58  183  241 |
| ***OsGELP52*** | unclassified transposon  putative MITE, p-SINE1-like  putative MITE, MITE-adh, type B-like  putative MITE, MITE-adh, type J-like  putative retrotransposon, SINE-like  putative MITE, MITE-adh, type G-like  putative MITE, MITE-adh, type G-like | **7** | **repeat_130156**  **repeat_140270**  **repeat_137914**  **repeat_145758**  **repeat_136641**  **repeat_133838**  **repeat_133839** |  | 1^st^ intron  4^th^ intron | 94  70  457  60  62  197  457 |
| ***OsGELP53*** | putative MITE, MITE-adh, type B-like  unclassified transposon  putative MITE, Wanderer-like | **3** | **repeat_138198**  **repeat_131009**  **repeat_141869** | Repeat | 1^st^ intron | 233  130  131 |
| ***OsGELP54*** | putative MITE, p-SINE1-like  putative MITE, MITE-adh-3-like  putative retrotransposon, SINE-like  putative MITE, Explorer-like | **4** | **repeat_140725**  **repeat_138676**  **repeat_136948**  **repeat_141571** | Repeat | 1^st^ intron | 170  203  113  108 |
| ***OsGELP55*** | unclassified transposon | **1** | **repeat_132092** | Repeat | 3’UTR | 232 |
| ***OsGELP60*** | putative MITE, MITE-adh, type B-like  putative MITE, MITE-adh, type B-like | **2** | **repeat_51970**  **repeat_51971** | Repeat | 1^st^ intron | 239  213 |
| ***OsGELP62*** | putative MITE, MITE-adh, type K-like  putative MITE, Explorer-like | **2** | **repeat_53422**  **repeat_55164** | Repeat | 3^rd^ intron | 177  128 |
| ***OsGELP63*** | unclassified transposon  putative MITE, p-SINE1-like  putative MITE, MITE-adh, type B-like | **3** | **repeat_45242**  **repeat_53972**  **repeat_52202** | Repeat | 3^rd^ intron  4^th^ intron | 175  258  229 |
| ***OsGELP65*** | unclassified transposon  putative MITE, MITE-adh, type A-like  putative MITE, p-SINE1-like  putative MITE, p-SINE1-like  putative MITE, Ditto-like  unclassified transposon  unclassified repetitive sequence  unclassified retrotransposon  putative MITE, Tourist-like | **9** | **repeat_45250**  **repeat_50740**  **repeat_53974**  **repeat_53975**  **repeat_42366**  **repeat_45251**  **repeat_48244**  **repeat_56321**  **repeat_59559** | Repeat | 3^rd^ intron | 57  241  36  166  250  255  59  85  55 |
| ***OsGELP66*** | putative MITE, Stowaway-like  putative retrotransposon, SINE-like | **2** | **repeat_53142**  **repeat_51557** | Repeat | 2^nd^ intron  3’ UTR | 35  104 |
| ***OsGELP67*** | putative MITE, MITE-adh, type M-like  putative MITE, Snap-like | **2** | **repeat_49195**  **repeat_51188** | Repeat | 1^st^ intron  3^rd^ intron | 150  155 |
| ***OsGELP70*** | putative retrotransposon, SINE-like | **1** | **repeat_51659** | Repeat | 1^st^ intron | 73 |
| ***OsGELP71*** | putative MITE, MITE-adh-5-like  putative MITE, MITE-adh, type B-like  putative MITE, Stowaway-like  putative MITE, Micron-like  putative MITE, MITE-adh-10-like  putative retrotransposon, SINE-like  putative MITE, MITE-adh, type M-like | **7** | **repeat_50428**  **repeat_52758**  **repeat_53174**  **repeat_54634**  **repeat_43220**  **repeat_51660**  **repeat_49222** | Repeat | 1^st^ intron  2^nd^ exon 2^nd^ intron  3^rd^ intron | 347  232  104  64  186  58  68 |
| ***OsGELP72*** | unclassified transposon  putative MITE, MITE-adh, type B-like  putative MITE, MITE-adh, type A-like  putative MITE, Gaijin/Gaigin-like  putative MITE, Tourist-like | **5** | **repeat_47437**  **repeat_52761**  **repeat_50896**  **repeat_43064**  **repeat_60052** | Repeat | 2^nd^ intron  4^th^ intron  3’UTR | 174  237  231  48  323 |
| ***OsGELP73*** | putative MITE, Pangrangja-like  putative retrotransposon, SINE-like | **2** | **repeat_42140**  **repeat_51675** | Repeat | 1^st^ intron  2^nd^ intron | 251  176 |
| ***OsGELP75*** | putative MITE, MITE-adh, type D-like  putative MITE, p-SINE1-like  unclassified transposon | **3** | **repeat_104118**  **repeat_96937**  **repeat_86961** | Repeat | 5’UTR  1^st^ intron | 55  99  279 |
| ***OsGELP76*** | putative retrotransposon, SINE-like  putative MITE, Ditto-like | **2** | **repeat_94094**  **repeat_84491** | Repeat | 2^nd^ intron | 56  247 |
| ***OsGELP79*** | putative MITE, Gaijin/Gaigin-like  putative MITE, MITE-adh, type J-like | **2** | **repeat_84975**  **repeat_102801** | Repeat | 3^rd^ intron | 164  110 |
| ***OsGELP81*** | putative MITE, MITE-adh, type B-like  putative MITE, MITE-adh, type A-like | **2** | **repeat_95134**  **repeat_93491** | Repeat | 1^st^ intron | 139  227 |
| ***OsGELP83*** | unclassified transposon  putative MITE, Tourist-like  putative MITE, Tourist-like  putative MITE, Castaway-like  putative retrotransposon, Ty1-copia-like | **5** | **repeat_88539**  **repeat_103532**  **repeat_103533**  **repeat_85655**  **repeat_92372** | Repeat | 1^st^ intron | 257  235  341  197  202 |
| ***OsGELP85*** | putative retrotransposon, SINE-like | **1** | **repeat_94417** | Repeat | 3^rd^ intron | 69 |
| ***OsGELP86*** | putative MITE, MITE-adh, type B-like  unclassified transposon | **2** | **repeat_95603**  **repeat_89235** | Repeat | 1^st^ intron  3’UTR | 234  494 |
| ***OsGELP87*** | putative retrotransposon, SINE-like  putative MITE, MITE-adh, type J-like  putative MITE, MITE-adh, type D-like  putative MITE, MITE-adh-9-like  putative MITE, Tourist-like  unclassified transposon  putative MITE, MITE-adh, type B-like  putative MITE, p-SINE1-like | **8** | **repeat_94554**  **repeat_103010**  **repeat_104690**  **repeat_86552**  **repeat_103835**  **repeat_89861**  **repeat_95804**  **repeat_97588** | Repeat | 1^st^ intron | 117  102  154  122  290  255  235  75 |
| ***OsGELP88*** | putative MITE, MITE-adh, type A-like  putative MITE, MITE-adh, type B-like  putative MITE, Snabo-like | **3** | **repeat_93730**  **repeat_95981**  **repeat_93931** | Repeat | 3^rd^ intron  3’UTR | 253  172  141 |
| ***OsGELP91*** | putative MITE, MITE-adh, type K-like  putative MITE, MITE-adh, type K-like  putative MITE, Explorer-like | **3** | **repeat_199309**  **repeat_199310**  **repeat_200935** | Repeat | 3^rd^ intron | 235  164  143 |
| ***OsGELP92*** | unclassified retrotransposon  putative MITE, Gaijin/Gaigin-like  unclassified repetitive sequence  putative MITE, MITE-adh-5-like | **4** | **repeat_204415**  **repeat_188938**  **repeat_194628**  **repeat_196192** | Repeat | 3^rd^ intron  3’UTR | 153  182  111  257 |
| ***OsGELP93*** | putative MITE, Pangrangja-like  unclassified transposon  putative MITE, Wanderer-like | **3** | **repeat_188049**  **repeat_193358**  **repeat_201314** | Repeat | 2^nd^ intron | 220  162  171 |
| ***OsGELP94*** | unclassified transposon  putative MITE, Pangrangja-like | **2** | **repeat_193553**  **repeat_188055** | Repeat | 3^rd^ intron | 162  248 |
| ***OsGELP95*** | putative MITE, MDM-like  putative MITE, Ditto-like  putative MITE, MITE-adh-5-like  unclassified repetitive sequence  putative MITE, p-SINE1-like  putative MITE, MITE-adh-5-like  putative transposon, CACTA, En/Spm-like  putative MITE, Micron-like  unclassified transposon  putative MITE, MITE-adh, type D-like | **10** | **repeat_62884**  **repeat_63284**  **repeat_71030**  **repeat_68715**  **repeat_74265**  **repeat_71031**  **repeat_82890**  **repeat_75080**  **repeat_65325**  **repeat_80936** | Repeat | 1^st^ intron | 359  43  155  82  69  77  121  82  45  91 |
| ***OsGELP97*** | unclassified retrotransposon  putative MITE, MITE-adh, type D-like  putative MITE, MITE-adh, type D-like  unclassified transposon  putative MITE, MITE-adh, type B-like  putative MITE, MITE-adh, type D-like  unclassified transposon  putative MITE, p-SINE1-like  putative MITE, Wanderer-like  putative centromere sequence, centromere-specific retrotransposon-like  putative MITE, MITE-adh, type A-like  putative MITE, Pangrangja-like  unclassified transposon  unclassified retrotransposon  putative MITE, MITE-adh-11-like  unclassified retrotransposon | **16** | **repeat_117917**  **repeat_121184**  **repeat_121184**  **repeat_121185**  **repeat_109011**  **repeat_114450**  **repeat_121186**  **repeat_109012**  **repeat_116004**  **repeat_117263**  **repeat_116775**  **repeat_113503**  **repeat_106956**  **repeat_117918**  **repeat_115634**  **repeat_117919** | Repeat | 1^st^ intron  2^nd^ intron | 157  133  120  85  154  154  440  183  162  55  215  152  322  155  515  304 |
| ***OsGELP98*** | putative MITE, Susu-like  putative MITE, Kiddo-like | **2** | **repeat_107206**  **repeat_123390** | Repeat | 2^nd^ intron  3’UTR | 259  244 |
| ***OsGELP99*** | unclassified repetitive sequence  unclassified repetitive sequence  putative MITE, Tourist-like  putative MITE, MITE-adh-4-like | **4** | **repeat_111524**  **repeat_111523**  **repeat_120519**  **repeat_115931** | Repeat | 1^st^ Intron  2^nd^ intron  3’UTR | 237  36  217  66 |
| ***OsGELP101*** | putative MITE, Gaijin/Gaigin-like  unclassified transposon  putative MITE, Tourist-like  putative MITE, MITE-adh, type N-like | **4** | **repeat_107965**  **repeat_111423**  **repeat_121133**  **repeat_115345** | Repeat | 2^nd^ Intron  3’UTR | 152  135  349  112 |
| ***OsGELP102*** | unclassified retrotransposon  putative transposon, CACTA, En/Spm-like  putative MITE, Explorer-like  putative MITE, MITE-adh-5-like | **4** | **repeat_181845**  **repeat_186995**  **repeat_181081**  **repeat_177192** | Repeat | 1^st^ intron  2^nd^ intron | 100  352  131  249 |
| ***OsGELP103*** | putative retrotransposon, SINE-like | **1** | **repeat_178124** | Repeat | 1^st^ intron | 220 |
| ***OsGELP104*** | putative MITE, Pangrangja-like  unclassified transposon  putative MITE, Tourist-like  unclassified transposon  putative MITE, Crackle-like  putative MITE, Gaijin/Gaigin-like  putative MITE, MITE-adh, type D-like | **7** | **repeat_171036**  **repeat_174020**  **repeat_184855**  **repeat_174021**  **repeat_177395**  **repeat_171682**  **repeat_185520** | Repeat | 1^st^ Intron  2^nd^ intron | 51  80  349  264  118  130  123 |
| ***OsGELP106*** | unclassified retrotransposon | **1** | **repeat_183449** | Repeat | 4^th^ intron | 109 |
| ***OsGELP107*** | putative MITE, MITE-adh, type M-like  putative MITE, Castaway-like  putative MITE, Castaway-like | **3** | **repeat_176348**  **repeat_172000**  **repeat_172000** | Repeat | 1^st^ intron | 202  54  199 |
| ***OsGELP109*** | unclassified transposon  MULE04 (MULE04) TI0007117 | **2** | **repeat_174677**  **mcgill_packs_822** | Repeat  pack_mule | 2^nd^ intron  6^th^ exon and  6^th^ intron | 73  720 |
| ***OsGELP111*** | putative MITE, Pangrangja-like  putative MITE, MITE-adh, type A-like  putative MITE, p-SINE1-like | **3** | **repeat_150003**  **repeat_158317**  **repeat_161574** | Repeat | 2^nd^ intron | 228  236  59 |
| ***OsGELP112*** | putative MITE, MITE-adh, type A-like | **1** | **repeat_158471** | Repeat | 3’UTR | 241 |
| ***OsGELP113*** | putative MITE, MITE-adh, type B-like | **1** | **repeat_8597** | Repeat | 3’UTR | 335 |
| ***OsGELP114*** | putative MITE, MITE-adh-5-like | **1** | **repeat_7027** | Repeat | 3^rd^ intron | 78 |
