# Supplementary material for: Multifunctionality and diversity of GDSL esterase/lipase gene family in rice (Oryza sativa L. japonica) genome: new insights from bioinformatics analysis
Source: BMC Genomics. 2012 Jul 15;13:309. doi: 10.1186/1471-2164-13-309 (PMC3412167; doi:10.1186/1471-2164-13-309)
Supplement: Additional file 9 — The 18 OsGELP proteins that were excluded from phylogenetic analysis. The GDSL esterase/lipase gene names, protein length, and the presence of five strictly conserved residues Ser-Gly-Asn-Asp-His in conserved blocks I, II, III, and V for 18 excluded genes are given. The presence of the consensus GDSL blocks is indicated by filled coloured boxes, and blank boxes display the absence of consensus alignment between them and other OsGELP proteins. [file 1471-2164-13-309-S9.doc]

**Additional file 9. Eighteen OsGELP proteins that were excluded from phylogenetic analysis.**

|  | **Gene Name** | **Size aa** | **Block I**  **Serine**  **S** | **Block II**  **Glycine**  **G** | **Block III**  **Asparagine**  **N** | **Block V**  **Aspartate**  **D** | **Block V**  **Histidine**  **H** | **MSU Osa1 Release 6.1 Gene Product Name** |
| --- | --- | --- | --- | --- | --- | --- | --- | --- |
| 1 | ***OsGELP1*** | 239 |  |  |  |  |  | GDSL-like lipase/acylhydrolase, putative, expressed |
| 2 | ***OsGELP13*** | 305 |  |  |  |  |  | GDSL-like lipase/acylhydrolase, putative, expressed |
| 3 | ***OsGELP19*** | 311 |  |  |  |  |  | GDSL-like lipase/acylhydrolase, putative |
| 4 | ***OsGELP21*** | 296 |  |  |  |  |  | GDSL-like lipase/acylhydrolase, putative, expressed |
| 5 | ***OsGELP28*** | 209 |  |  |  |  |  | GDSL-motif lipase/hydrolase protein, putative |
| 6 | ***OsGELP43*** | 323 |  |  |  |  |  | GDSL-like lipase/acylhydrolase, putative, expressed |
| 7 | ***OsGELP47*** | 277 |  |  |  |  |  | GDSL-like lipase/acylhydrolase, putative |
| 8 | ***OsGELP48*** | 340 |  |  |  |  |  | GDSL-like lipase/acylhydrolase, putative |
| 9 | ***OsGELP58*** | 323 |  |  |  |  |  | GDSL-like lipase/acylhydrolase, putative, expressed |
| 10 | ***OsGELP62*** | 212 |  |  |  |  |  | GDSL-like lipase/acylhydrolase, putative, expressed |
| 11 | ***OsGELP69*** | 285 |  |  |  |  |  | GDSL-like lipase/acylhydrolase, putative |
| 12 | ***OsGELP71*** | 322 |  |  |  |  |  | GDSL-like lipase/acylhydrolase, putative, expressed |
| 13 | ***OsGELP76*** | 190 |  |  |  |  |  | GDSL-like lipase/acylhydrolase, putative |
| 14 | ***OsGELP79*** | 341 |  |  |  |  |  | GDSL-like lipase/acylhydrolase, putative, expressed |
| 15 | ***OsGELP106*** | 339 |  |  |  |  |  | GDSL-like lipase/acylhydrolase, putative, expressed |
| 16 | ***OsGELP109*** | 271 |  |  |  |  |  | GDSL-like lipase/acylhydrolase, putative, expressed |
| 17 | ***OsGELP112*** | 261 |  |  |  |  |  | GDSL-like lipase/acylhydrolase, putative, expressed |
| 18 | ***OsGELP113*** | 281 |  |  |  |  |  | GDSL-like lipase/acylhydrolase, putative, expressed |
