# Supplementary material for: Multifunctionality and diversity of GDSL esterase/lipase gene family in rice (Oryza sativa L. japonica) genome: new insights from bioinformatics analysis
Source: BMC Genomics. 2012 Jul 15;13:309. doi: 10.1186/1471-2164-13-309 (PMC3412167; doi:10.1186/1471-2164-13-309)
Supplement: Additional file 10 — Physiological role, properties, and putative functions of plant GDSL esterases/lipases . The name, accession number, properties, and putative functions, as well as general biological roles of 24 plant GDSL esterases/lipases, whose putative functions have been elucidated recently and were adjoined into the original rice OsGELP family NJ tree, are listed. The coloured table divides 24 plant GDSL esterase/lipase proteins into three parts according to their major biological roles: secondary metabolism, plant development and morphogenesis, and defence and are shaded in blue, green, and light pink, respectively. In total, 50 OsGELP proteins with their names and percentage of similarity to every plant homolog or ortholog protein, whose function was revealed recently, along with phylogenetic subclade specificity to the tree from Figure 4, are given. [file 1471-2164-13-309-S10.doc]

**Additional file 10.** Physiological role, properties and putative functions of plant GDSL esterase/lipases.

| **Function** | | **Gene name** | **Accession number**  **Swiss-Prot** | **Properties and putative functions** | **Molecular function** | **OsGELP**  **homologs/**  **orthologs** | **Subclade specificity** | **Ref.** |
| --- | --- | --- | --- | --- | --- | --- | --- | --- |
| **Secondary metabolism** | Aryloxyphenoxypropionate (AOPP) metabolism | ***AmGDSH1*** (*Alopecurus myosuroides* hydrolase) | CAG27610 | Activation of AOPP graminicides to bioactive herbicidal acids occurs in crops and weeds via hydrolysis of aryloxyphenoxypropionate (AOPP) herbicide esters precursors by AmGDSH1.  Endogenous metabolism and herbicide bioactivation in crops and weeds. | Hydrolase activity, acting on ester bonds. | 69% ***OsGELP4,***  48% ***OsGELP5,***  56% ***OsGELP12*** | **Ia** | [47] |
|  | Phenylpropanoid metabolism | ***BnSCE3/BnLIP2*** (*Brassica napus* sinapine esterases) | AAX59709 | Hydrolysis of sinapine during seed germination via transformation of 1-O-sinapoyl-b-glucose to sinapoyl-L-malate in the seedlings. Hydrolysis of sinapine used to increase nutritional value of oilseed rape seeds. | Hydrolase activity, acting on ester bonds. | 48% ***OsGELP17,***  56% ***OsGELP18,***  47% ***OsGELP20,***  43% ***OsGELP49,***  42% ***OsGELP111*** | **Ic** | [48] |
|  | Ajmaline metebolism | ***AAE*** (*Rauvolﬁa serpentina* acetylajmalan esterase) | AAW88320 | Deacetylation of acetylnorajmaline leading to ajmaline formation (the last step in ajmaline alkaloid biosynthesis).Acetylated ajmalan alkaloids is a natural substrate for AAE.  Possible role in the late stages of ajmaline formation. | Acetylajmaline esterase activity.  Deacetylation. | 44% ***OsGELP88,***  46% ***OsGELP111*** |  | [49] |
|  | Xyloglucan metabolism | ***AtFXG1*** (*Arabidopsis thaliana* alpha-fucosidase 1) | Q9FXE5 | Hydrolytic activity against the oligosaccharides from xyloglucan XXFG, and 2’-fucosyl-lactitol of the α-l-fucosidase.  Putative role in hydrolysis of xyloglucans (carbohydrates), and a key role in the regulation of the XXFG levels. | Hydrolase activity, acting on ester bonds.  Carbohydrate hydrolysis. | 42% ***OsGELP14,***  42% ***OsGELP15,***  40% ***OsGELP61,***  44% ***OsGELP66,***  40% ***OsGELP80***  Orthologs:  55% ***OsGELP91,***  57% ***OsGELP92*** | **Ie** | [50] |
|  | Cardenolide metabolism. | ***LAE***  (*Digitalis lanata* Ehrh. lanatoside 15’-O-acetylesterase) | CAA09694 | Deacetylation of lanatosides (one of the main type of cardenolides) to the purpureaglycosides by lanatoside 15’-O- acetylesterase (LAE) catalyzis.  Hypothetic role of the cell wall bound LAE protein in cardenolide biosynthesis and transformation. | Hydrolase activity, acting on ester bonds.  Carbohydrate hydrolysis. | 40% ***OsGELP14,***  40% ***OsGELP66,***  61% ***OsGELP80,*** 41% ***OsGELP91,***  43% ***OsGELP92*** |  | [51] |
|  | Acetylcholine metabolism | ***AChE***  (*Macroptilium atropurpureum*  Siratro  acetylcholinesterase ) | BAG09557 | Hydrolytic activity on acetylthiocholine and propionylthiocholine, with high specificity constant s against acetylcholine, thiocholinesters and cholinesters. Possibilities of multiple substrate specificity including other ester compound.  The ACh-mediated system potentially localized in the extracellular region around the plasmodesmatal channel that might conduct cell-to-cell trafficking by a channel gating regulation.  Hypothetical role of the ACh-mediated system in regulation of the opening and/or closing of channels by interaction with morphoregulatory proteins at the cell wall matrix surrounding the plasmodesmata. | Hydrolase activity, acting on ester bonds. | 42% ***OsGELP14,***  40% ***OsGELP15,***  40% ***OsGELP16,***  44% ***OsGELP66,***  48% ***OsGELP91,***  48% ***OsGELP92*** |  | [52] |
|  |  | ***AChE***  (*Zea mays* L*.* acetylcholinesterase ) | Q5FC14 |  |  | 44% ***OsGELP66***  Orthologs:  67% ***OsGELP91,***  80% ***OsGELP92*** |  | [53,  54] |
|  |  | ***AChE***  (*Salicornia europaea* L. acetylcholinesterase) | AB489863 | Increasing of acetylcholinesterase (AChE) activity in the root and the lower part of the stem following salt (Na^+^ and Cl^-^) accumulation during growth of the Salicornia plants.  Putative function of the acetylcholine (ACh)-mediated system in Salicornia in transport of ions (excessive salt) through channels from epidermal cells of roots by cell-to-cell transport, in a manner similar to the animal systems. | Hydrolase activity, acting on ester bonds. | 41% ***OsGELP14,***  42% ***OsGELP66,***  40% ***OsGELP80,***  48% ***OsGELP91,***  53% ***OsGELP92*** |  | [55] |
|  | Lipid catabolism | ***Hev b 13***  (Latex allergen *Hevea brasiliensis* 13) | Q7Y1X1 | Hypothetical may be involved in nodulation. Have lipase and esterase activities. | Hydrolase activity, acting on ester bonds | 45% ***OsGELP14,***  43% ***OsGELP15,***  42% ***OsGELP61,***  43% ***OsGELP66,***  42% ***OsGELP80,***  50% ***OsGELP91,***  54% ***OsGELP92*** |  | [77] |
|  |  | ***ARAB-1***  (Arabidopsis lipase-1) | **Q38894** | A lipolytic activity withTween-80 plate was shown. | Hydrolase activity, acting on ester bonds. | Orthologs:  49% ***OsGELP49,***  45% ***OsGELP50,***  40% ***OsGELP93,***  46% ***OsGELP17,***  42% ***OsGELP18,***  42% ***OsGELP20,***  40% ***OsGELP105*** | **Ic** | [1] |
|  |  | ***CpEst***  *(Carica papaya* esterase) | **P86276** | A lipolytic activity with tributyrin, and vinyl esters. Hypothetica may be involved in plant defence. | Hydrolase activity, acting on ester bonds | 33% ***OsGELP94*** | **IIIf** | [63] |
| **Plant development**  **and morphogenesis** | Plant cutin biosynthesis | ***AgaSGNH***  (*Agave americane* GDSL and SGNH hydrolases) | Q5J7N0 | Extensive expression in the epidermis of the youngest leaf zones, especially active during cutin biosynthesis.  Hypothetical role of an extracellular AgaSGNH protein in metabolism of the epidermis cell wall and/or in the metabolism of plant cuticle. | Hydrolase activity, acting on ester bonds. | 72% ***OsGELP40,*** 69% ***OsGELP45,*** 69% ***OsGELP56,*** 72% ***OsGELP96,*** 72% ***OsGELP108*** |  | [45] |
|  | Plant development at seedling stage and cutin organization | ***WDL1***  (*Oryza sativa* wilted dwarf and lethal 1) | LOC_Os11g48070.1 | The *wilted dwarf and lethal 1* (*wdl1*) T-DNA insertion mutant plant dwarf and die at seedling stage due to the water loss.  Possible involvement in the epidermal cell differentiation, through cutin organization, by providing pre-formed oligomeric esters or inducing modiﬁcation of non-ester cross-links of non-depolymerizable components. | n/a | 38% ***OsGELP9*** | **Ia** | [57] |
|  | Organ development and coleoptile elongation | ***GER1***  (*Oryza sativa* GDSL containing enzyme rice 1) | LOC_Os02g15230.1 | Induction by both red (R) and far-red (FR) light, and jasmonic acid (JA).  Possible role of *GER1* in either biosynthesis of JA itself or in JA signalling response, as well as alternative function in events that regulate these processes. Putative function as a negative regulator of coleoptile elongation in the context of the impact of JA on light signalling. | n/a | 63% ***OsGELP3,***  74% ***OsGELP84,***  51% ***OsGELP85,***  49% ***OsGELP77,***  47% ***OsGELP2,***  47% ***OsGELP63*** | **Ib** | [56] |
|  | Root nodulation | ***ENOD8***  *(Medicago sativa* Early nodulins protein 8) | AAB41547 | The acetylesterase activity on shorter chain aliphatic ester substrates (acetyl and butyryl esters).  Due to the high homology to the cell wall associated proteins ENOD8 substrates could be acetylated oligo- or polysaccharides. | Hydrolase activity, acting on ester bonds. | 44% ***OsGELP66,***  41% ***OsGELP80,***  49% ***OsGELP91,***  52% ***OsGELP92*** | **Ie** | [46] |
|  | Pollination and fertilization | ***EXL4***  (*Arabidopsis thaliana* pollen coat protein extracellular lipase 4) | Q0WUV7 | T-DNA insertion mutant plants reduce expression of EXL4 and shows slower pollen hydration on the stigma and decreased competitiveness in pollination relative to wild type.  Promotion of the initiation of pollen coat (cuticle) hydration. | Hydrolase activity, acting on ester bonds. | 36% ***OsGELP 29,***  36% ***OsGELP 68,***  37% ***OsGELP 97,***  38% ***OsGELP100,***  38% ***OsGELP102,***  38% ***OsGELP107*** | **IIIb** | [43] |
|  |  | ***CDEF1***  (*Arabidopsis thaliana* cuticle destructing factor 1) | Q9SZW7 | The ectopic expression of CDEF1 (under the 35S promoter) causes disruption of cuticle and fusion of plant organs.  Facilitation of the penetration of the stigma by pollen tubes via stigma (cutin) surface destruction (hydrolyzation), and the lateral roots emergence via degradation of cell wall components. | Hydrolase activity, acting on ester bonds. | 40% ***OsGELP67,***  Ortholog:  44% ***OsGELP73*** | **IIIe** | [42] |
|  | Pollinator attraction and prevention of microbial growth within nectar | ***JNP1***  ( *Jacaranda mimosifolia* nectar protein 1) | **B0FTZ8** | The esterase/lipase activities of the heterologously expressed JNP1 from the raw nectar.  Putative role in the hydrolysis of the nectar lipids with the concomitant release of free fatty acids. | Hydrolase activity, acting on ester bonds. | 68% ***OsGELP24,***  50% ***OsGELP67,*** 66% ***OsGELP73,*** 48% ***OsGELP95*** |  | [44] |
| **Defence** | Biotic | ***ESM1*** (*Arabidopsis thaliana* epithiospecifier modifier 1)  ***MVP1*** (*Arabidopsis thaliana* modiﬁed vacuole phenotype1) | Q9LJG3  Q7XA74 | Myrosinase-associated proteins play a role in the myrosinase-glucosinolate metabolic plant defence system hydrolysis. | Aliphatic glucosinolate and aromatic glucosinolate hydrolysis  Hydrolysis of allyl glucosinolate in vitro. | 31% ***OsGELP44,*** 31% ***OsGELP94***  29% ***OsGELP94,***  28% ***OsGELP102*** | **IIId** | [33] |
|  |  |  |  |  |  |  | **IIIf** |  |
|  |  |  |  |  |  |  | **IIIb** |  |
|  |  | ***AtGLIP1***  (*Arabidopsis thaliana* GDSL lipase1) | Q9FLN0 | Local and systemic resistance: disruption of the spore integrity and defence signalling activation.  Activation role for generation and propagation of a systemic signal required for ethylene (ET)-mediated systemic resistance. | Hydrolase activity, acting on ester bonds. | 35% ***OsGELP98*** |  | [35,  36] |
|  |  | ***AtGLIP2*** (*Arabidopsis thaliana* GDSL lipase2) | **Q9SYF0** | Syestmic resistance: gene induced by a salicylic acid (SA), JA and ET.  Resistance to *Erwinia carotovora* via negative regulation of auxin signaling. | Hydrolase activity, acting on ester bonds. Arylesterase activity. | 31% ***OsGELP26,***  32% ***OsGELP29,***  32% ***OsGELP100***  31% ***OsGELP97,***  35% ***OsGELP98,***  31% ***OsGELP87*** |  | [37] |
|  |  |  |  |  |  | 30% ***OsGELP74*** | **IIIc** |  |
|  |  |  |  |  |  | 30% ***OsGELP42,***  31% ***OsGELP90*** | **IIId** |  |
|  |  |  |  |  |  | 32% ***OsGELP67,***  32% ***OsGELP95*** | **IIIe** |  |
|  |  | ***BrSIL1***  (*Brassica rapa* salicylate-induced lipase-like 1 gene) | Q8L8G1 | Systemic acquired resistance (SAR): gene is activated via SA-dependent signalling pathway, in the presence of the non-host pathogen. | Hydrolase activity, acting on ester bonds. | 32% ***OsGELP29,***  34% ***OsGELP87,***  33% ***OsGELP97,***  37% ***OsGELP98*** | **IIIb** | [38] |
|  |  |  |  |  |  | 32% ***OsGELP74*** | **IIIc** |  |
|  |  |  |  |  |  | 33% ***OsGELP42,***  32% ***OsGELP90*** | **IIId** |  |
|  |  |  |  |  |  | 32% ***OsGELP67,***  342% ***OsGELP95*** | **IIIe** |  |
|  |  | ***CaGLIP1***  (*Capsicum annuum* GDSL-type lipase) | Q08ET5 | Systemic acquired resistance (SAR): gene is activated via a SA-dependent signalling pathway, in the presence of pathogen *Xanthomonas campestris* pv. *vesicatoria* (*Xcv*).  Defensive role of *CaGLIP1* gene during early responses to biotic stresses. | Hydrolase activity, acting on ester bonds.  . | 67% ***OsGELP24,***  50% ***OsGELP67,*** 65% ***OsGELP73,*** 48% ***OsGELP95*** |  | [39] |
|  | Abiotic | ***CaGLIP1***  (*Capsicum annuum* GDSL-type lipase) | Q08ET5 | Induction during abiotic stimuli (sodium nitroprusside, methyl viologen, high salt, mannitol-mediated dehydration and wounding).  Defensive role of CaGLIP1 gene during early responses to abiotic stresses. |  |  |  | [39] |
|  |  | ***CaGL1***  (*Capsicum annuum* GDSL-lipase 1) | Q0R4F7 | Induction by methyl jasmonic acid (MeJA), and local/systemic wounding stimuli.  Association with signalling pathway of MeJA and/or early stage of wounding responses through *CaPR-4* expression modulation. | Hydrolase activity, acting on ester bonds. | 67% ***OsGELP24,***  51% ***OsGELP67,*** 66% ***OsGELP73,*** 49% ***OsGELP95*** |  | [40] |
|  |  | ***AtLTL1***  (*Arabidopsis thaliana* Li-tolerant lipase1) | Q9M8Y5 | Induction by LiCl or NaCl, and activation in the presence of SA.  Halotolerance is a general function of AtLTL1 in the Arabidopsis plant. | Hydrolase activity, acting on ester bonds. | Orthologs:  68% ***OsGELP40,*** 64% ***OsGELP45,*** 69% ***OsGELP56,*** 71% ***OsGELP96,*** 66% ***OsGELP108*** | **IIIf** |  |
