# Supplementary material for: Multifunctionality and diversity of GDSL esterase/lipase gene family in rice (Oryza sativa L. japonica) genome: new insights from bioinformatics analysis
Source: BMC Genomics. 2012 Jul 15;13:309. doi: 10.1186/1471-2164-13-309 (PMC3412167; doi:10.1186/1471-2164-13-309)
Supplement: Additional file 11 — Putative conserved motifs predicted in the OsGELP and known plant GDSL esterase/lipase proteins. The consensus sequence, regular expression, amino acid length, number of the OsGELP proteins containing the motif, and E-value of each 45 predicted motifs are given. The overall height of each column in the motif LOGO indicates sequence conservation at that position, whereas the height of symbols within each column presents relative frequency of the corresponding amino acid. GDSL lipase consensus block distribution is as follows: block I is located in motif 3, block II in motif 5, block III in motif 6, and block V in motif 2. Four strictly conserved catalytic residues Ser-Gly-Asn-HisxxAsp from conserved blocks I, II, III, and V are coloured red in regular expression of corresponding motifs. Regular expression pattern sequences that are coloured in blue and green represent possible sequences for secondary structure elements like helix or sheet, respectively. [file 1471-2164-13-309-S11.doc]

**Additional file 11.** Putative conserved motifs predicted in the OsGELP and known plant GDSL esterase/lipase proteins.

| **Motif#** | **Motif consensus sequence** | **E value** | **Regular expression (RE) describing the motif.** | **No. of OsGELP proteins** | **Length (amino acids)** | **Protein secondary structure elements** | **Phylogenetic clades and subclades specificity** |
| --- | --- | --- | --- | --- | --- | --- | --- |
| **1** | 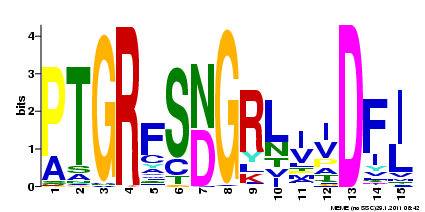 | 1.3e-966 | [PA]TGRFS[ND]G**RL[IV][IV]DF[IL]** | 120 | 15 | α1-helix |  |
| **2** | 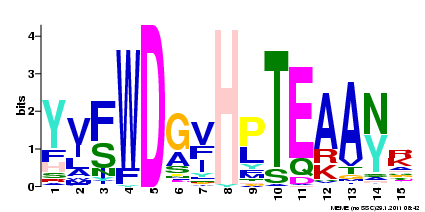 | 1.0e-955 | **YV[FS]WD**G[VF]**H**[PL]T**EAA[NY][KR]** | 116 | 15 | Β6-sheet  L10-loop  α6-helix |  |
| **3** | 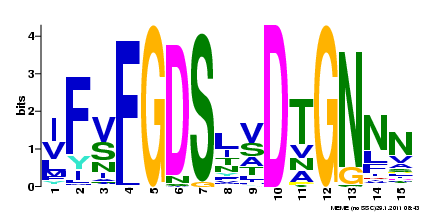 | 2.0e-883 | **[IV]F[VS]FG**D**S**L[VS]DTGNNN | 118 | 15 | β1-sheet  L1-loop |  |
| **4** | 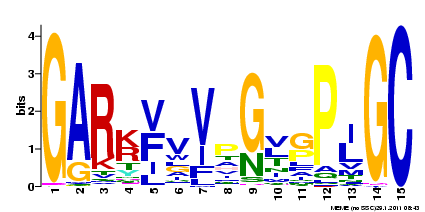 | 6.1e-746 | GA**R[KR][VF]V[VI]P[GN]**[VL][GP]P[IL]GC | 120 | 15 | β4-sheet |  |
| **5** | 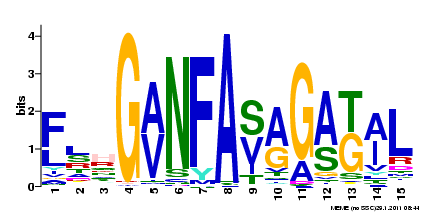 | 1.4e-577 | [FL]LH**G[AV]NFA[SV]**[AG]**G**[AS][TG][AI]L | 111 | 15 | β2-sheet  L3-loop |  |
| **6** | 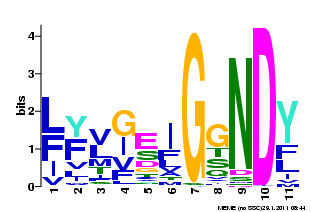 | 8.1e-404 | **[LF][FY][VL][GIV]E**IGG**N**DY | 119 | 11 | β3-sheet |  |
| **7** | 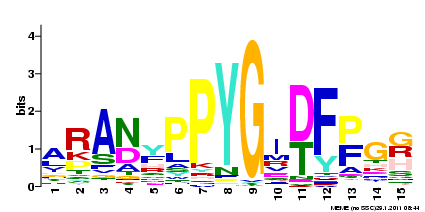 | 5.9e-365 | ARA[ND]YPPYGI[DT]F[PF]G[GRH] | 95 | 15 | L1-loop | Clades II, III |
| **8** | 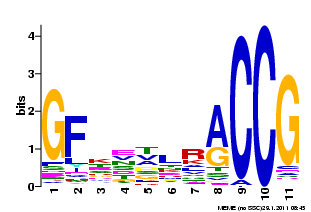 | 4.1e-307 | GFxxxLR[AG]CCG | 114 | 11 | L9-loop |  |
| **9** | 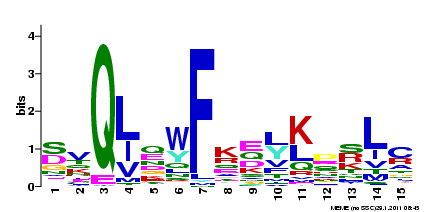 | 4.9e-300 | [SD]VQ[LI]QWFKE**[LY][KL]P[RS]LC** | 92 | 15 | L3-loop  α2-helix |  |
| **10** | 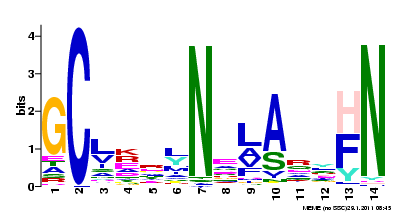 | 5.4e-389 | G**CLKx[LY]NxL[AS]Rx[HF]N** | 109 | 14 | α4-helix |  |
| **11** | 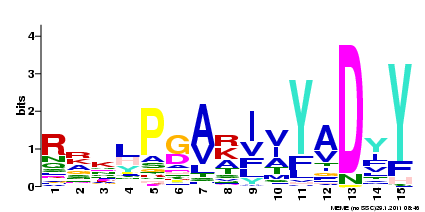 | 1.5e-271 | RxxLP[GD][AV][KR]**[IV][VI]Y[AV]D**YY | 94 | 15 | L8-loop  β5-sheet |  |
| **12** | 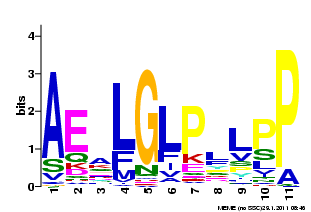 | 2.8e-249 | AEALGLPLLPP | 109 | 11 | L2-loop |  |
| **13** | 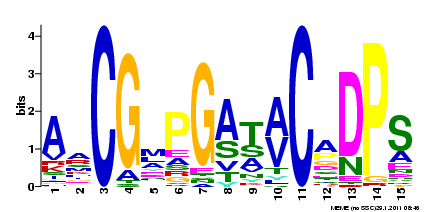 | 1.6e-151 | AxCGMPG[AS]T[AV]CADPS | 38 | 15 | L9-loop | Subclades  Ia, Ib, Ic, Id |
| **14** | 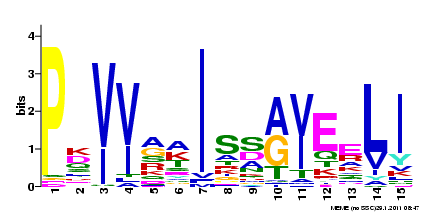 | 1 1.3e-147 | **Px[VI][VI]AAISS[AG]**[VI]EELI | 48 | 15 | α3-helix | Clade I:  Subclades  Ia, Ib, Ic, Id, Ie |
| **15** | 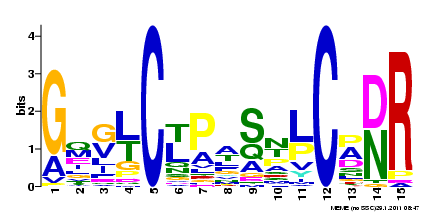 | 1.3e-098 | GQ[GV][LT]C[LT]PA[SQ]N[LP]C[AP][DN]R | 33 | 15 | L9-loop | Subclades  IIId, IIIe, IIIf |
| **16** | 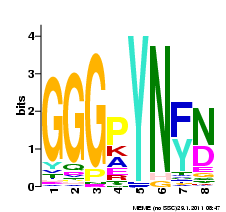 | 8.7e-078 | GGGPYN[FY][ND] | 49 | 8 | L9-loop | Clade I:  Subclades  Ia, Ib, Ic, Id,Ie |
| **17** | 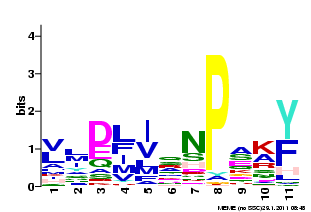 | 3.3e-070 | **[VL]x[DE]L[IV]xNP**xK[YF] | 83 | 11 | α5-helix L9-loop |  |
| **18** | 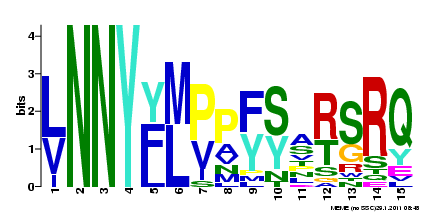 | 3.6e-069 | [LV]NNY[FYL][LM][PV]P[FY][SY]A[RT][SG]**RQ** | 18 | 15 | L5-loop  α3-helix | Subclades  IIIe, IIIf |
| **19** | 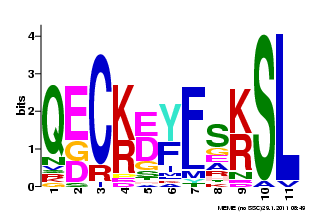 | 3.5e-065 | **Q[EDG]C[KR][DE][YF][FL]**S[KR]SL | 24 | 11 | α2-helix | Subclades  Ia, Ib, Ic |
| **20** | 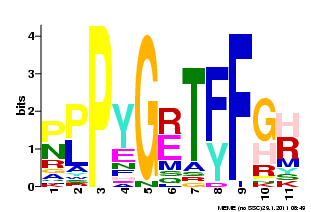 | 1.4e-058 | [  [PN][PL]PYG[ER]T[FY]F[GH][HR] | 24 | 11 | L1-loop | Clade I:  Subclades  Ia, Ib, Ic, Id,Ie |
| **21** | 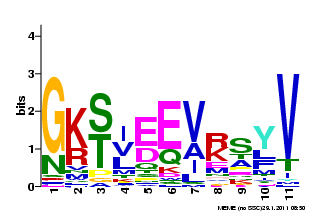 | 1.8e-032 | G[KR][ST][IVL][ED]E**V[RK]SYV** | 37 | 11 | L5-loop  α3-helix | Clade I:  Subclades  Ia, Ib, Ic, Id,Ie |
| **22** | 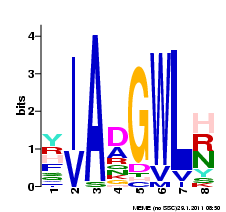 | 1.8e-032 | Y[IV]A[DA]GWL[HNR] | 25 | 8 | C terminal loop | Subclades  Ia, Ib, Ic |
| **23** | 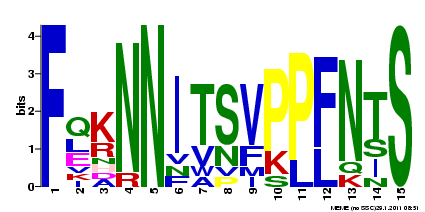 | 6.6e-028 | F[QEL][KR]NNI[TV][SN][VF][PK][PL][FL]N[TIS]S | 10 | 15 | L3-loop | Subclade Ia, |
| **24** | 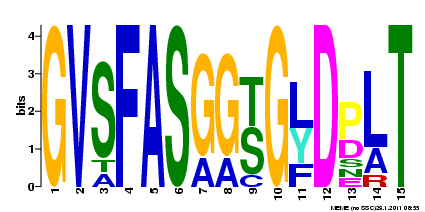 | 1.8e-028 | **GVSFAS[GA][GA][ST]**G[LYF]D[PD][LA]T | 9 | 15 | β2-sheet  L3-loop | Subclade IIIb |
| **25** | 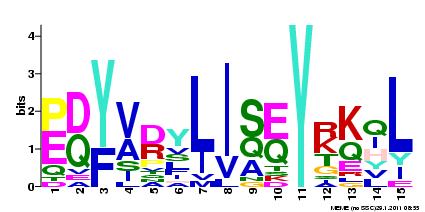 | 3.0e-027 | **[EP][DQ][YF][VA][DR]YLI[SQ][EQ]Y[KR]**[KQ]QL | 16 | 15 | α3-helix | Subclades  IIId, IIIe, IIIf |
| **26** | 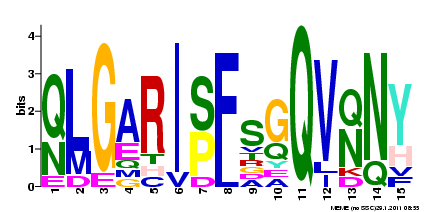 | 1.2e-025 | [QN][LM]G[AE]RI[SP]**[FL]S[GQ]QV[NQ][NQ][YH]** | 10 | 15 | L3-loop  α2-helix | Subclade IIIe |
| **27** | 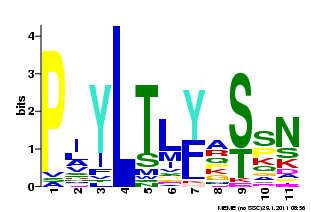 | 4.7e-026 | **PIYLTL[YF]**A[ST]SN | 27 | 11 | α4-helix | Subclades  Ia, Ib, Ic |
| **28** | 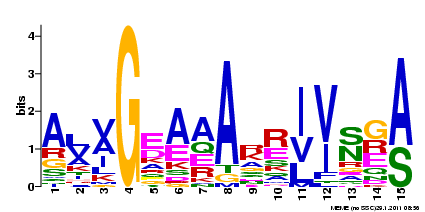 | 6.2e-026 | A[LV][VA]GE**[AE]AAR[RE][IVL][VI**][SN][GER][AS] | 28 | 15 | L3-loop  α2-helix  L4-loop | Subclades  IIIb, IIIc, IIIf |
| **29** | 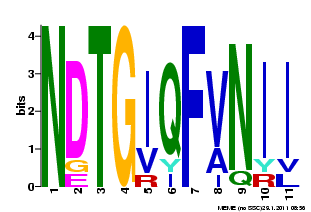 | 1.5e-023 | NDTG[IV]QF[VA]NII | 9 | 11 | L3-loop | Subclade IIIf |
| **30** | 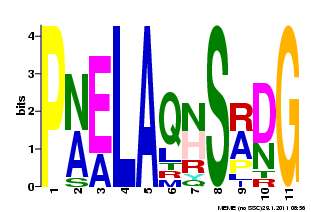 | 1.3e-023 | **P[NA][EA]LA**Q[HN]S[AR][DN]G | 12 | 11 | α4-helix | Subclades  IIIe, IIIf |
| **31** | 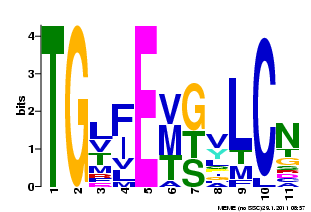 | 2.0e-023 | TG[LTV][FI]E[MVT][GST][VY]LCN | 15 | 11 | L9-loop | Subclades  IIIb, IIIc |
| **32** | 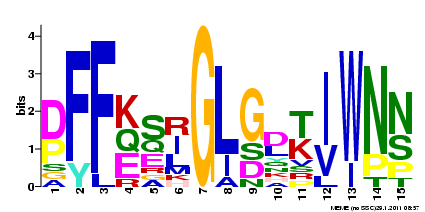 | 2.0e-019 | [DP]FF[KEQ]S[IR]GLGD[TK][IV]WN[NS] | 11 | 15 | L3-loop | Subclade Ib |
| **33** | 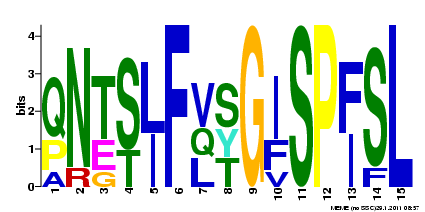 | 4.0e-015 | [QP]N[TE][ST][LI]F[VLQ][STY]G[IF]SP[FI]SL | 7 | 15 | L3-loop | Subclade Ie |
| **34** | 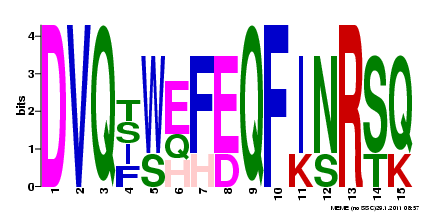 | 2.6e-013 | **DVQ[FIST][WS][EHQ][FH][ED]QF[IK][NS]R[ST][QK]** | 4 | 15 | α2-helix | Subclade Ie |
| **35** | 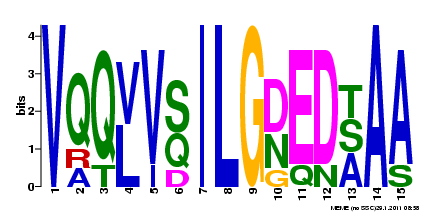 | 8.6e-012 | **VQQ[LV]V[SQ]IL**G[DN]ED[AST]AA | 6 | 15 | α2-helix  L4-loop | Subclade IIIe |
| **36** | 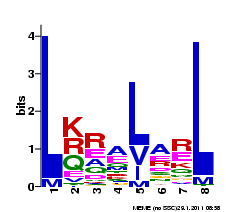 | 3.3e-011 | **L[KQR]RALA[RE]L** | 54 | 8 | α4-helix |  |
| **37** | 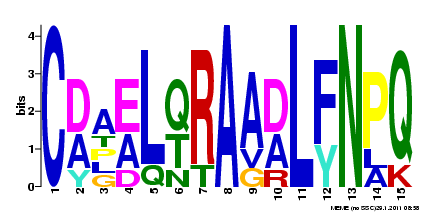 | 1.8e-007 | **C[DA]A[EA]L[QT]RAA[DA]L[FY]NPQ** | 6 | 15 | α4-helix | Subclade IIIf |
| **38** | 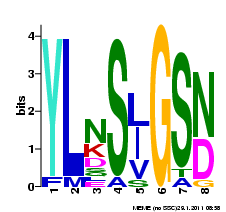 | 5.4e-006 | YL[NK]S[ILV]GS[ND] | 14 | 8 | L2-loop | Subclade Ie |
| **39** | 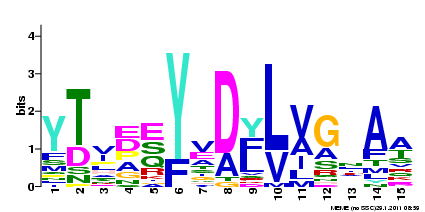 | 6.3e-005 | **Y[TD]V[DE][EQS][YF]V[DA][FYL][LV][VA]GxAA** | 24 | 15 | α3-helix | Subclades  IIIb, IIIc |
| **40** | 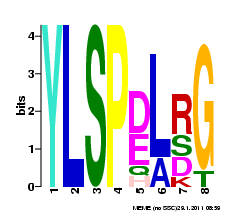 | 1.0e-003 | YLSP[DE][LA][RDS]G | 9 | 8 | L2-loop | Subclade IIIf |
| **41** | 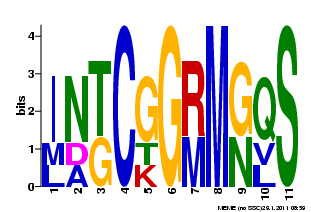 | 3.4e-003 | [ILM][NAD][TG**]C[GKT]G[RM]M**[GN][QLV]S | 5 | 11 | α2-helix | Specific to OsGELP18, OsGELP20 |
| **42** | 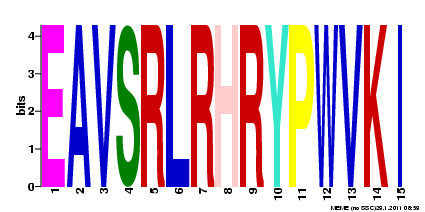 | 3.5e-003 | EAVSRLRHRYP**WVKI** | 2 | 15 | L8-loop  β5-sheet | Specific to OsGELP10, OsGELP11 |
| **43** | 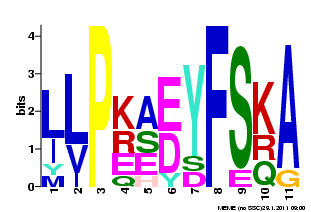 | 5.0e-003 | **[LI][LV]P[KER][AES][ED]YF**S[KQR]A | 8 | 11 | α2-helix | Subclade Ie |
| **44** | 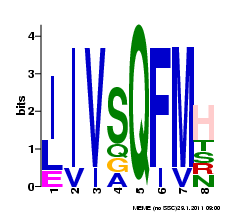 | 8.0e-002 | [IL]IVSQFMH | 7 | 8 | C terminal loop | Subclade IIIf |
| **45** | 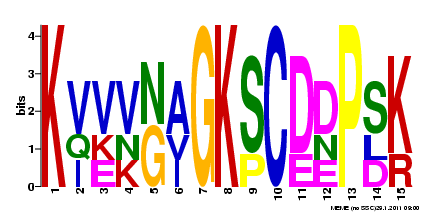 | 9.1e-002 | K[VIQ][VEK][VKN][GN][AIV]GK[SP]C[DE][DEN]P[SDL][KR] | 4 | 15 | L9-loop | Subclade Ie |
