# Supplementary material for: Multifunctionality and diversity of GDSL esterase/lipase gene family in rice (Oryza sativa L. japonica) genome: new insights from bioinformatics analysis
Source: BMC Genomics. 2012 Jul 15;13:309. doi: 10.1186/1471-2164-13-309 (PMC3412167; doi:10.1186/1471-2164-13-309)

Additional file 12. Differential expression of the rice *OsGELP* genes in response to plant hormone cytokinin.

A.

Clade	Gene name	*Trans*-zeatin (seedling root)		Kinetin (seedling)		BAP (seedling)	
		qPCR	GENEVESTIGATOR microarray	qPCR	GENEVESTIGATOR microarray	qPCR	GENEVESTIGATOR microarray
Clade I	*OsGELP5*	1.40	1.04				
Clade I	*OsGELP12*	1.33	-1.23				
Clade I	*OsGELP85*	1.13	2.03				
Clade I	*OsGELP77*	1.76	-1.21				
Clade I	*OsGELP2*	1.33	-1.2				
Clade I	*OsGELP88*					3.38	4.41
Clade I	*OsGELP49*	1.23	2.08				
Clade I	*OsGELP50*	1.39	1.73			5.93	2.18
Clade I	*OsGELP17*	1.24	-1.96				
Clade I	*OsGELP15*	2.35	2.05				
Clade I	*OsGELP61*	1.14	-2.00				
Clade III	*OsGELP92*	1.86	1.12	1.25	-1.2		
Clade III	*OsGELP100*	1.30	-1				
Clade III	*OsGELP74*	1.82	1.07				
Clade III	*OsGELP90*	0.95	3.43				
Clade III	*OsGELP44*	1.11	-1.1				
Clade III	*OsGELP24*	1.54	1.1				

B.

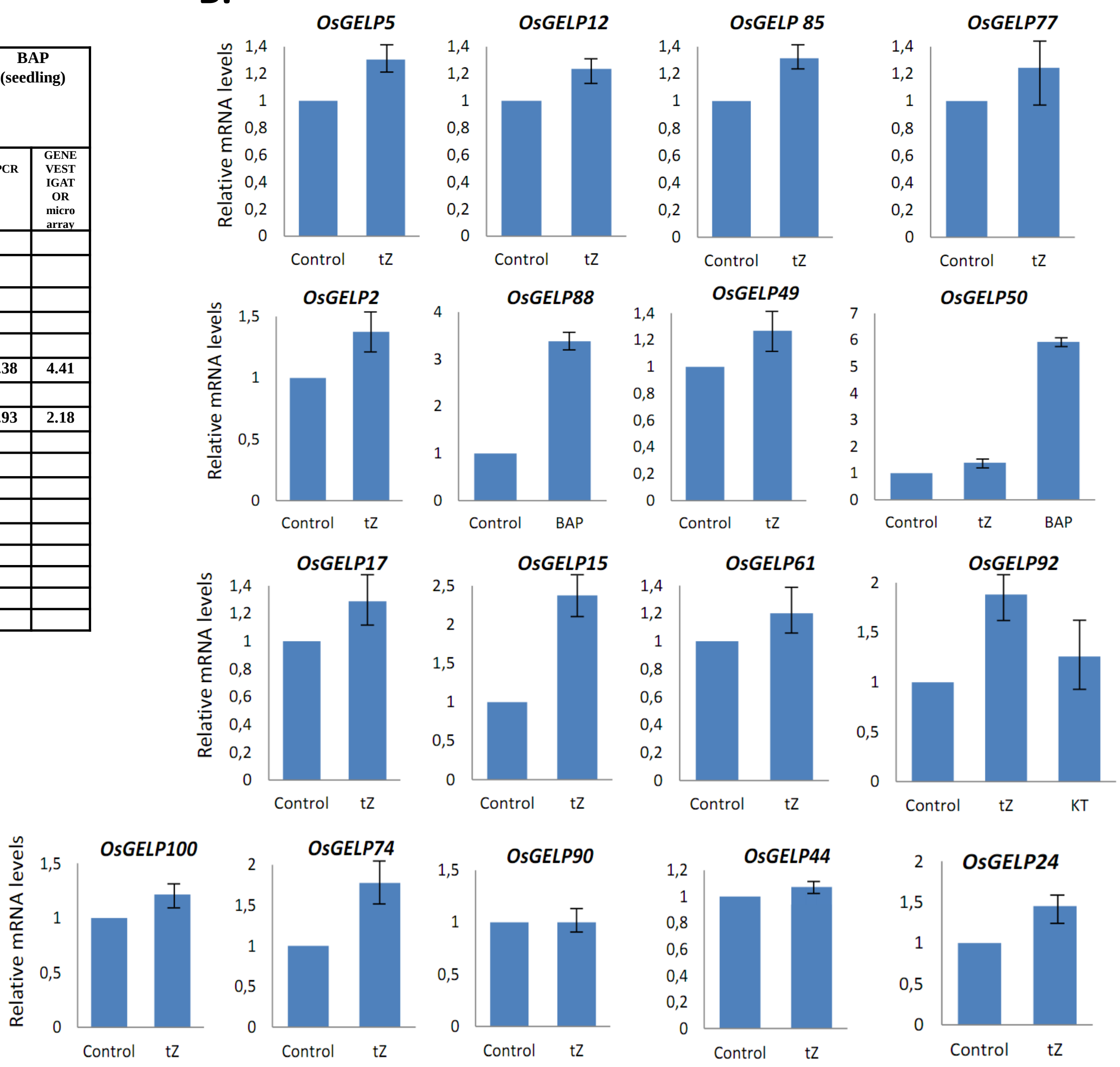

Supplement: Additional file 12 — Differential expression of rice OsGELP genes in response to plant hormone cytokinin. A. Comparison of the fold expression difference for the 17 representative genes under cytokinin (tZ, BAP, and KT) treatment for results from the real-time PCR, and the microarray data obtained from Genevestigator database are given. B. Real-time PCR analysis of representative OsGELP genes and their differential expression during cytokinin (tZ, BAP, and KT) treatment are shown. The mRNA levels for each gene in different tissue samples were calculated relative to its expression in control seedlings. The error bars represent standard deviation. [file 1471-2164-13-309-S12.pdf]
