# Supplementary material for: Multifunctionality and diversity of GDSL esterase/lipase gene family in rice (Oryza sativa L. japonica) genome: new insights from bioinformatics analysis
Source: BMC Genomics. 2012 Jul 15;13:309. doi: 10.1186/1471-2164-13-309 (PMC3412167; doi:10.1186/1471-2164-13-309)
Supplement: Additional file 13 — The rice GDSL esterase/lipase genes excluded from the general list of the OsGELP candidates. The locus ID, ORF length, predicted protein length, the presence of GDSL-lipase domain with confidence (E-value), description, and cDNA support of all 19 excluded genes are given. [file 1471-2164-13-309-S13.doc]

**Additional file 13.** Rice GDSL esterase/lipase genes excluded from the general list of the *OsGELP*s candidates.

| **Locus ID** | **Open reading frame length (bp)** | **Predicted protein**  **length**  **(aa)** | **Pham E -value of PF00657** | **MSU Osa1 Release 6.1 annotation** | **cDNA accession number** |
| --- | --- | --- | --- | --- | --- |
| [Os01g12304](http://rice.plantbiology.msu.edu/cgi-bin/ORF_infopage.cgi?&orf=LOC_Os01g12304) | 597 | 199 | 1.3e-15 | esterase, putative, expressed | [AK071394](http://www.ncbi.nlm.nih.gov/entrez/viewer.fcgi?db=nucleotide&val=AK071394) |
| [Os01g12340](http://rice.plantbiology.msu.edu/cgi-bin/ORF_infopage.cgi?&orf=LOC_Os01g12340) | 6243 | 2081 | 1.20e-01 | retrotransposon protein, putative, unclassified | n/a |
| [Os01g13680](http://rice.plantbiology.msu.edu/cgi-bin/ORF_infopage.cgi?&orf=LOC_Os01g13680) | 240 | 80 | 1e-07 | hypothetical protein | [AK062747](http://www.ncbi.nlm.nih.gov/nuccore/32972765?report=genbank) |
| [Os01g32630](http://rice.plantbiology.msu.edu/cgi-bin/ORF_infopage.cgi?&orf=LOC_Os01g32630) | 4779 | 1593 | 3e-22 | retrotransposon protein, putative, unclassified | n/a |
| [Os01g46260](http://rice.plantbiology.msu.edu/cgi-bin/ORF_infopage.cgi?&orf=LOC_Os01g46260) | 537 | 179 | 1.7e-06 | expressed protein | [AK111325](http://www.ncbi.nlm.nih.gov/entrez/viewer.fcgi?db=nucleotide&val=AK111325) |
| [Os02g26080](http://rice.plantbiology.msu.edu/cgi-bin/ORF_infopage.cgi?&orf=LOC_Os02g26080) | 435 | 145 | 4.7e-05 | anther-specific proline-rich protein APG precursor, putative | n/a |
| [Os02g26090](http://rice.plantbiology.msu.edu/cgi-bin/ORF_infopage.cgi?&orf=LOC_Os02g26090) | 510 | 170 | 7e-09 | anther-specific proline-rich protein APG, putative | n/a |
| [Os02g39150](http://rice.plantbiology.msu.edu/cgi-bin/ORF_infopage.cgi?&orf=LOC_Os02g39150) | 513 | 171 | 8e-04 | hypothetical protein | n/a |
| [Os03g07070](http://rice.plantbiology.msu.edu/cgi-bin/ORF_infopage.cgi?&orf=LOC_Os03g07070) | 270 | 90 | 4.9e-05 | hypothetical protein | n/a |
| [Os03g24960](http://rice.plantbiology.msu.edu/cgi-bin/ORF_infopage.cgi?&orf=LOC_Os03g24960) | 1200 | 400 | **1.6e-08** | ulp1 protease family, C-terminal catalytic domain containing protein | n/a |
| [Os03g38355.1](http://rice.plantbiology.msu.edu/cgi-bin/ORF_infopage.cgi?orf=13103.m04162) | 255 | 85 | **1.9e-08** | GSDL-motif lipase, putative | n/a |
| [Os03g38400](http://rice.plantbiology.msu.edu/cgi-bin/ORF_infopage.cgi?&orf=LOC_Os03g38400) | 342 | 114 | 1e-16 | GDSL-motif lipase/hydrolase family protein, putativ | n/a |
| [Os05g43080](http://rice.plantbiology.msu.edu/cgi-bin/ORF_infopage.cgi?&orf=LOC_Os05g43080) | 222 | 74 | 2e-08 | GDSL-motif lipase/hydrolase-like protein, putative | n/a |
| [Os06g03890](http://rice.plantbiology.msu.edu/cgi-bin/ORF_infopage.cgi?&orf=LOC_Os06g03890) | 546 | 182 | 1e-03 | alpha-L-fucosidase 3 precursor, putative | n/a |
| [Os06g03900](http://rice.plantbiology.msu.edu/cgi-bin/ORF_infopage.cgi?&orf=LOC_Os06g03900) | 612 | 204 | 2.1e-09 | esterase precursor, putative, expressedsed | n/a |
| [Os06g24420](http://rice.plantbiology.msu.edu/cgi-bin/ORF_infopage.cgi?&orf=LOC_Os06g24420) | 3405 | 1135 | 2.1e-20 | transposon protein, putative, CACTA, En/Spm sub-class | n/a |
| [Os09g03490](http://rice.plantbiology.msu.edu/cgi-bin/ORF_infopage.cgi?&orf=LOC_Os09g03490) | 399 | 133 | 4e-10 | carboxylic ester hydrolase, putative | n/a |
| [Os10g09130](http://rice.plantbiology.msu.edu/cgi-bin/ORF_infopage.cgi?&orf=LOC_Os10g09130) | 453 | 151 | 5e-12 | retrotransposon protein, putative, unclassified | n/a |
| [Os11g19690](http://rice.plantbiology.msu.edu/cgi-bin/ORF_infopage.cgi?&orf=LOC_Os11g19690) | 645 | 215 | 3.4e-08 | transposon protein, putative, CACTA, En/Spm sub-class | n/a |
