# Supplementary material for: Multifunctionality and diversity of GDSL esterase/lipase gene family in rice (Oryza sativa L. japonica) genome: new insights from bioinformatics analysis
Source: BMC Genomics. 2012 Jul 15;13:309. doi: 10.1186/1471-2164-13-309 (PMC3412167; doi:10.1186/1471-2164-13-309)
Supplement: Additional file 14 — Motifs represent 13 highly conserved OsGELP protein alignment blocks used for phylogenetic analysis. The consensus sequence, regular expression, length (amino acids), number of the OsGELP proteins containing the motif, and E-value of each of predicted motifs are given. The overall height of each column in the motif LOGO indicates sequence conservation at that position, whereas the height of symbols within each column presents relative frequency of the corresponding amino acid. GDSL lipase consensus block distribution is as follows: motif 3 is located in block I, motif 5 is in block II, motif 6 is in block III, and motif 2 is in block V. Four strictly conserved catalytic residues Ser-Gly-Asn-HisxxAsp from conserved blocks I, II, III, and V are coloured red in the regular expression of representative motif. Regular expression pattern sequences that are coloured in blue and green represent possible sequences for secondary structure elements like helix or sheet, respectively. [file 1471-2164-13-309-S14.doc]

**Additional file 14.** Motifs that represent thirteen highly conserved GELP protein’s alignment blocks, which employed for phylogenetic analysis.

| **No.** | **Motif #** | **Motif consensus sequence** | **E value** | **Regular expression (RE) describing the motif.** | **No. of OsGELP proteins** | **Length (amino acids)** | **Protein secondary structure elements** |
| --- | --- | --- | --- | --- | --- | --- | --- |
| 1 | **1** | 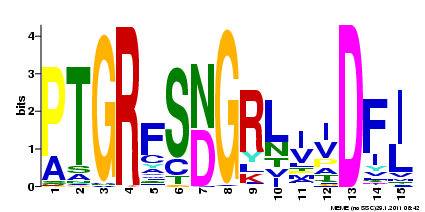 | 1.3e-966 | [PA]TGRFS[ND]G**RL[IV][IV]DF[IL]** | 120 | 15 | α1-helix |
| 2 | **2** | 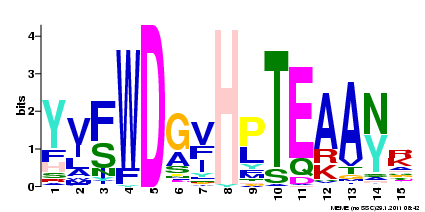 | 1.0e-955 | **YV[FS]WD**G[VF]**H**[PL]T**EAA[NY][KR]** | 116 | 15 | α6-helix |
| 3 | **3** | 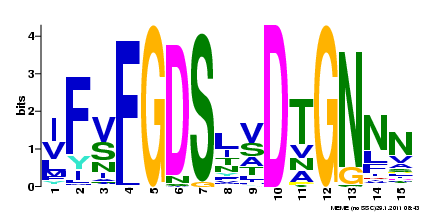 | 2.0e-883 | **[IV]F[VS]FG**D**S**L[VS]DTGNNN | 118 | 15 | β1-sheet  L1-loop |
| 4 | **4** | 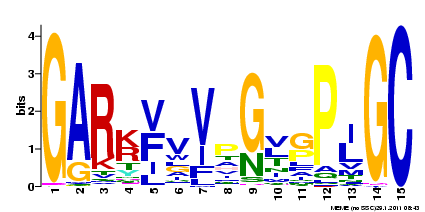 | 6.1e-746 | GA**R[KR][VF]V[VI]P[GN]**[VL][GP]P[IL]GC | 120 | 15 | β4-sheet |
| 5 | **5** | 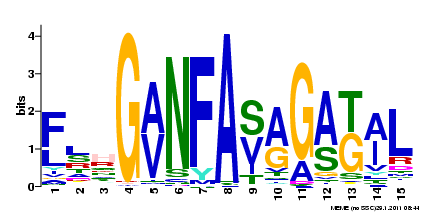 | 1.4e-577 | [FL]LH**G[AV]NFA[SV]**[AG]**G**[AS][TG][AI]L | 111 | 15 | β2-sheet  L3-loop |
| 6 | **6** | 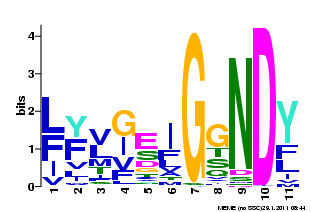 | 8.1e-404 | **[LF][FY][VL][GIV]E**IGG**N**DY | 119 | 11 | β3-sheet |
| 7 | **7** | 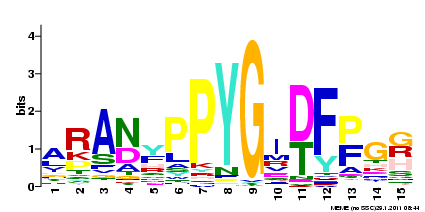 | 5.9e-365 | ARA[ND]YPPYGI[DT]F[PF]G[GRH] | 95 | 15 | L1-loop |
| 8 | **8** | 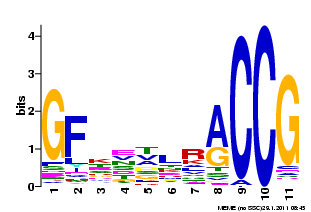 | 4.1e-307 | GFxxxLR[AG]CCG | 114 | 11 | L9-loop |
| 9 | **10** | 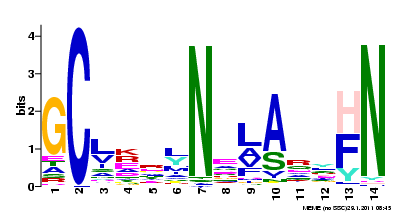 | 5.4e-389 | G**CLKx[LY]NxL[AS]Rx[HF]N** | 109 | 14 | α4-helix |
| 10 | **11** | 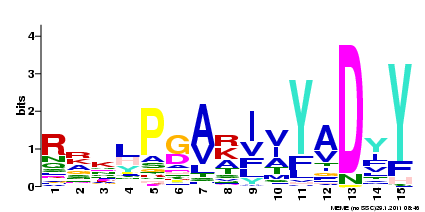 | 1.5e-271 | RxxLP[GD][AV][KR]**[IV][VI]Y[AV]D**YY | 94 | 15 | L8-loop  β5-sheet |
| 11 | **12** | 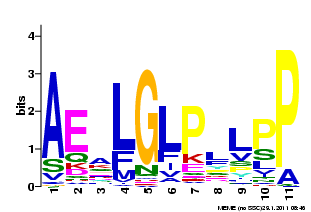 | 2.8e-249 | AEALGLPLLPP | 109 | 11 | L2-loop |
| 12 | **17** | 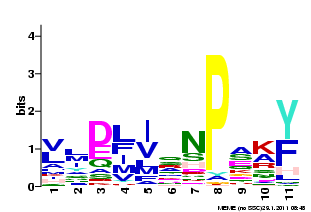 | 3.3e-070 | **[VL]x[DE]L[IV]xNP**xK[YF] | 83 | 11 | L9-loop |
| 13 | **20** | 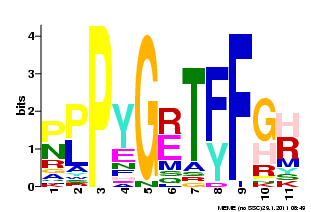 | 1.4e-058 | [  [PN][PL]PYG[ER]T[FY]F[GH][HR] | 24 | 11 | L1-loop |
| 14 | **22** | 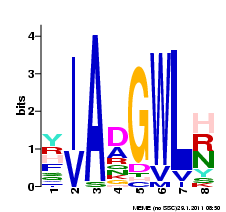 | 1.8e-032 | Y[IV]A[DA]GWL[HNR] | 25 | 8 | C terminal loop |
| 15 | **24** | 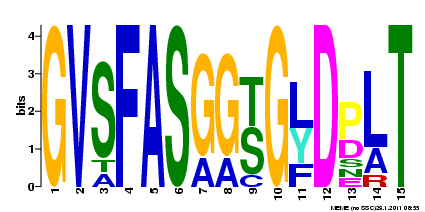 | 1.8e-028 | **GVSFAS[GA][GA][ST]G**[LYF]D[PD][LA]T | 9 | 15 | β2-sheet  L3-loop |
| 16 | **27** | 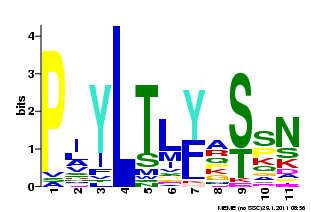 | 4.7e-026 | **PIYLTL[YF]**A[ST]SN | 27 | 11 | α4-helix |
| 17 | **30** | 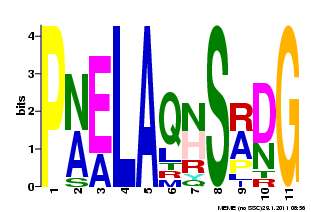 | 1.3e-023 | **P[NA][EA]LA**Q[HN]S[AR][DN]G | 12 | 11 | α4-helix |
| 18 | **36** | 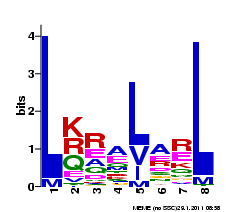 | 3.3e-011 | **L[KQR]RALA[RE]L** | 54 | 8 | α4-helix |
| 19 | **37** | 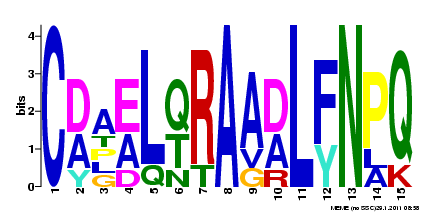 | 1.8e-007 | **C[DA]A[EA]L[QT]RAA[DA]L[FY]NPQ** | 6 | 15 | α4-helix |
| 20 | **38** | 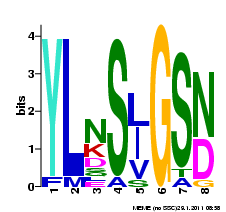 | 5.4e-006 | YL[NK]S[ILV]GS[ND] | 14 | 8 | L2-loop |
| 21 | **40** | 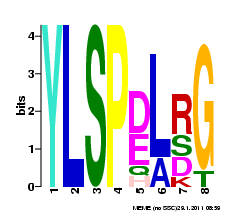 | 1.0e-003 | YLSP[DE][LA][RDS]G | 9 | 8 | L2-loop |
| 22 | **42** | 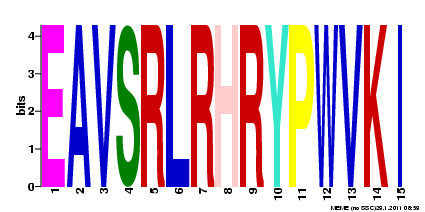 | 3.5e-003 | EAVSRLRHRYP**WVKI** | 2 | 15 | L8-loop  β5-sheet |
| 23 | **44** | 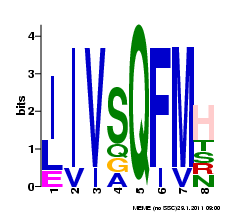 | 8.0e-002 | [IL]IVSQFMH | 7 | 8 | C terminal loop |
