# Supplementary material for: Multifunctionality and diversity of GDSL esterase/lipase gene family in rice (Oryza sativa L. japonica) genome: new insights from bioinformatics analysis
Source: BMC Genomics. 2012 Jul 15;13:309. doi: 10.1186/1471-2164-13-309 (PMC3412167; doi:10.1186/1471-2164-13-309)
Supplement: Additional file 15 — Primer sequences used for real-time PCR analysis. The OsGELP gene names and sequences of PCR primers used in the quantitative RT PCRs to verify gene expression levels are listed. [file 1471-2164-13-309-S15.docx]

**Additional file 15.** Primer sequences used for real-time PCR analysis.

| **Gene name** | **Locus** | **Primer sequence** |
| --- | --- | --- |
| ***OsGELP2*** | Os01g11620 | 5'- CTTCCCGCTGTACCTGACGCTGTA-3' |
|  |  | 5'-CCTGGTCGTAGAAGTTGCCGTACAT-3’ |
| ***OsGELP5*** | Os01g11700 | 5'-GCGCGGCCACACAGAAACTCAT-3’ |
|  |  | 5'-GGTTGTGCTCCATGGACAGCA-3’ |
| ***OsGELP12*** | Os01g12320 | 5'-CGGACATCATCCGTACCATCTCCA-3’ |
|  |  | 5'-TTGTGCAGCGTCGCTACCTCGT-3’ |
| ***OsGELP15*** | Os01g22660 | 5'-GCTCTTGGCTGCCTTCCTCAGAA-3’ |
|  |  | 5'-TCGTACTTGAGAGGGAACAGATCGG-3’ |
| ***OsGELP17*** | [Os01g42730](http://rice.plantbiology.msu.edu/cgi-bin/ORF_infopage.cgi?&orf=LOC_Os01g42730) | 5'-TCCTTGCCCCACTCCAGTTTG-3’ |
|  |  | 5'-AGGTTGCCTCCGTGAAGTGGA-3’ |
| ***OsGELP24*** | Os01g52770 | 5'-AGCAGTTCGCCGACTCGCTCAT-3’ |
|  |  | 5'-AGATCTGGATGGCGGAGTCGATG-3’ |
| ***OsGELP44*** | [Os02g50690](http://rice.plantbiology.msu.edu/cgi-bin/ORF_infopage.cgi?&orf=LOC_Os02g50690).1 | 5'-ATGAGGTACTCCGTCGGCAGCT-3’ |
|  |  | 5'-TCCCAGAACAGGTACCCGTTGC-3’ |
| ***OsGELP49*** | [Os03g25030](http://rice.plantbiology.msu.edu/cgi-bin/ORF_infopage.cgi?&orf=LOC_Os03g25030) | 5'-GTCAAGTCCGCAAGACTATGATGCC-3’ |
|  |  | 5'-ACGGATCTGGTGCAACATGCGT-3’ |
| ***OsGELP50*** | Os03g25040 | 5-CACGACGTCGAACCTGTGCA-3’ |
|  |  | 5'-TGTGTGGGTATGATCAGGCTGT-3’ |
| ***OsGELP61*** | [Os05g06710](http://rice.plantbiology.msu.edu/cgi-bin/ORF_infopage.cgi?&orf=LOC_Os05g06710) | 5'- CGAATTACAACAAGGCTGCACGG-3’ |
|  |  | 5'- GCACACCATCAACGGCCACTT-3’ |
| ***OsGELP74*** | [Os06g05550](http://rice.plantbiology.msu.edu/cgi-bin/ORF_infopage.cgi?&orf=LOC_Os06g05550) | 5'- CTTCAAAAACACCGTGGCACAACT-3’ |
|  |  | 5'- CGAGTCGACGGTGACGTTCATCTT-3’ |
| ***OsGELP77*** | Os06g06250 | 5'-ACCCGCTTCAACACCCTCTCCT-3’ |
|  |  | 5'-GCAGCACGCCCTCAAATTCGT-3’ |
| ***OsGELP85*** | [Os06g34120](http://rice.plantbiology.msu.edu/cgi-bin/ORF_infopage.cgi?&orf=LOC_Os06g34120) | 5'-TTGGATGCTTCCCCTTGTACCTGAC-3’ |
|  |  | 5'-TGCCGCCTTGAAGTAGTCACCAT-3’ |
| ***OsGELP88*** | [Os06g47910](http://rice.plantbiology.msu.edu/cgi-bin/ORF_infopage.cgi?&orf=LOC_Os06g47910) | 5'-ATGCAGAAGCTGAGACGAAGATGA-3’ |
|  |  | 5'-CCAACGTTTGACCGTCCATCTTAT-3’ |
| ***OsGELP90*** | [Os06g50950](http://rice.plantbiology.msu.edu/cgi-bin/ORF_infopage.cgi?&orf=LOC_Os06g50950) | 5’-TCTTCCTCCTCGGCGTCGTCAA-3’ |
|  |  | 5’-CGACCAGCCCCACGTTGATGAT-3 |
| ***OsGELP92*** | Os07g39750 | 5'-CCTCGTCATCGACTTCATCGCTG-3’ |
|  |  | 5'-GACTGCACGTCCAAGGAAATGG-3’ |
| ***OsGELP100*** | Os09g36880 | 5’-TTCCTCGCCAATCTAGCCTCTGAT-3’ |
|  |  | 5'-TGCTCCAGCTTGGCGTTGAACA-3’ |
| ***ACT1*** | AK100267 | 5’-CAGCCACACTGTCCCCATCTA-3’ |
|  |  | 5’-AGCAAGGTCGAGACGAAGGA-3’ |
